# Design, Synthesis and Bioactivities of Novel Isoxazole-Containing Pyrazole Oxime Derivatives

**DOI:** 10.3390/molecules22122000

**Published:** 2017-11-27

**Authors:** Hong Dai, Wei Yao, Yuan Fang, Siyu Sun, Yujun Shi, Jia Chen, Guoqing Jiang, Jian Shi

**Affiliations:** 1 College of Chemistry and Chemical Engineering, Nantong University, Nantong 226019, China; daihong_2015@aliyun.com (H.D.); yaowei265688@126.com (W.Y.); sunsiyu95@163.com (S.S.); 15642891665@163.com (J.C.); 2 School of Environmental and Chemical Engineering, Jiangsu University of Science and Technology, Zhenjiang 212003, China; fyuan6586@aliyun.com; 3 Analysis and Testing Center, Nantong University, Nantong 226019, China

**Keywords:** isoxazole, pyrazole oxime, synthesis, bioactivity

## Abstract

In this study, in order to find novel biologically active pyrazole oxime derivatives, twenty-eight new pyrazole oxime compounds containing a substituted isoxazole ring were synthesized and evaluated for their acaricidaland insecticidal activities. Bioassays exhibited that some target compounds indicated good acaricidal and insecticidal activities against *Tetranychus cinnabarinus*, *Aphis medicaginis*, *Mythimna separata*, and *Nilaparvata lugens*. Especially, compounds **9c**, **9h**, **9u**, and **9v** showed 100.00%, 90.56%, 90.78%, and 90.62% insecticidal activities against *A. medicaginis* at the concentration of 20 μg/mL, respectively, compounds **9k** and **9u** had 70.86% and 100.00% insecticidal activities against *M. separata* at 20 μg/mL, respectively.

## 1. Introduction

In the past few decades, heterocycles play a significant role in the research of agricultural and medicinal chemistry. The isoxazole skeleton, a crucial type of nitrogen-containing heterocycle, has been used in pesticide and drug design because of their various biological activities, such as insecticidal [1,2,3], herbicidal [4,5,6], fungicidal [7], antiviral [8,9,10], and anticancer activities [11]. Recently, Yu et al. obtained a series of 3,4,5-trisubstituted isoxazoles that were showing good insecticidal activities [12]. More recently, Sun et al. reported several series of isoxazole compounds carrying benzoylurea moiety displaying perfect insecticidal activities [13]. The widespread use of isoxazole-based compounds as a scaffold in the field of agriculture and medicine research endows the isoxazole ring as an important structural class.

**Figure 1:**
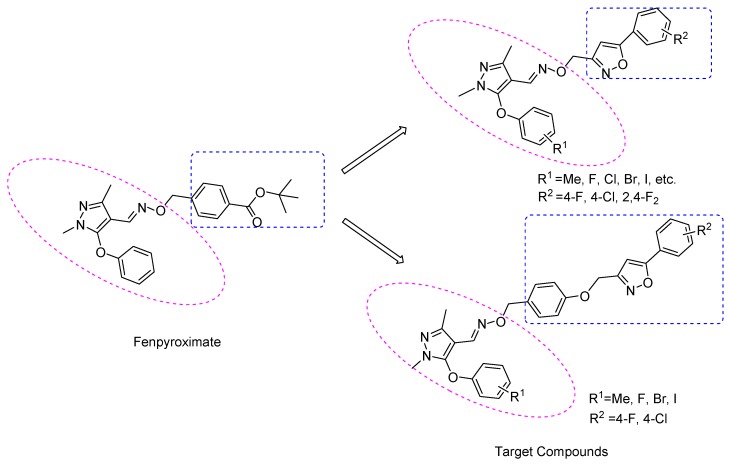
The design of the target molecules.

On the other hand, pyrazole oxime derivatives are one of the hotspots in the design of new drugs due to their broad spectrum of insecticidal [14,15], acaricidal [16], fungicidal [17], anti-TMV [18], and antitumor activities [19]. In particular, the insecticidal and acaricidal activities have been widely investigated for their potential applications in agricultural production. For instance, Fenpyroximate (Figure 1), an excellent acaricide containing a pyrazole oxime moiety, is used to control some phytophagous mites, such as Tetranychus urticae Koch and Polyphagotarsonemus latus Banks [20,21]. Recently, Dai et al. reported that some pyrazole oximes possessed interesting insecticidal and acaricidal activity through modification of the esterified group of Fenpyroximate with thiazolylmethoxy or thiadiazolylmethoxy unit [22,23]. Wang and co-workers synthesized and evaluated the insecticidal activity of a series of pyrazole oxime ethers containing oxazole ring and found that some compounds showed good insecticidal properties against Aphis craccivora and *Nilaparvata lugens* [24]. Very recently, Dai and co-workers also obtained some pyrazole oximes owning wonderful acaricidal and insecticidal activities against *Tetranychus cinnabarinus*, *Aphis medicaginis* and *Nilaparvata lugens* by substituting the esterified aryl group of Fenpyroximate with oxadiazole ring [25]. This gave a great impetus to the search for biologically active molecules carrying the pyrazole oxime subunit.

Encouraged by the aforementioned facts, we envisioned that the introduction of a substituted isoxazole pharmacophore into the parent pyrazole oxime scaffold might produce some new compounds with multiple biological activities. In this study, we describe the design and synthesis of a number of novel pyrazole oxime derivatives bearing isoxazole ring (Figure 1). Moreover, all of the new compounds were tested for their acaricidal and insecticidal activities. 

## 2. Results and Discussion

### 2.1. Chemistry

**Scheme 1:**
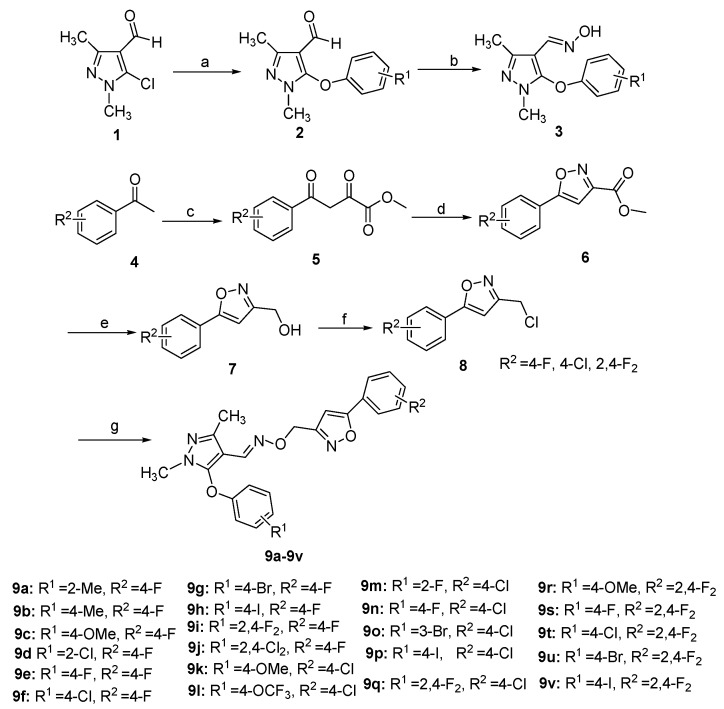
Synthesis of the title compounds **9a**–**9v**. *Reagents and conditions:* (a) substituted phenol, KOH, DMF or DMSO, 40 °C, 2–4 h, 105 °C, 8–24 h, 52–76% for **2**; (b) NH_2_OH·HCl, KOH, CH_3_OH, reflux, 7–22 h, 61–73% for **3**; (c) dimethyl oxalate, CH_3_ONa, CH_3_OH, 60 °C, 6–8 h, 53–65% for **5**; (d) NH_2_OH·HCl, CH_3_OH, 60 °C, 7–10 h, 55–60% for **6**; (e) LiAlH_4_, THF, 0 °C, 3–6 h, 70–76% for **7**; (f) SOCl_2_, DMF, CH_2_Cl_2_, 0 °C, 4–7 h, 73–80% for **8**; (g) compound **3**, K_2_CO_3_, Cs_2_CO_3_, CH_3_CN, reflux, 8–19 h, 47–63% for **9**.

**Scheme 2:**
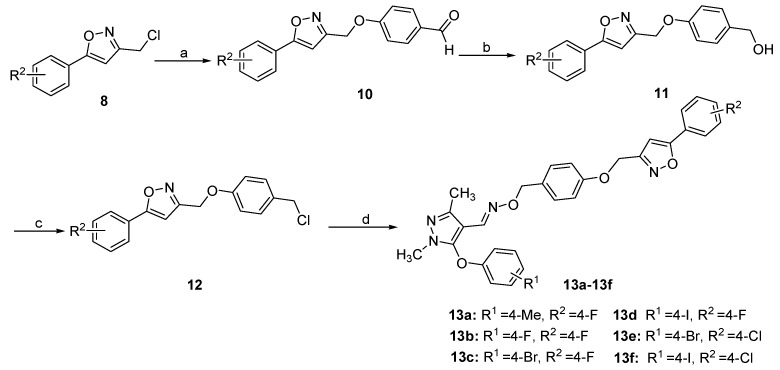
Synthesis of the title compounds **13a**–**13f**. *Reagents and conditions:* (a) 4-hydroxybenzaldehyde, Cs_2_CO_3_, CH_3_CN, reflux, 5 h, 71–77% for **10**; (b) LiAlH_4_, THF, 0 °C, 30 min, 68–72% for **11**; (c) SOCl_2_, DMF, CH_2_Cl_2_, 0 °C, 6–8 h, 62–65% for **12**; and, (d) compound **3**, K_2_CO_3_, CH_3_CN, reflux, 10–15 h, 46–56% for **13**.

The general synthetic route of the title compounds **9a**–**9v** was depicted in Scheme 1. The key intermediates **3** were conveniently synthesized by two steps from 1,3-dimethyl-5-chloro-1*H*-pyrazole-4-carbaldehyde (**1**). The condensation of compound **1** with various substituted phenols under basic conditions gave compounds **2**, which were easily transformed into intermediates **3** by treatment with hydroxylamine hydrochloride using potassium hydroxide as the base. Intermediate 3-chloromethyl-5-substituted phenyl isoxazole (**8**) was prepared from compound **4**. Compound **4** was easily reacted with dimethyl oxalate under basic conditions to obtain compound **5**. Further reaction with hydroxylamine hydrochloride afforded compound **6** successfully. Next, a reaction with LiAlH_4_ was undertaken to form compound **7** in good yields. Further chlorination of compound **7** provided intermediate **8** smoothly by the addition of some drops of *N*,*N*-dimethylformamide. Finally, pyrazole oximes **3** were admixed with 3-chloromethyl-5-substituted phenyl isoxazole (**8**) in acetonitrile using potassium carbonate as alkali and cesium carbonate as catalyst to afford corresponding compounds **9a**–**9v** in satisfactory yields (Scheme 1). The synthetic route for the target compounds **13a**–**13f** is shown in Scheme 2. The important intermediates **12** were smoothly prepared in three steps from compound **8**. The condensation of compound **8** with 4-hydroxybenzaldehyde under basic condition afforded compounds **10**, which were transformed to intermediates **11** by treatment with LiAlH_4_. Then, compounds **11** reacted with SOCl_2_ to give intermediates **12** conveniently. At last, intermediates **12** were treated with compound **8** in acetonitrile using potassium carbonate as alkali to obtain corresponding pyrazole oximes derivatives **13a**–**13f** in good yields (Scheme 2). The target compounds **9a**–**9v** and **13a**–**13f** were effectively characterized by ^1^H-NMR, ^13^C-NMR, and elemental analyses.

### 2.2. Biological Activities

The synthesized compounds **9a**–**9v** and **13a**–**13f** were evaluated for insecticidal activities against *Aphis medicaginis*, *Mythimna separata* and *Nilaparvata lugens*, and acaricidal activity against *Tetranychus cinnabarinus* using known procedures, and Chlorantraniliprole, Pyridalyl, Abamectin and Fenpyroximate were used as the positive controls, respectively. As indicated in Table 1, some title compounds showed moderate to good acaricidal activity against *T. cinnabarinus* at a concentration of 500 μg/mL. The mortalities of compounds **9e** and **9q** were 80.46% and 50.38%, respectively. Besides acaricidal potencies, most title compounds displayed wonderful insecticidal activities against *A. medicaginis* at a dosgae of 500 μg/mL, for example, compounds **9b**, **9c**, **9d**, **9e**, **9g**, **9h**, **9i**, **9j**, **9k**, **9q**, **9r**, **9s**, **9t**, **9u**, and **9v** all possessed 100.00% inhibition rates, respectively, which were equal to that of the control Chlorantraniliprole. Furthermore, some aimed compounds exhibited potent insecticidal activities against *A. medicaginis* when the dosage was lowered to 100 μg/mL. For instance, compounds **9b**, **9c**, **9d**, **9g**, **9h**, **9i**, **9j**, **9u**, and **9v** all owned 100.00% insecticidal activity against *A. medicaginis*, respectively, which were comparable to that of the control Chlorantraniliprole. Even when the dosage was reduced to 20 μg/mL, some compounds still had satisfactory insecticidal activity against *A. medicaginis*, and the mortalities of compounds **9c**, **9h**, **9u,** and **9v** were 100.00%, 90.56%, 90.78%, and 90.62%, respectively. From the above insecticidal activity data, we can find that the substituents (R^1^) on the phenyl ring may have an impact on the activities. When R^2^ is 4-fluoro or 2,4-difluoro, the substituent (R^1^) at 4-position of phenyl ring was methoxy (**9c**), halogen (**9g**, **9h**, **9u**, and **9v**), or 2,4-position of phenyl ring was fluoro or chloro (**9i** and **9j**), it was more favorable to the insecticidal activity against *A. medicaginis* at the dosage of 20 μg/mL. Table 2 demonstrated that most target compounds showed excellent larvicidal activity against *M. separata* at a concentration of 500 μg/mL. Moreover, some of them had moderate to good larvicidal activity against *M. separata* when the concentration came to 100 μg/mL, among these derivatives, compounds **9c**, **9k**, and **9u** all exhibited 100.00% inhibition rates, respectively, which were comparable to that of the control Pyridalyl. When the concentration arrived at 20 μg/mL, compounds **9k** and **9u** still indicated potential inhibitory activities against *M. separata,* with the inhibition rates being 70.86% and 100.00%, respectively. When R^2^ is 4-fluoro or 2,4-difluoro, the substituent (R^1^) at 4-position of phenyl ring was methoxy (**9k**) or bromo (**9u**), it was more favorable to the insecticidal activity against *M. separata* at 20 μg/mL. Interestingly, some designed compounds possessed wonderful inhibitory activities against *N. lugens* besides good insecticidal activities against *A. medicaginis* and *M. separata*. Among them, compounds **9c**, **9d**, **9i**, **9k**, **9r**, **9s**, and **9t** all had 100.00% inhibition rates against *N. lugens* at 500 μg/mL. When the dosage was reduced to 100 μg/mL, some title compounds were still active against *N. lugens*, especially, the inhibition rates of compounds **9e** and **9g** were 75.23% and 75.76%. From the data shown in Table 1 and Table 2, we found that the structure-insecticidal activity relationship of some obtained compounds against *N. lugens* is similar to structure-insecticidal activity relationship of some compounds against *A. medicaginis*. When R^2^ is 4-fluoro, the substituent (R^1^) was 4-fluoro (**9e**) or 4-bromo (**9g**), it was more advantageous to increase the insecticidal activity against *N. lugens* at 100 μg/mL than other substituents. The data presented in Table 1 and Table 2 also displayed that compounds **9c**, **9g**, **9h**, **9i**, **9j**, **9k**, **9r**, **9s**, and **9t** exhibited exciting insecticidal effects against *N. lugens* and *M. separata* beyond wonderful insecticidal activities against *A. medicaginis* at the dosage of 500 μg/mL. At the same time, compound **9e** had good acaricidal activity against *T. cinnabarinus* besides potent insecticidal activities against *A. medicaginis*, *M. separata,* and *N. lugens* at 500 μg/mL. From the data listed in Table 1 and Table 2, we can find that some benzyloxy-linked isoxazole derivatives also possessed good insecticidal activities against *A. medicaginis*, *M. separata,* and *N. lugens* at the dosage of 500 μg/mL. For example, compounds **13c** and **13f** both had 100.00% insecticidal activities against *A. medicaginis*, which were similar to that of the control Chlorantraniliprole. Compounds **13a**, **13b**, **13c**, **13e**, and **13f** all displayed 100.00% insecticidal activities against *M. separata*, respectively, which were equal to that of the control Pyridalyl. In addition, compound **13d** indicated 100.00% inhibitory activity against *N. lugens*, which was near to that of the control Abamectin. All of the above data implied that the bioactivity spectrum of pyrazole oxime derivatives was significantly improved by introducing the important isoxazole ring. This research indicated that these target compounds may function as potential lead structures for the discovery of novel pesticides in future.

## 3. Experimental Section

### 3.1. Chemistry

#### 3.1.1. General Procedures

All of the reagents were chemically pure and solvents were dried according to standard methods. ^1^H-NMR and ^13^C-NMR spectra were obtained on a Bruker AV400 spectrometer (400 MHz, ^1^H; 100 MHz, ^13^C, Bruker, Billerica, MA, USA) in CDCl_3_ with tetramethylsilane as the internal standard. The melting points were determined on an X-4 binocular microscope melting point apparatus (Beijing Tech Instrument Co., Beijing, China) and are uncorrected. Elemental analyses were determined on a Yanaco CHN Corder MT-3 elmental analyzer (Yanaco, Kyoto, Japan). The reactions were monitored by analytical thin-layer chromatography (TLC) with ultraviolet (UV) light and TLC was carried out on silica gel GF_254_. The intermediate 5-chloropyrazole aldehyde **1** was synthesized according to the reported procedure [26]. The intermediates **5** and **6** were prepared by the literature method [27]. 

#### 3.1.2. General Procedure for the Preparation of **2**

To a well stirred solution of substituted phenol (30 mmol) in DMF or DMSO (30 mL), KOH was added (40 mmol) at room temperature. The resulting mixture was heated to 40 °C for 2–4 h, and then compound **1** (20 mmol) was added thereto. The reaction solution was heated to 105 °C for 8–24 h. After being cooled to room temperature, the mixture was poured into water and extracted with ethyl acetate (3 × 50 mL). The combined extracts were dried over anhydrous magnesium sulfate, filtered, and evaporated to afford intermediate **2**, with yields ranging from 52% to 76% [25].

#### 3.1.3. General Procedure for the Preparation of **3**

To a solution of hydroxylamine hydrochloride (30 mmol) in anhydrous methanol (60 mL) at room temperature, was added KOH (40 mmol) in portions, and the mixture was stirred at room temperature for 20 min. To the above solution was added intermediate **2**, the reaction mixture was heated to reflux for 7–22 h. After being cooled to room temperature, the mixture was poured into water (100 mL), and the solid precipitate was filtered, washed with water, and dried to give corresponding 5-substituted phenoxy pyrazole oximes **3**, with yields ranging from 61% to 73% [25].

#### 3.1.4. General Procedure for the Preparation of **7**

To a well stirred cold (0 °C) solution of intermediate **6** (4 mmol) and THF (60 mL), was added LiAlH_4_ (10 mmol) in three portions and the reaction mixture was stirred at 0 °C for 3–6 h. To the above solution, was added ice water (40 mL). After the solid precipitate was filtered, the filtrate was extracted with ethyl acetate (3 × 50 mL). The combined extracts were dried over anhydrous sodium sulfate, filtered, and concentrated to produce compound **7**, with yields ranging from 70% to 76% [28].

#### 3.1.5. General Procedure for the Preparation of **8**

To a well stirred cold (0 °C) solution of compound **7** (20 mmol) in CH_2_Cl_2_ (50 mL), was added dropwise a mixture of thionyl chloride (40 mmol) in CH_2_Cl_2_ (15 mL). Then, several drops of DMF was added thereto. The resulting mixture was stirred at 0 °C for 4–7 h. To the above solution, was added ice water (50 mL), and pH value of the mixture was adjusted to 6 by saturated sodium bicarbonate solution. The separated organic layer was dried over anhydrous sodium sulfate, filtered, and concentrated to afford the corresponding compound **8**, with yields ranging from 73% to 80% [28].

#### 3.1.6. General Procedure for the Preparation of **9a**–**9v**

To a mixture of intermediate **8** (4 mmol), compound **3** (5 mmol), and potassium carbonate (12 mmol) in acetonitrile (30 mL) at room temperature, was added cesium carbonate (1 mmol). The resulting mixture was heated to reflux for 8–19 h. The reaction mixture was allowed to cool at room temperature and filtered. The solvent was evaporated under reduced pressure, and the residue was admixed with water (50 mL) and extracted with ethyl acetate (3 × 50 mL). The combined organic layer was washed with water (3 × 20 mL), dried over anhydrous sodium sulfate, filtered, and concentrated. The residue was purified by silica gel column chromatography using a mixture of petroleum ether and ethyl acetate as an eluent to produce the target compounds **9a**–**9v**, with yields ranging from 47% to 63%. Pyrazole oxime derivatives **9a**–**9v** were novel, and the physical and spectral data for these compounds are listed below. ^1^H-NMR and ^13^C-NMR spectra are provided in the Supplementary Materials.

*Data for* **9a**. Yellow solid, yield 51%, m.p.: 93–94 °C. ^1^H-NMR (400 MHz, DMSO-*d*_6_): *δ* 7.87–7.91 (m, 2H, Ar-H), 7.75 (s, 1H, CH=N), 6.98–7.40 (m, 5H, Ar-H), 6.80 (s, 1H, Isoxazole-H), 6.56 (d, *J* = 8.0 Hz, 1H, Ar-H), 5.00 (s, 2H, CH_2_), 3.55 (s, 3H, N-CH_3_), 2.32 (s, 3H, CH_3_), 2.24 (s, 3H, CH_3_). ^13^C-NMR (100 MHz, CDCl_3_): *δ* 168.9, 165.0, 162.5, 162.1, 154.9, 148.4, 147.0, 141.7, 131.6, 127.9, 127.1, 126.7, 123.8, 123.6, 116.3, 116.0, 113.3, 99.5, 98.9, 67.0, 34.1, 16.1, 14.7. Anal. Calcd for C_23_H_21_FN_4_O_3_: C, 65.70; H, 5.03; N, 13.33. Found: C, 65.56; H, 5.16; N, 13.18.

*Data for* **9b**. White solid, yield 53%, m.p.: 96–97 °C. ^1^H-NMR (400 MHz, CDCl_3_): *δ* 7.83 (s, 1H, CH=N), 7.68 (d, *J* = 8.8 Hz, 2H, Ar-H), 7.43 (d, *J* = 8.4 Hz, 2H, Ar-H), 7.09 (d, *J* = 8.0 Hz, 2H, Ar-H), 6.79 (d, *J* = 8.8 Hz, 2H, Ar-H), 6.47 (s, 1H, Isoxazole-H), 5.10 (s, 2H, CH_2_), 3.60 (s, 3H, N-CH_3_), 2.38 (s, 3H, CH_3_), 2.27 (s, 3H, CH_3_). ^13^C-NMR (100 MHz, CDCl_3_): *δ* 168.8, 162.1, 154.7, 148.3, 146.9, 141.8, 136.2, 133.3, 130.6, 130.4, 129.3, 127.1, 125.9, 118.0, 115.2, 99.8, 99.6, 67.0, 34.3, 20.6, 14.9. Anal. Calcd for C_23_H_21_FN_4_O_3_: C, 65.70; H, 5.03; N, 13.33. Found: C, 65.85; H, 4.90; N, 13.21.

*Data for* **9c**. Yellow solid, yield 55%, m.p.: 104–105 °C. ^1^H-NMR (400 MHz, CDCl_3_): *δ* 7.82 (s, 1H, CH=N), 7.66 (d, *J* = 8.8 Hz, 2H, Ar-H), 7.43 (d, *J* = 8.4 Hz, 2H, Ar-H), 6.80–6.85 (m, 4H, Ar-H), 6.46 (s, 1H, Isoxazole-H), 5.11 (s, 2H, CH_2_), 3.74 (s, 3H, OCH_3_), 3.61 (s, 3H, N-CH_3_), 2.37 (s, 3H, CH_3_). ^13^C-NMR (100 MHz, CDCl_3_): *δ* 168.8, 162.1, 155.8, 150.6, 148.6, 146.9, 141.8, 136.2, 129.3, 127.1, 125.8, 119.8, 116.4, 115.0, 114.9, 99.5, 99.4, 67.0, 55.6, 34.2, 14.8. Anal. Calcd for C_23_H_21_FN_4_O_4_: C, 63.30; H, 4.85; N, 12.84. Found: C, 63.45; H, 4.71; N, 12.96.

*Data for* **9d**. Yellow solid, yield 47%, m.p.: 76–78 °C. ^1^H-NMR (400 MHz, CDCl_3_): *δ* 7.82 (s, 1H, CH=N), 7.68 (d, *J* = 8.4 Hz, 2H, Ar-H), 7.41–7.44 (m, 3H, Ar-H), 7.00–7.15 (m, 2H, Ar-H), 6.70 (d, *J* = 8.4 Hz, 1H, Ar-H), 6.44 (s, 1H, Isoxazole-H), 5.05 (s, 2H, CH_2_), 3.65 (s, 3H, CH_3_), 2.37 (s, 3H, CH_3_). ^13^C-NMR (100 MHz, CDCl_3_): *δ* 168.7, 162.0, 152.1, 147.3, 147.1, 141.3, 136.2, 131.0, 129.3, 128.0, 127.1, 125.9, 124.6, 122.8, 115.5, 99.7, 99.6, 67.0, 34.3, 14.5. Anal. Calcd for C_22_H_18_ClFN_4_O_3_: C, 59.94; H, 4.12; N, 12.71. Found: C, 59.78; H, 4.25; N, 12.85. 

*Data for* **9e**. Yellow oil, yield 50%. ^1^H-NMR (400 MHz, DMSO-*d*_6_): *δ* 7.89–7.92 (m, 2H, Ar-H), 7.85 (s, 1H, CH=N), 7.14–7.41 (m, 4H, Ar-H), 6.98–7.01 (m, 2H, Ar-H), 6.86 (s, 1H, Isoxazole-H), 5.02 (s, 2H, CH_2_), 3.56 (s, 3H, N-CH_3_), 2.24 (s, 3H, CH_3_). ^13^C-NMR (100 MHz, CDCl_3_): *δ* 169.1, 165.0, 162.5, 161.9, 160.0, 157.6, 152.5, 148.8, 148.0, 147.1, 141.3, 127.9, 123.8, 116.7, 116.3, 116.1, 99.8, 98.9, 67.1, 34.4, 14.6. Anal. Calcd for C_22_H_18_F_2_N_4_O_3_: C, 62.26; H, 4.27; N, 13.20. Found: C, 62.10; H, 4.41; N, 13.33.

*Data for* **9f**. White solid, yield 49%, m.p.: 116–118 °C. ^1^H-NMR (400 MHz, DMSO-*d*_6_): *δ* 7.88–7.92 (m, 3H, CH=N and Ar-H), 7.36–7.41 (m, 4H, Ar-H), 6.98 (d, *J* = 8.0 Hz, 2H, Ar-H), 6.85 (s, 1H, Isoxazole-H), 5.02 (s, 2H, CH_2_), 3.56 (s, 3H, N-CH_3_), 2.24 (s, 3H, CH_3_). ^13^C-NMR (100 MHz, CDCl_3_): *δ* 169.0, 165.0, 162.5, 161.9, 155.2, 147.4, 147.1, 141.3, 129.9, 128.9, 127.9, 127.8, 123.7, 116.7, 116.3, 98.9, 98.6, 67.1, 34.3, 14.5. Anal. Calcd for C_22_H_18_ClFN_4_O_3_: C, 59.94; H, 4.12; N, 12.71. Found: C, 60.07; H, 4.02; N, 12.57.

*Data for* **9g**. White solid, yield 52%, 114–115 °C. ^1^H-NMR (400 MHz, CDCl_3_): *δ* 7.84 (s, 1H, CH=N), 7.72–7.75 (m, 2H, Ar-H), 7.40 (d, *J* = 9.2 Hz, 2H, Ar-H), 7.16 (t, *J* = 8.8 Hz, 2H, Ar-H), 6.78 (d, *J* = 8.8 Hz, 2H, Ar-H), 6.40 (s, 1H, Isoxazole-H), 5.08 (s, 2H, CH_2_), 3.60 (s, 3H, CH_3_), 2.37 (s, 3H, CH_3_). ^13^C-NMR (100 MHz, CDCl_3_): *δ* 169.0, 165.0, 162.5, 161.9, 155.7, 147.1, 141.3, 132.9, 127.9, 127.8, 123.8, 117.1, 116.3, 116.2, 116.1, 99.9, 98.9, 67.1, 34.3, 14.6. Anal. Calcd for C_22_H_18_BrFN_4_O_3_: C, 54.45; H, 3.74; N, 11.54. Found: C, 54.30; H, 3.88; N, 11.63.

*Data for* **9h**. White solid, yield 55%, m.p.: 100–101 °C. ^1^H-NMR (400 MHz, CDCl_3_): *δ* 7.83 (s, 1H, CH=N), 7.72–7.76 (m, 2H, Ar-H), 7.59 (d, *J* = 8.8 Hz, 2H, Ar-H), 7.16 (t, *J* = 8.8 Hz, 2H, Ar-H), 6.67 (d, *J* = 8.8 Hz, 2H, Ar-H), 6.41 (s, 1H, Isoxazole-H), 5.09 (s, 2H, CH_2_), 3.60 (s, 3H, N-CH_3_), 2.37 (s, 3H, CH_3_). ^13^C-NMR (100 MHz, CDCl_3_): *δ* 169.1, 165.0, 162.5, 161.9, 156.6, 147.1, 141.3, 138.8, 127.9, 123.8, 123.7, 117.5, 116.3, 99.9, 98.9, 86.5, 67.1, 34.3, 14.6. Anal. Calcd for C_22_H_18_FIN_4_O_3_: C, 49.64; H, 3.41; N, 10.53. Found: C, 49.52; H, 3.27; N, 10.66.

*Data for* **9i**. Yellow oil, yield 48%. ^1^H-NMR (400 MHz, CDCl_3_): *δ* 7.83 (s, 1H, CH=N), 7.68 (d, *J* = 8.4 Hz, 2H, Ar-H), 7.43 (d, *J* = 8.4 Hz, 2H, Ar-H), 6.73–6.94 (m, 3H, Ar-H), 6.46 (s, 1H, Isoxazole-H), 5.05 (s, 2H, CH_2_), 3.67 (s, 3H, N-CH_3_), 2.34 (s, 3H, CH_3_). ^13^C-NMR (100 MHz, CDCl_3_): *δ* 168.9, 162.0, 147.4, 147.2, 141.1, 136.2, 129.3, 127.1, 125.8, 123.9, 117.5, 117.4, 111.2, 110.9, 105.9, 105.4, 99.4, 99.2, 67.0, 34.3, 14.3. Anal. Calcd for C_22_H_17_F_3_N_4_O_3_: C, 59.73; H, 3.87; N, 12.66. Found: C, 59.85; H, 3.76; N, 12.51.

*Data for* **9j**. Yellow oil, yield 51%. ^1^H-NMR (400 MHz, CDCl_3_): *δ* 7.83 (s, 1H, CH=N), 7.68 (d, *J* = 8.4 Hz, 2H, Ar-H), 7.41–7.44 (m, 3H, Ar-H), 7.08–7.11 (m, 1H, Ar-H), 6.63 (d, *J* = 8.8 Hz, 1H, Ar-H), 6.44 (s, 1H, Isoxazole-H), 5.05 (s, 2H, CH_2_), 3.64 (s, 3H, CH_3_), 2.35 (s, 3H, CH_3_). ^13^C-NMR (100 MHz, CDCl_3_): *δ* 168.9, 161.9, 150.8, 147.3, 146.8, 141.0, 136.2, 130.7, 129.3, 127.9, 127.1, 125.9, 123.7, 121.9, 116.3, 99.8, 99.4, 67.1, 34.3, 14.2. Anal. Calcd for C_22_H_17_Cl_2_FN_4_O_3_: C, 55.59; H, 3.61; N, 11.79. Found: C, 55.45; H, 3.72; N, 11.94.

*Data for* **9k**. Yellow solid, yield 63%, m.p.: 113–115 °C. ^1^H-NMR (400 MHz, CDCl_3_): *δ* 7.82 (s, 1H, CH=N), 7.67 (d, *J* = 8.8 Hz, 2H, Ar-H), 7.43 (d, *J* = 8.4 Hz, 2H, Ar-H), 6.80–6.85 (m, 4H, Ar-H), 6.46 (s, 1H, Isoxazole-H), 5.11 (s, 2H, CH_2_), 3.74 (s, 3H, OCH_3_), 3.61 (s, 3H, N-CH_3_), 2.37 (s, 3H, CH_3_). ^13^C-NMR (100 MHz, CDCl_3_): *δ* 168.8, 162.1, 155.9, 150.6, 148.7, 147.0, 141.8, 136.2, 129.3, 127.1, 125.9, 116.4, 115.0, 99.6, 99.5, 67.0, 55.7, 34.2, 14.8. Anal. Calcd for C_23_H_21_ClN_4_O_4_: C, 61.00; H, 4.67; N, 12.37. Found: C, 60.84; H, 4.81; N, 12.49.

*Data for* **9l**. Yellow solid, yield 61%, m.p.: 152–154 °C. ^1^H-NMR (400 MHz, CDCl_3_): *δ* 7.85 (s, 1H, CH=N), 7.68 (d, *J* = 8.8 Hz, 2H, Ar-H), 7.43 (d, *J* = 8.4 Hz, 2H, Ar-H), 7.16 (d, *J* = 8.8 Hz, 2H, Ar-H), 6.91 (d, *J* = 9.2 Hz, 2H, Ar-H), 6.46 (s, 1H, Isoxazole-H), 5.07 (s, 2H, CH_2_), 3.62 (s, 3H, N-CH_3_), 2.37 (s, 3H, CH_3_). ^13^C-NMR (100 MHz, CDCl_3_): *δ* 168.9, 161.9, 154.9, 147.4, 147.2, 144.8, 141.3, 136.3, 129.3, 127.1, 125.8, 122.8, 116.4, 99.9, 99.4, 67.1, 34.3, 14.6. Anal. Calcd for C_23_H_18_ClF_3_N_4_O_4_: C, 54.50; H, 3.58; N, 11.05. Found: C, 54.63; H, 3.46; N, 11.16.

*Data for* **9m**. Yellow solid, yield 56%, m.p.: 96–98 °C. ^1^H-NMR (400 MHz, CDCl_3_): *δ* 7.83 (s, 1H, CH=N), 7.68 (d, *J* = 8.4 Hz, 2H, Ar-H), 7.43 (d, *J* = 8.4 Hz, 2H, Ar-H), 7.00–7.18 (m, 3H, Ar-H), 6.76–6.80 (m, 1H, Ar-H), 6.45 (s, 1H, Isoxazole-H), 5.05 (s, 2H, CH_2_), 3.67 (s, 3H, N-CH_3_), 2.36 (s, 3H, CH_3_). ^13^C-NMR (100 MHz, CDCl_3_): *δ* 168.8, 162.0, 153.2, 150.7, 147.4, 147.1, 144.1, 141.3, 136.2, 129.3, 127.1, 125.8, 124.5, 117.3, 117.1, 116.7, 99.5, 99.4, 67.0, 34.3, 14.4. Anal. Calcd for C_22_H_18_ClFN_4_O_3_: C, 59.94; H, 4.12; N, 12.71. Found: C, 59.80; H, 3.99; N, 12.79.

*Data for* **9n**. White solid, yield 52%, m.p.: 146–147 °C. ^1^H-NMR (400 MHz, CDCl_3_): *δ* 7.83 (s, 1H, CH=N), 7.67 (d, *J* = 8.4 Hz, 2H, Ar-H), 7.44 (d, *J* = 8.8 Hz, 2H, Ar-H), 6.83–7.00 (m, 4H, Ar-H), 6.44 (d, 1H, Isoxazole-H), 5.09 (s, 2H, CH_2_), 3.61 (s, 3H, N-CH_3_), 2.36 (s, 3H, CH_3_). ^13^C-NMR (100 MHz, CDCl_3_): *δ* 168.8, 162.0, 159.9, 157.5, 152.6, 147.9, 147.1, 141.5, 136.2, 129.3, 127.1, 125.8, 116.6, 116.4, 99.7, 99.4, 67.0, 34.2, 14.6. Anal. Calcd for C_22_H_18_ClFN_4_O_3_: C, 59.94; H, 4.12; N, 12.71. Found: C, 60.04; H, 4.23; N, 12.57.

*Data for* **9o**. White solid, yield 50%, m.p.: 116–118 °C. ^1^H-NMR (400 MHz, CDCl_3_): *δ* 7.85 (s, 1H, CH=N), 7.68 (d, *J* = 8.4 Hz, 2H, Ar-H), 7.44 (d, *J* = 8.8 Hz, 2H, Ar-H), 6.82–7.20 (m, 4H, Ar-H), 6.42 (s, 1H, Isoxazole-H), 5.09 (s, 2H, CH_2_), 3.61 (s, 3H, N-CH_3_), 2.37 (s, 3H, CH_3_). ^13^C-NMR (100 MHz, CDCl_3_): *δ* 168.8, 162.0, 157.1, 147.2, 141.3, 136.2, 131.1, 129.3, 127.1, 126.9, 125.8, 123.1, 118.7, 114.1, 100.0, 99.4, 67.1, 34.3, 14.5. Anal. Calcd for C_22_H_18_BrClN_4_O_3_: C, 52.66; H, 3.62; N, 11.17. Found: C, 52.79; H, 3.48; N, 11.30.

*Data for* **9p**. White solid, yield 58%, m.p.: 147–149 °C. ^1^H-NMR (400 MHz, CDCl_3_): *δ* 7.83 (s, 1H, CH=N), 7.68 (d, *J* = 8.4 Hz, 2H, Ar-H), 7.59 (d, *J* = 8.8 Hz, 2H, Ar-H), 7.45 (d, *J* = 8.4 Hz, 2H, Ar-H), 6.67 (d, *J* = 8.8 Hz, 2H, Ar-H), 6.45 (s, 1H, Isoxazole-H), 5.09 (s, 2H, CH_2_), 3.59 (s, 3H, CH_3_), 2.36 (s, 3H, CH_3_). ^13^C-NMR (100 MHz, CDCl_3_): *δ* 168.9, 161.9, 156.6, 147.2, 147.1, 141.3, 138.8, 136.2, 129.3, 127.1, 125.8, 117.5, 99.9, 99.4, 86.5, 67.1, 34.3, 14.6. Anal. Calcd for C_22_H_18_ClIN_4_O_3_: C, 48.15; H, 3.31; N, 10.21. Found: C, 48.01; H, 3.25; N, 10.33.

*Data for* **9q**. Yellow solid, yield 49%, m.p.: 80–81 °C. ^1^H-NMR (400 MHz, CDCl_3_): *δ* 7.83 (s, 1H, CH=N), 7.68 (d, *J* = 8.4 Hz, 2H, Ar-H), 7.44 (d, *J* = 8.4 Hz, 2H, Ar-H), 6.70–6.95 (m, 3H, Ar-H), 6.46 (s, 1H, Isoxazole-H), 5.06 (s, 2H, CH_2_), 3.68 (s, 3H, CH_3_), 2.34 (s, 3H, CH_3_). ^13^C-NMR (100 MHz, CDCl_3_): *δ* 167.8, 160.9, 158.5, 156.0, 152.1, 149.6, 146.4, 146.2, 140.1, 139.6, 135.2, 128.3, 126.0, 124.8, 116.5, 110.1, 104.8, 98.3, 98.2, 66.0, 33.2, 13.2. Anal. Calcd for C_22_H_17_ClF_2_N_4_O_3_: C, 57.59; H, 3.73; N, 12.21. Found: C, 57.75; H, 3.58; N, 12.08.

*Data for* **9r**. White solid, yield 61%, m.p.: 86–88 °C. ^1^H-NMR (400 MHz, CDCl_3_): *δ* 7.91–7.97 (m, 1H, Ar-H), 7.81 (s, 1H, CH=N), 6.67–7.04 (m, 7H, Ar-H and Isoxazole-H), 5.12 (s, 2H, CH_2_), 3.75 (s, 3H, OCH_3_), 3.61 (s, 3H, N-CH_3_), 2.38 (s, 3H, CH_3_). ^13^C-NMR (100 MHz, CDCl_3_): *δ* 165.1, 162.9, 162.2, 160.7, 158.3, 155.9, 150.6, 148.8, 147.0, 141.8, 128.9, 119.8, 116.4, 115.0, 112.4, 104.8, 102.9, 99.5, 66.9, 55.7, 34.2, 14.8. Anal. Calcd for C_23_H_20_F_2_N_4_O_4_: C, 60.79; H, 4.44; N, 12.33. Found: C, 60.65; H, 4.59; N, 12.45.

*Data for* **9s**. Yellow solid, yield 52%, m.p.: 106–108 °C. ^1^H-NMR (400 MHz, CDCl_3_): *δ* 7.91–7.97 (m, 1H, Ar-H), 7.83 (s, 1H, CH=N), 7.10 (d, *J* = 6.0 Hz, 2H, Ar-H), 6.63–7.03 (m, 5H, Ar-H and Isoxazole-H), 5.10 (s, 2H, CH_2_), 3.61 (s, 3H, CH_3_), 2.37 (s, 3H, CH_3_). ^13^C-NMR (100 MHz, CDCl_3_): *δ* 165.0, 162.9, 162.1, 160.8, 160.0, 158.2, 157.5, 152.6, 147.9, 147.1, 141.5, 128.9, 116.6, 116.4, 112.4, 104.8, 102.8, 99.7, 67.0, 34.2, 14.6. Anal. Calcd for C_22_H_17_F_3_N_4_O_3_: C, 59.73; H, 3.87; N, 12.66. Found: C, 59.58; H, 4.01; N, 12.78.

*Data for* **9t**. Yellow solid, yield 56%, m.p.: 108–109 °C. ^1^H-NMR (400 MHz, CDCl_3_): *δ* 7.92–7.97 (m, 1H, Ar-H), 7.84 (s, 1H, CH=N), 6.63–7.26 (m, 7H, Ar-H and Isoxazole-H), 5.10 (s, 2H, CH_2_), 3.61 (s, 3H, N-CH_3_), 2.37 (s, 3H, CH_3_). ^13^C-NMR (100 MHz, CDCl_3_): *δ* 165.0, 163.0, 162.6, 162.1, 160.7, 158.2, 155.1, 147.4, 147.2, 141.4, 129.9, 128.8, 116.6, 112.4, 112.2, 104.8, 102.8, 99.9, 67.0, 34.3, 14.5. Anal. Calcd for C_22_H_17_ClF_2_N_4_O_3_: C, 57.59; H, 3.73; N, 12.21. Found: C, 57.49; H, 3.86; N, 12.35.

*Data for* **9u**. Yellow solid, yield 60%, m.p.: 101–103 °C. ^1^H-NMR (400 MHz, CDCl_3_): *δ* 7.89 (d, *J* = 8.4 Hz, 1H, Ar-H), 7.84 (s, 1H, CH=N), 7.53 (d, *J* = 2.0 Hz, 1H, Ar-H), 6.76–7.40 (m, 6H, Ar-H and Isoxazole-H), 5.11 (s, 2H, CH_2_), 3.60 (s, 3H, N-CH_3_), 2.37 (s, 3H, CH_3_). ^13^C-NMR (100 MHz, CDCl_3_): *δ* 165.2, 161.8, 155.7, 147.3, 147.1, 141.4, 136.2, 132.9, 132.2, 130.6, 130.1, 127.7, 124.7, 117.0, 116.2, 104.3, 99.8, 67.0, 34.3, 14.6. Anal. Calcd for C_22_H_17_BrF_2_N_4_O_3_: C, 52.50; H, 3.40; N, 11.13. Found: C, 52.66; H, 3.27; N, 11.27.

*Data for* **9v**. Yellow solid, yield 58%, m.p.: 104–106 °C. ^1^H-NMR (400 MHz, CDCl_3_): *δ* 7.89 (d, *J* = 8.8 Hz, 1H, Ar-H), 7.83 (s, 1H, CH=N), 7.58 (d, *J* = 8.4 Hz, 2H, Ar-H), 6.65–7.54 (m, 5H, Ar-H and Isoxazole-H), 5.11 (s, 2H, CH_2_), 3.60 (s, 3H, CH_3_), 2.38 (s, 3H, CH_3_). ^13^C-NMR (100 MHz, CDCl_3_): *δ* 165.2, 161.8, 156.5, 147.3, 147.1, 141.4, 138.8, 136.3, 132.3, 130.7, 130.1, 127.7, 124.7, 117.5, 104.3, 99.9, 86.6, 67.0, 34.3, 14.6. Anal. Calcd for C_22_H_17_F_2_IN_4_O_3_: C, 48.02; H, 3.11; N, 10.18. Found: C, 48.13; H, 3.01; N, 10.33.

#### 3.1.7. General Procedure for the Preparation of **10**

To a mixture of intermediate **8** (10 mmol), 4-hydroxybenzaldehyde (12 mmol) in acetonitrile (60 mL) at room temperature, was added cesium carbonate (13 mmol). The resulting mixture was heated to reflux for 5 h. After being cooled to room temperature, the mixture was poured into water (100 mL), and the solid precipitate was filtered, washed with water, and dried to afford corresponding compound **10,** with yields ranging from 71% to 77%, which could be used for the next reaction without further purification.

#### 3.1.8. General Procedure for the Preparation of **11**

To a well stirred cold (0 °C) solution of intermediate **10** (4 mmol) and THF (60 mL), was added LiAlH_4_ (6 mmol) in three portions and the reaction mixture was stirred at 0 °C for 30 min. To the above solution, was added ice water (40 mL). After the solid precipitate was filtered, the filtrate was extracted with ethyl acetate (3 × 30 mL). The combined extracts were dried over anhydrous sodium sulfate, filtered, and concentrated to give compound **11** with yields ranging from 68% to 72%, which could be used for the next reaction without further purification.

#### 3.1.9. General Procedure for the Preparation of **12**

To a well stirred cold (0 °C) solution of compound **11** (4 mmol) in CH_2_Cl_2_ (50 mL), was added dropwise a mixture of thionyl chloride (8 mmol) in CH_2_Cl_2_ (10 mL). Then, several drops of DMF was added thereto. The resulting mixture was stirred at 0 °C for 6–8 h. To the above solution, was added ice water (50 mL), and the pH value of the mixture was adjusted to 6 by saturated sodium bicarbonate solution. The organic layer was dried over anhydrous sodium sulfate, filtered, and concentrated to afford the corresponding compound **12**, with yields ranging from 62% to 65%, which could be used for the following transformations without further purification.

#### 3.1.10. General Procedure for the Preparation of **13a**–**13f**

To a mixture of intermediate **12** (4 mmol), compound **3** (5 mmol), and potassium carbonate (10 mmol) in acetonitrile (30 mL) at room temperature. The resulting mixture was heated to reflux for 10–15 h. The reaction mixture was allowed to cool at room temperature and filtered. The solvent was evaporated under reduced pressure, and the residue was admixed with water (40 mL) and extracted with ethyl acetate (3 × 30 mL). The combined organic layer was washed with water (3 × 20 mL), dried over anhydrous sodium sulfate, filtered, and concentrated. The residue was purified by silica gel column chromatography using a mixture of petroleum ether and ethyl acetate as an eluent to produce the title compounds **13a**–**13f**, with yields ranging from 46% to 56%. Pyrazole oxime derivatives **13a**–**13f** were novel and the physical and spectral data for these compounds are listed below. ^1^H-NMR and ^13^C-NMR spectra are provided in the Supplementary Materials.

*Data for* **13a**. White solid, yield 50%, m.p.: 77–79 °C. ^1^H-NMR (400 MHz, CDCl_3_): *δ* 7.75–7.78 (m, 3H, Ar-H and CH=N), 7.07–7.26 (m, 6H, Ar-H), 6.94 (d, *J* = 8.0 Hz, 2H, Ar-H), 6.76 (d, *J* = 8.0 Hz, 2H, Ar-H), 6.58 (s, 1H, Isoxazole-H), 5.18 (s, 2H, CH_2_), 4.94 (s, 2H, CH_2_), 3.58 (s, 3H, N-CH_3_), 2.37 (s, 3H, CH_3_), 2.30 (s, 3H, CH_3_). ^13^C-NMR (100 MHz, CDCl_3_): *δ* 169.6, 165.1, 162.6, 161.5, 157.8, 154.7, 148.1, 146.8, 140.7, 133.1, 130.8, 130.4, 128.0, 127.9, 123.6, 116.4, 116.1, 115.1, 114.6, 100.2, 98.6, 75.6, 61.8, 34.2, 20.6, 14.9. Anal. Calcd for C_30_H_27_FN_4_O_4_: C, 68.43; H, 5.17; N, 10.64. Found: C, 68.59; H, 5.05; N, 10.77.

*Data for* **13b**. White solid, yield 48%, m.p.: 99–101 °C. ^1^H-NMR (400 MHz, CDCl_3_): *δ* 7.75–7.78 (m, 3H, Ar-H and CH=N), 7.13–7.24 (m, 4H, Ar-H), 6.82–7.00 (m, 6H, Ar-H), 6.59 (s, 1H, Isoxazole-H), 5.18 (s, 2H, CH_2_), 4.92 (s, 2H, CH_2_), 3.60 (s, 3H, N-CH_3_), 2.35 (s, 3H, CH_3_). ^13^C-NMR (100 MHz, CDCl_3_): *δ* 169.6, 165.1, 162.6, 161.5, 160.0, 157.8, 157.5, 152.7, 152.6, 147.7, 147.0, 140.4, 130.7, 130.3, 128.0, 127.9, 123.6, 116.6, 116.5, 116.4, 116.1, 114.6, 100.2, 98.6, 75.7, 61.8, 34.2, 14.6. Anal. Calcd for C_29_H_24_F_2_N_4_O_4_: C, 65.65; H, 4.56; N, 10.56. Found: C, 65.78; H, 4.42; N, 10.65.

*Data for* **13c**. White solid, yield 46%, m.p.: 91–93 °C. ^1^H-NMR (400 MHz, CDCl_3_): *δ* 7.75–7.78 (m, 3H, Ar-H and CH=N), 7.40 (d, *J* = 8.0 Hz, 2H, Ar-H), 7.13–7.22 (m, 4H, Ar-H), 6.94 (d, *J* = 8.0 Hz, 2H, Ar-H), 6.76 (d, *J* = 8.0 Hz, 2H, Ar-H), 6.59 (s, 1H, Isoxazole-H), 5.18 (s, 2H, CH_2_), 4.91 (s, 2H, CH_2_), 3.59 (s, 3H, N-CH_3_), 2.34 (s, 3H, CH_3_). ^13^C-NMR (100 MHz, CDCl_3_): *δ* 169.6, 165.1, 162.6, 161.5, 157.8, 155.8, 147.0, 140.3, 132.9, 130.7, 130.3, 128.0, 127.9, 123.6, 117.1, 116.4, 116.1, 114.6, 100.3, 98.6, 75.7, 61.8, 34.2, 14.5. Anal. Calcd for C_29_H_24_BrFN_4_O_4_: C, 58.89; H, 4.09; N, 9.47. Found: C, 58.74; H, 4.20; N, 9.61.

*Data for* **13d**. White solid, yield 53%, m.p.: 97–99 °C. ^1^H-NMR (400 MHz, CDCl_3_): *δ* 7.75–7.78 (m, 3H, Ar-H and CH=N), 7.59 (d, *J* = 8.0 Hz, 2H, Ar-H), 7.13–7.22 (m, 4H, Ar-H), 6.95 (d, *J* = 8.0 Hz, 2H, Ar-H), 6.65 (d, *J* = 8.0 Hz, 2H, Ar-H), 6.59 (s, 1H, Isoxazole-H), 5.19 (s, 2H, CH_2_), 4.91 (s, 2H, CH_2_), 3.58 (s, 3H, N-CH_3_), 2.34 (s, 3H, CH_3_). ^13^C-NMR (100 MHz, CDCl_3_): *δ* 169.5, 165.1, 162.6, 161.5, 157.8, 156.6, 147.0, 146.9, 140.2, 138.8, 130.7, 130.3, 128.0, 127.9, 123.6, 117.5, 116.4, 116.1, 114.6, 100.3, 98.6, 86.4, 75.7, 61.8, 34.2, 14.5. Anal. Calcd for C_29_H_24_FIN_4_O_4_: C, 54.56; H, 3.79; N, 8.78. Found: C, 54.69; H, 3.90; N, 8.64.

*Data for* **13e**. White solid, yield 51%, m.p.: 88–90 °C. ^1^H-NMR (400 MHz, CDCl_3_): *δ* 7.77 (s, 1H, CH=N), 7.70 (d, *J* = 8.0 Hz, 2H, Ar-H), 7.39–7.44 (m, 4H, Ar-H), 7.21 (d, *J* = 8.0 Hz, 2H, Ar-H), 6.94 (d, *J* = 8.0 Hz, 2H, Ar-H), 6.76 (d, *J* = 8.0 Hz, 2H, Ar-H), 6.63 (s, 1H, Isoxazole-H), 5.19 (s, 2H, CH_2_), 4.91 (s, 2H, CH_2_), 3.59 (s, 3H, N-CH_3_), 2.34 (s, 3H, CH_3_). ^13^C-NMR (100 MHz, CDCl_3_): *δ* 169.4, 161.6, 157.8, 155.8, 147.0, 140.2, 136.4, 133.3, 132.9, 130.7, 130.3, 129.3, 127.1, 125.7, 119.4, 117.1, 116.1, 114.6, 100.3, 99.1, 75.7, 61.7, 34.2, 14.5. Anal. Calcd for C_29_H_24_BrClN_4_O_4_: C, 57.30; H, 3.98; N, 9.22. Found: C, 57.14; H, 3.85; N, 9.36.

*Data for* **13f**. White solid, yield 56%, m.p.: 83–85 °C. ^1^H-NMR (400 MHz, CDCl_3_): *δ* 7.76 (s, 1H, CH=N), 7.71 (d, *J* = 8.0 Hz, 2H, Ar-H), 7.59 (d, *J* = 8.0 Hz, 2H, Ar-H), 7.44 (d, *J* = 8.0 Hz, 2H, Ar-H), 7.21 (d, *J* = 8.0 Hz, 2H, Ar-H), 6.63–6.96 (m, 5H, Ar-H and Isoxazole-H), 5.19 (s, 2H, CH_2_), 4.91 (s, 2H, CH_2_), 3.59 (s, 3H, N-CH_3_), 2.34 (s, 3H, CH_3_). ^13^C-NMR (100 MHz, CDCl_3_): *δ* 169.4, 161.6, 157.8, 156.6, 147.0, 146.9, 140.2, 139.2, 138.8, 136.4, 130.7, 130.4, 129.3, 127.1, 119.6, 117.6, 114.6, 100.4, 99.2, 86.4, 75.7, 61.8, 34.2, 14.5. Anal. Calcd for C_29_H_24_ClIN_4_O_4_: C, 53.19; H, 3.69; N, 8.56. Found: C, 53.33; H, 3.54; N, 8.47.

### 3.2. Biological Tests

#### 3.2.1. Bioassay Methods

All of the bioassays were performed on representative test organisms reared in the laboratory. The bioassay was repeated in triplicate. Acaricidal and insecticidal assessments were made on a dead/alive basis, and mortality rates were corrected using Abbott’s formula.

#### 3.2.2. Insecticidal Activities against *Mythimna separata*

The larvicidal activities of the title compounds against *Mythimna separata* were tested by foliar application [29]. Corn leaves were dipped into the obtained solutions for 2–3 s. After air-drying, the soaked leaves were put into a culture dish with a piece of filter paper, followed by inoculation of 10 third-instar *M. separata* larvae per dish. Covered with gauze and kept in observation room for normal cultivation at 24 °C–27 °C. Mortality was assessed 48 h after treatment. The individuals who did not respond to the touch of writing brush were recognized as dead. Each test was run three times and the results were averaged. Pyridalyl, as the control compound, was tested under the same conditions.

#### 3.2.3. Acaricidal Activities against *Tetranychus cinnabarinus*, and Insecticidal Activities against *Aphis medicaginis* and *Nilaparvata lugens*

The acaricidal activities against *Tetranychus cinnabarinus*, and insecticidal activities against *Aphis medicaginis* and *Nilaparvata lugens* of the title compounds were tested by the spray method [30]. Under the Potter spray tower, horsebean leaves, inoculated with *T. cinnabarinus* were separately treated with solutions of tested compounds. After that, the resultant horsebean leaves were kept in an observation room for normal cultivation at 24 °C–27 °C. Mortality was assessed 48 h after treatment. Each test was run three times and results were averaged. Fenpyroximate was used as the control. Activities against *A. medicaginis* were evaluated by the similar procedure except that the culture temperature was reduced to 20 °C–22 °C. Inhibitions of *N. lugens* were tested on the rice seedlings, which was inoculated with *N. lugens* first. After that, the resultant rice seedlings were kept in an observation room for normal cultivation at 24 °C–27 °C. Mortality was assessed 48 h after treatment. All of the tests were run with three duplicates and the results were averaged. Chlorantraniliprole and Abamectin were used as the positive controls, respectively.

## 4. Conclusions

In summary, a series of novel pyrazole oxime compounds containing isoxazole moiety were prepared and evaluated for their acaricidal activity against *T. cinnabarinus*, and insecticidal activities against *A. medicaginis*, *M. separata* and *N. lugens*. Bioassays results revealed that some title compounds exhibited potent acaricidal and insecticidal activities. Among these compounds, compound **9e** showed 80.46% acaricidal activity against *T. cinnabarinus* at 500 μg/mL, compounds **9c**, **9h**, **9u**, and **9v** had 100.00%, 90.56%, 90.78%, and 90.62% insecticidal activity against *A. medicaginis* at the concentration of 20 μg/mL, respectively, compounds **9k** and **9u** possessed 70.86% and 100.00% insecticidal activity against *M. separata* at the dosage of 20 μg/mL, respectively, and insecticidal activity against *N. lugens* of compounds **9e** and **9g** were 75.23% and 75.76% at 100 μg/mL. Further analogue synthesis and structural optimization are well under way.

## Figures and Tables

**Table 1:** Acaricidal and insecticidal activities of target compounds **9a**–**9v** and **13a**–**13f** (mortality, %).

Compd.	*Tetranychus cinnabarinus*		*Aphis medicaginis*		
	500 μg/mL	100 μg/mL	500 μg/mL	100 μg/mL	20 μg/mL
**9a**	0	— ^b^	90.52 ± 0.72	40.54 ± 1.22	0
**9b**	0	—	100.00 ± 0.00	100.00 ± 0.00	40.89 ± 0.57
**9c**	0	—	100.00 ± 0.00	100.00 ± 0.00	100.00 ± 0.00
**9d**	0	—	100.00 ± 0.00	100.00 ± 0.00	60.36 ± 1.08
**9e**	80.46 ± 0.65 ^a^	50.49 ± 1.78	100.00 ± 0.00	90.66 ± 0.53	50.67 ± 1.36
**9f**	0	—	50.33 ± 1.59	0	—
**9g**	0	—	100.00 ± 0.00	100.00 ± 0.00	80.73 ± 0.71
**9h**	0	—	100.00 ± 0.00	100.00 ± 0.00	90.56 ± 0.82
**9i**	0	—	100.00 ± 0.00	100.00 ± 0.00	85.83 ± 0.69
**9j**	0	—	100.00 ± 0.00	100.00 ± 0.00	80.28 ± 1.21
**9k**	0	—	100.00 ± 0.00	0	—
**9l**	0	—	0	—	—
**9m**	0	—	50.72 ± 1.37	0	—
**9n**	0	—	0	—	—
**9o**	0	—	0	—	—
**9p**	0	—	0	—	—
**9q**	50.38 ± 1.23	0	100.00 ± 0.00	0	—
**9r**	0	—	100.00 ± 0.00	70.89 ± 1.25	0
**9s**	0	—	100.00 ± 0.00	40.57 ± 0.68	—
**9t**	0	—	100.00 ± 0.00	0	—
**9u**	0	—	100.00 ± 0.00	100.00 ± 0.00	90.78 ± 1.35
**9v**	0	—	100.00 ± 0.00	100.00 ± 0.00	90.62 ± 0.96
**13a**	0	—	0	—	—
**13b**	0	—	0	—	—
**13c**	20.26 ± 1.61	—	100.00 ± 0.00	20.92 ± 0.52	—
**13d**	0	—	0	—	—
**13e**	0	—	0	—	—
**13f**	0	—	100.00 ± 0.00	30.81 ± 1.47	—
Fenpyroximate	100.00 ± 0.00	100.00 ± 0.00	—	—	—
Chlorantraniliprole	—	—	100.00 ± 0.00	100.00 ± 0.00	100.00 ± 0.00

^a^ Each value represents the mean ± standard error of three replications. ^b^ “—” refers to “not tested”.

**Table 2:** Insecticidal activities of title compounds **9a**–**9v** and **13a**–**13f** (mortality, %).

Compd.	*Mythimna separata*			*Nilaparvata lugens*		
	500 μg/mL	100 μg/mL	20 μg/mL	500 μg/mL	100 μg/mL	20 μg/mL
**9a**	0	— ^b^	—	85.54 ± 1.32	0	—
**9b**	90.37 ± 0.85 ^a^	0	—	0	—	—
**9c**	100.00 ± 0.00	100.00 ± 0.00	40.57 ± 0.76	100.00 ± 0.00	0	—
**9d**	60.29 ± 1.03	0	—	100.00 ± 0.00	0	—
**9e**	50.55 ± 0.92	0	—	90.26 ± 1.45	75.23 ± 0.69	30.87 ± 1.43
**9f**	80.76 ± 0.65	0	—	50.32 ± 1.21	0	—
**9g**	80.32 ± 0.82	50.43 ± 0.73	0	80.79 ± 0.87	75.76 ± 0.75	0
**9h**	100.00 ± 0.00	40.56 ± 0.47	—	95.23 ± 0.65	50.43 ± 1.58	0
**9i**	100.00 ± 0.00	30.19 ± 1.58	—	100.00 ± 0.00	0	—
**9j**	90.74 ± 0.57	0	—	90.86 ± 0.71	0	—
**9k**	100.00 ± 0.00	100.00 ± 0.00	70.86 ± 0.63	100.00 ± 0.00	0	—
**9l**	0	—	—	50.43 ± 0.54	0	—
**9m**	0	—	—	80.31 ± 1.29	0	—
**9n**	0	—	—	0	—	—
**9o**	100.00 ± 0.00	0	—	0	—	—
**9p**	100.00 ± 0.00	60.58 ± 1.21	0	0	—	—
**9q**	100.00 ± 0.00	0	—	70.32 ± 0.86	0	—
**9r**	100.00 ± 0.00	30.48 ± 0.65	—	100.00 ± 0.00	40.87 ± 0.73	—
**9s**	100.00 ± 0.00	0	—	100.00 ± 0.00	0	—
**9t**	100.00 ± 0.00	70.18 ± 1.43	30.27 ± 1.22	100.00 ± 0.00	30.75 ± 0.82	—
**9u**	100.00 ± 0.00	100.00 ± 0.00	100.00 ± 0.00	0	—	—
**9v**	100.00 ± 0.00	50.21 ± 1.56	0	0	—	—
**13a**	100.00 ± 0.00	40.43 ± 1.81	—	0	—	—
**13b**	100.00 ± 0.00	30.61 ± 0.63	—	0	—	—
**13c**	100.00 ± 0.00	0	—	20.68 ± 1.21	—	—
**13d**	70.91 ± 0.89	0	—	100.00 ± 0.00	20.65 ± 0.53	—
**13e**	100.00 ± 0.00	0	—	30.21 ± 1.12	—	—
**13f**	100.00 ± 0.00	0	—	20.57 ± 0.43	—	—
Pyridalyl	100.00 ± 0.00	100.00 ± 0.00	100.00 ± 0.00	—	—	—
Abamectin	—	—	—	100.00 ± 0.00	100.00 ± 0.00	100.00 ± 0.00

^a^ Each value represents the mean ± standard error of three replications. ^b^ “—” refers to “not tested”.

## Supplementary Materials

The following are available online.

## Notes

**Sample Availability:** Samples of the compounds **9a**–**9v** and **13a**–**13f** are available from the authors.
