# Supplementary material for: Design, Synthesis and Bioactivities of Novel Isoxazole-Containing Pyrazole Oxime Derivatives
Source: Molecules. 2017 Nov 27;22(12):2000. doi: 10.3390/molecules22122000 (PMC6149770; doi:10.3390/molecules22122000)
Supplement: Supplementary file 1 [file molecules-22-02000-s001.pdf]

molecules

MDPI

Supplementary Materials

The ^{1}H-NMR and ^{13}C-NMR spectra of pyrazole oxime derivatives **9a–9v** and **13a–13f** were listed below:

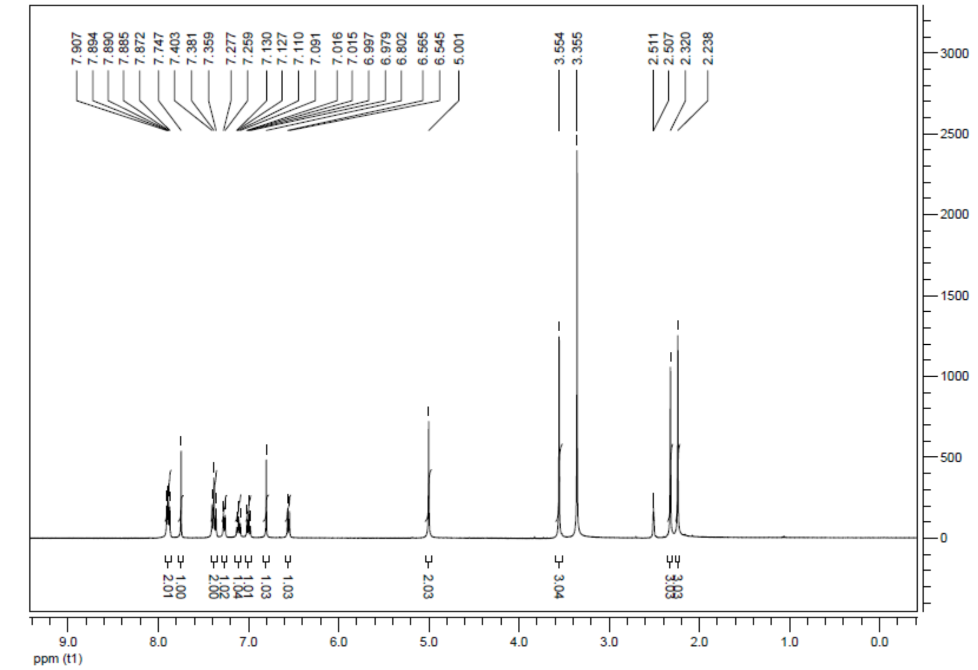

Figure S1. ^{1}H-NMR of compound **9a** (400 MHz, d_6-DMSO).

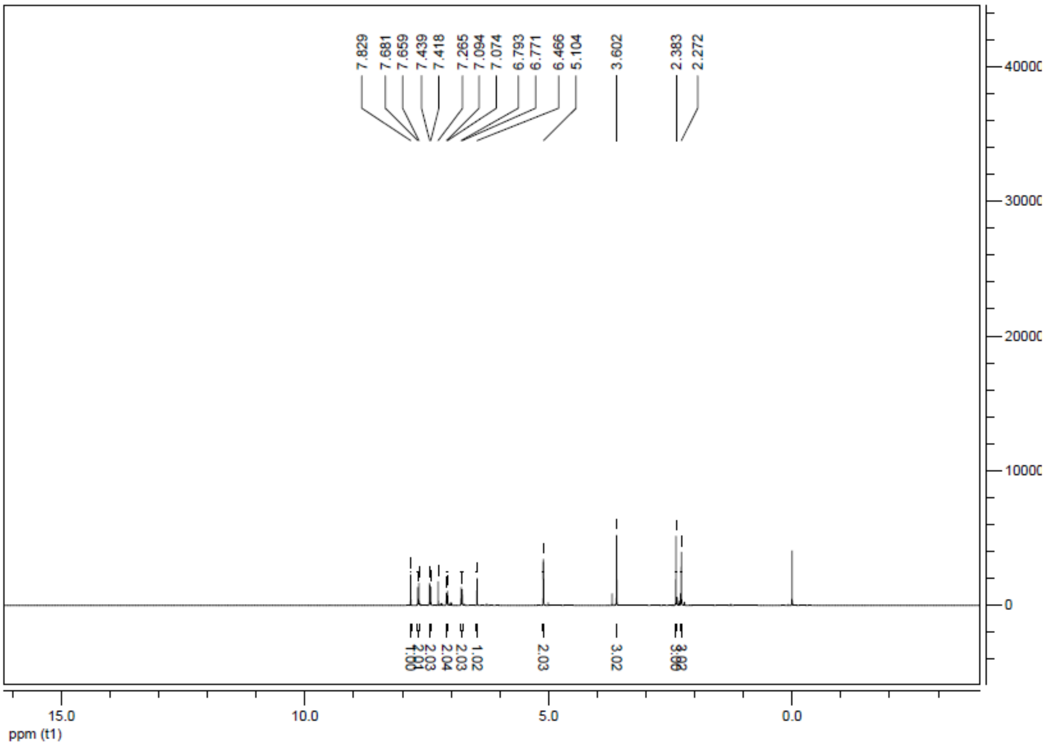

Figure S2. ^{1}H-NMR of compound **9b** (400 MHz, $CDCl_3$).

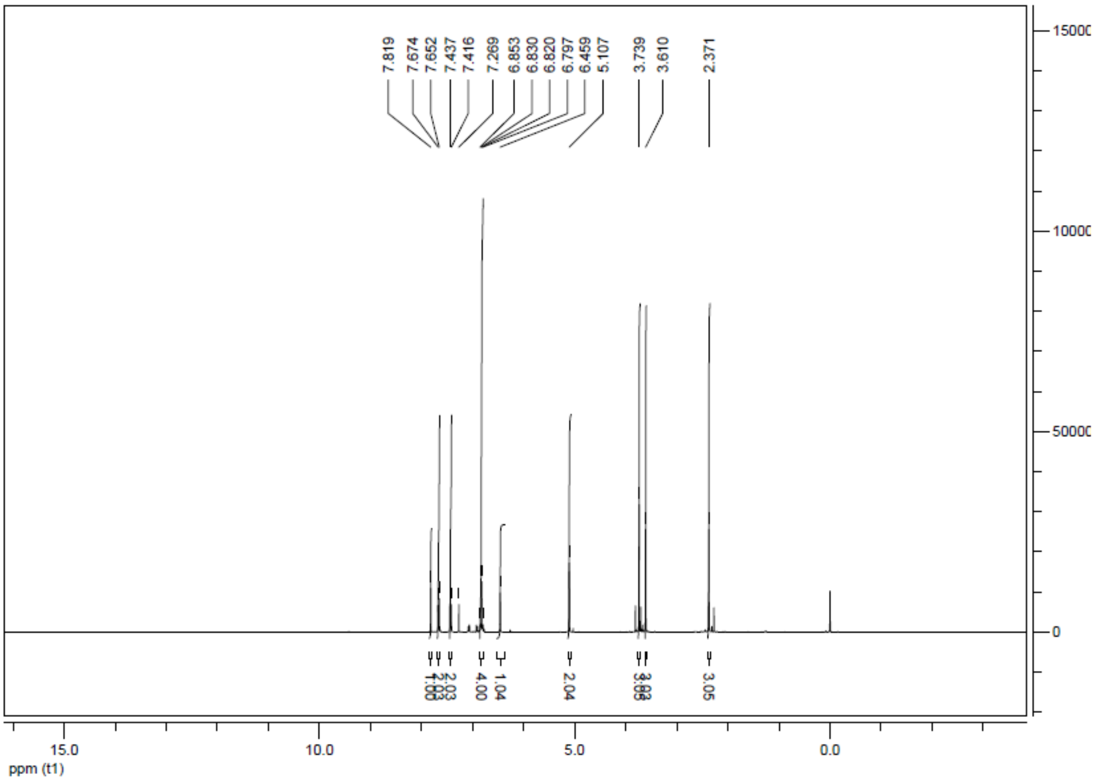

Figure S3. ^{1}H-NMR of compound **9c** (400 MHz, $CDCl_3$).

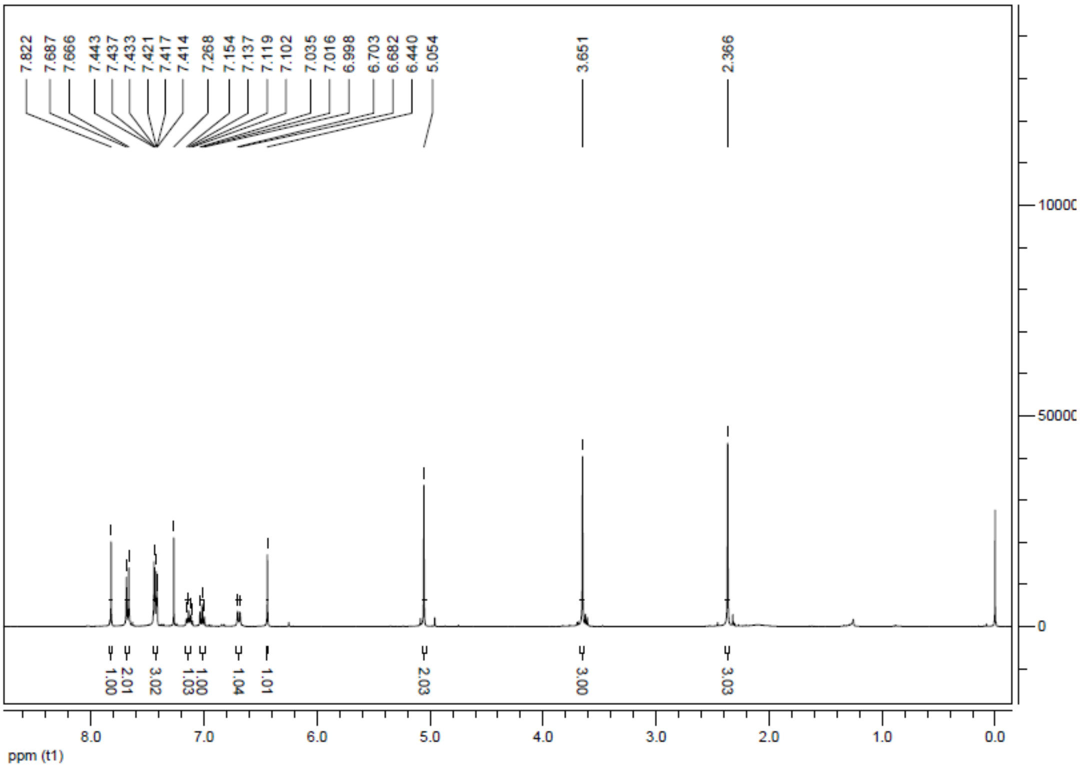

Figure S4. ^{1}H-NMR of compound **9d** (400 MHz, $CDCl_3$).

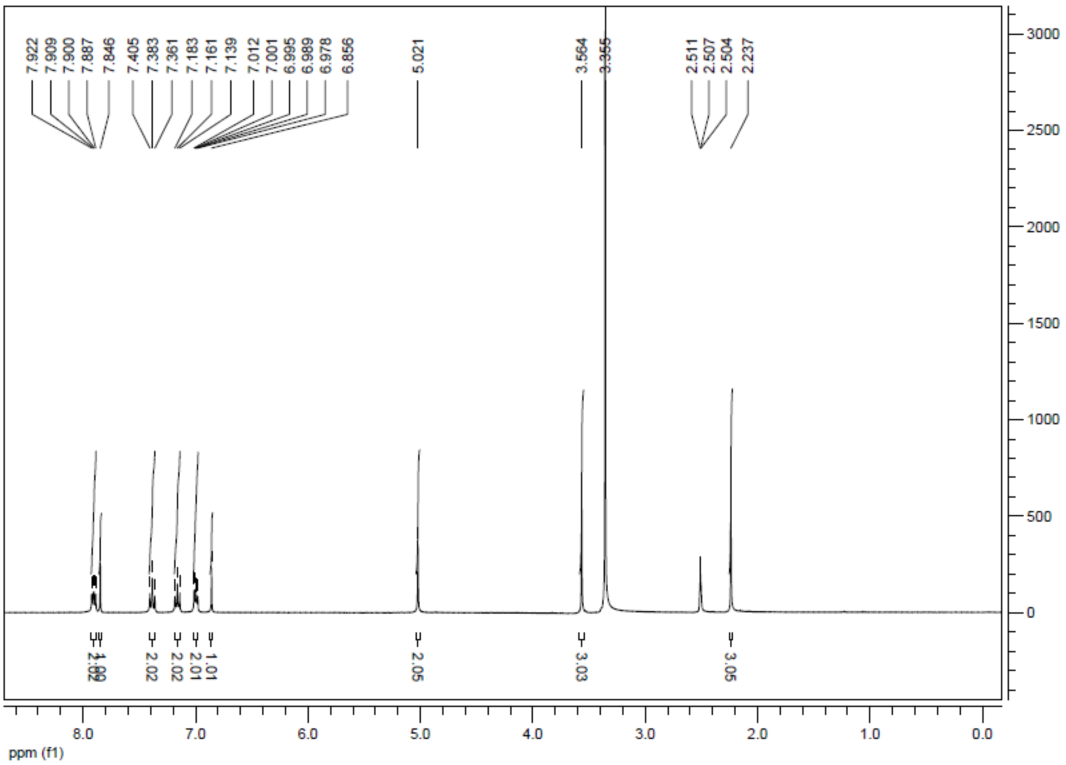

Figure S5. ^{1}H-NMR of compound **9e** (400 MHz, d_6-DMSO).

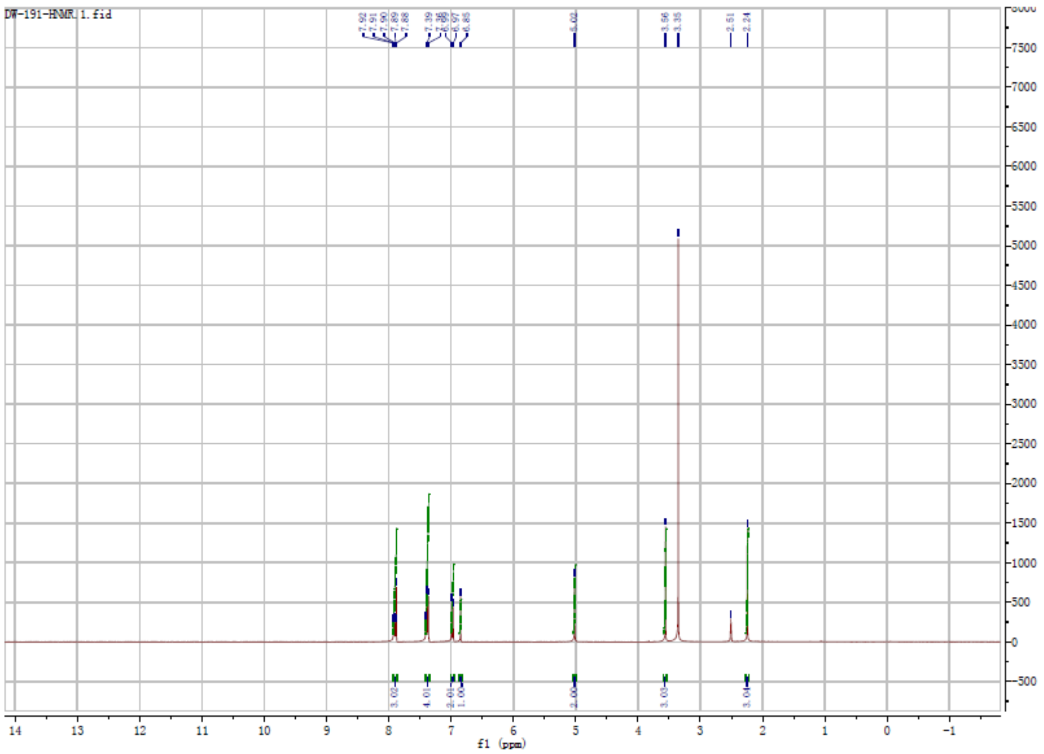

Figure S6. ^{1}H-NMR of compound **9f** (400 MHz, d_6-DMSO).

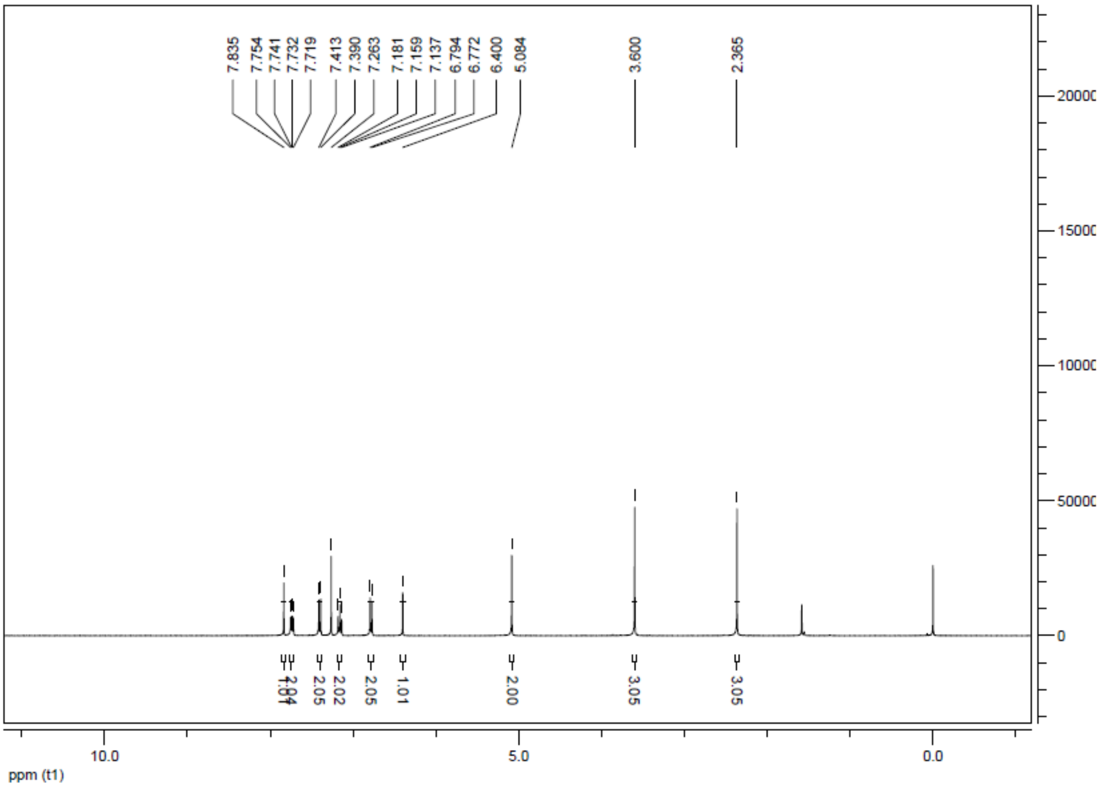

Figure S7. ^{1}H-NMR of compound **9g** (400 MHz, $CDCl_3$).

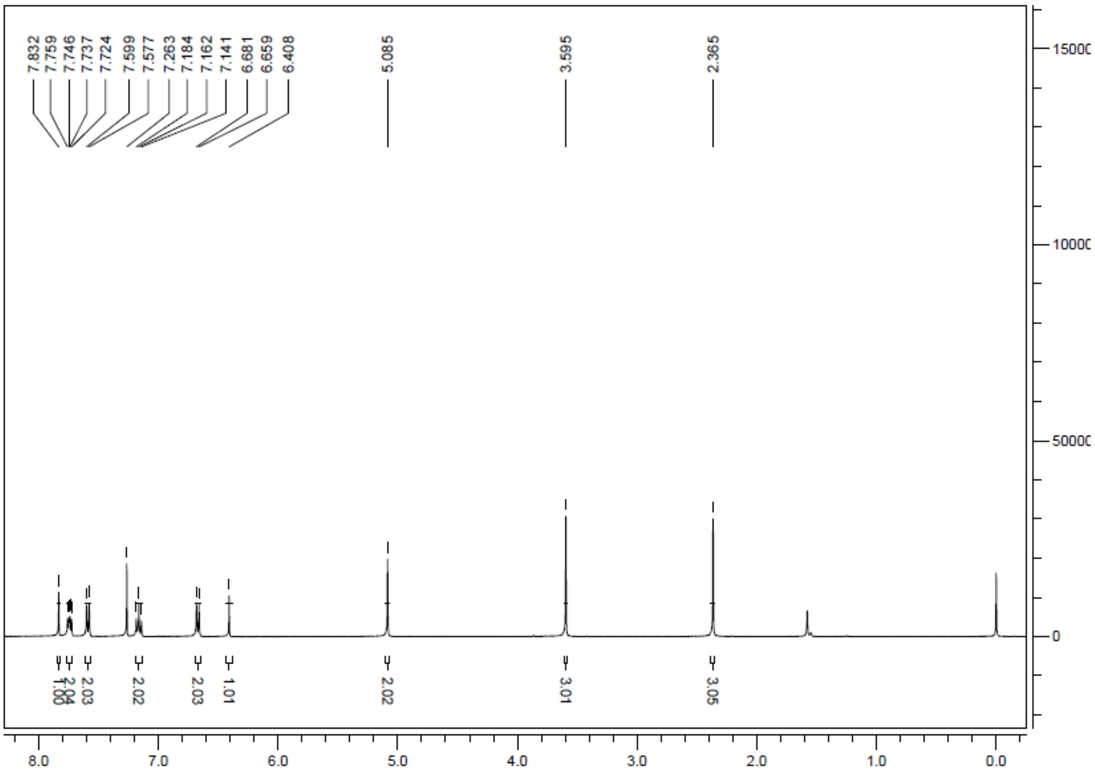

Figure S8. ^{1}H-NMR of compound **9h** (400 MHz, $CDCl_3$).

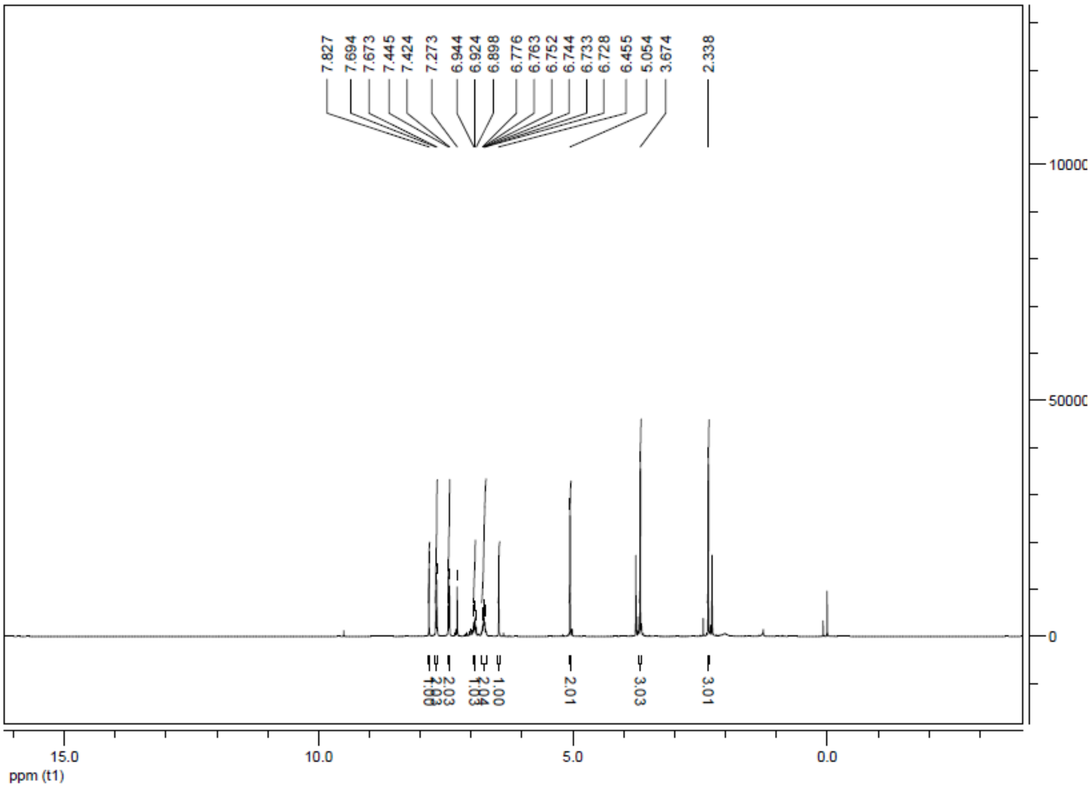

Figure S9. ^{1}H-NMR of compound **9i** (400 MHz, $CDCl_3$).

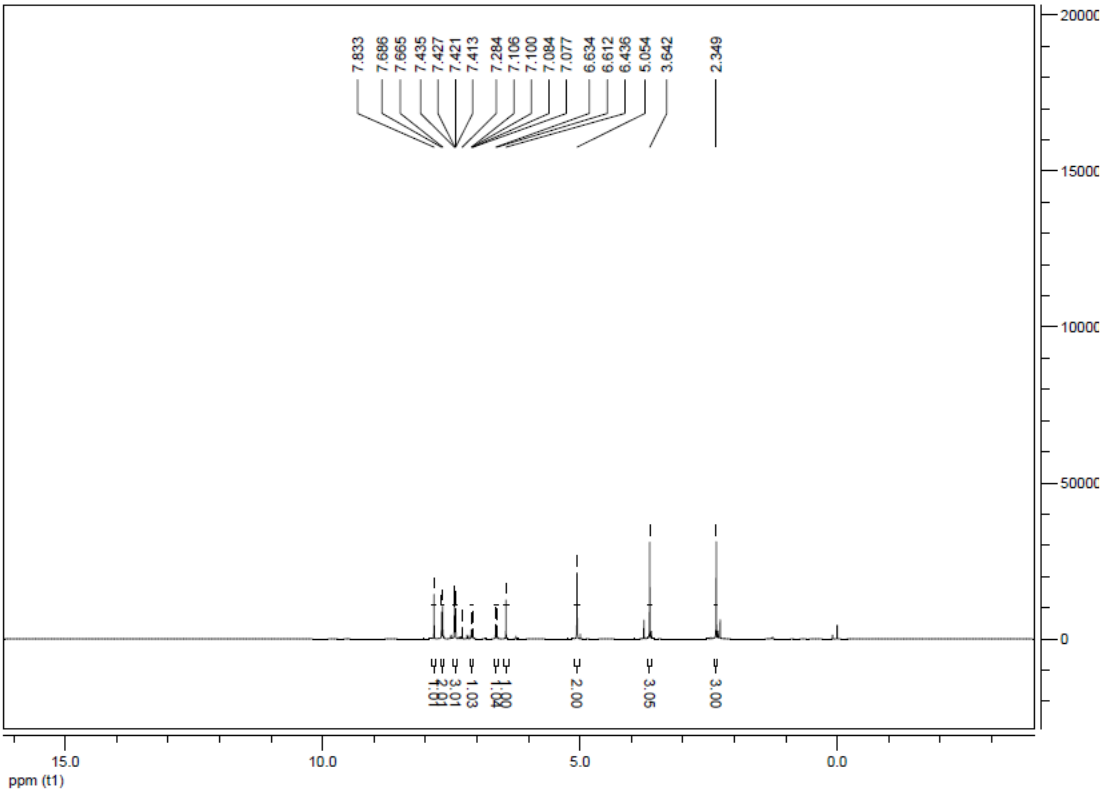

Figure S10. ^{1}H-NMR of compound **9j** (400 MHz, $CDCl_3$).

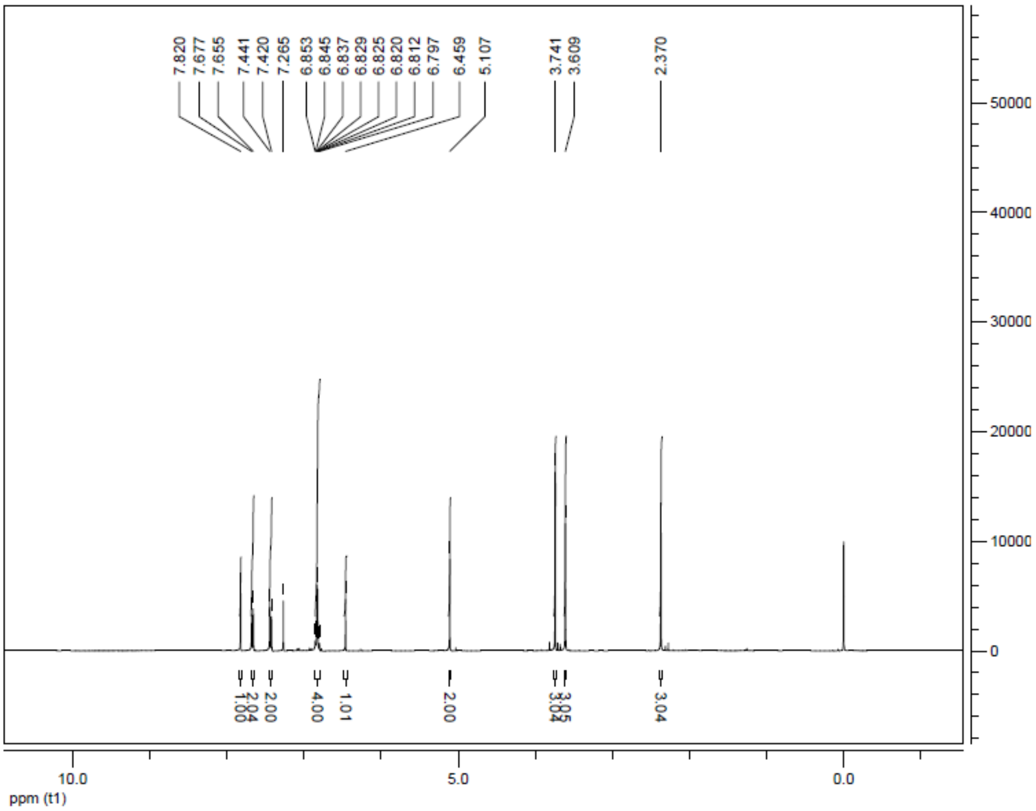

Figure S11. ^{1}H-NMR of compound **9k** (400 MHz, $CDCl_3$).

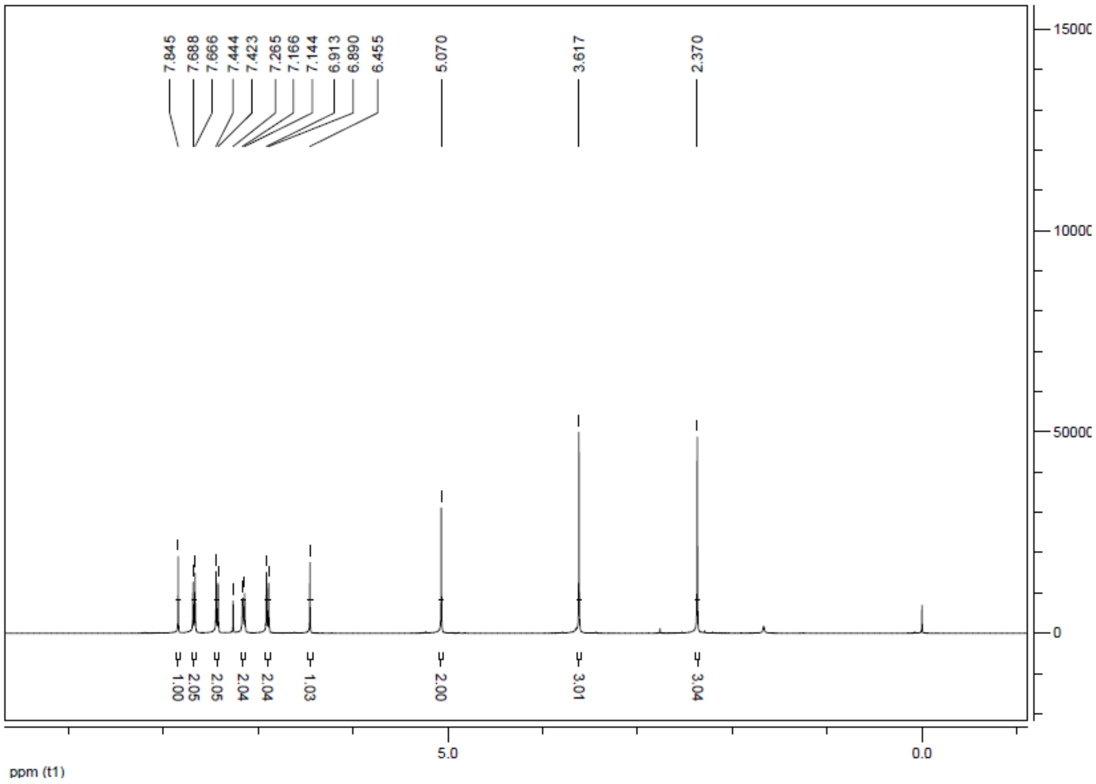

Figure S12. ^{1}H-NMR of compound **9l** (400 MHz, $CDCl_3$).

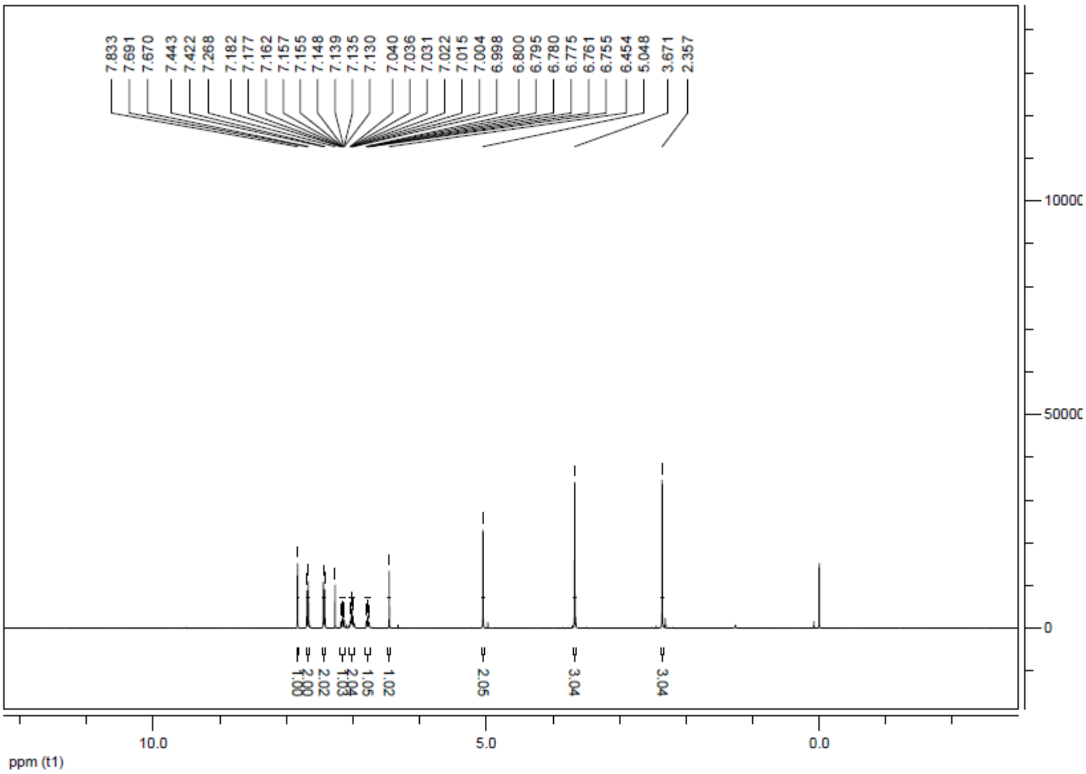

Figure S13. ^{1}H-NMR of compound **9m** (400 MHz, $CDCl_3$).

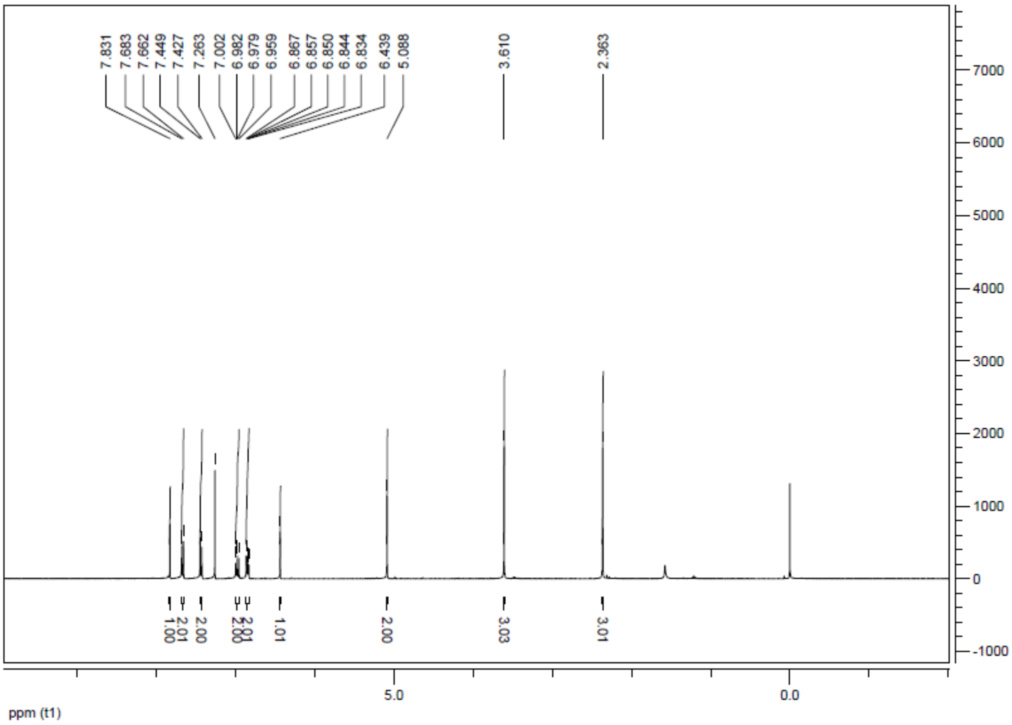

Figure S14. ^{1}H-NMR of compound **9n** (400 MHz, $CDCl_3$).

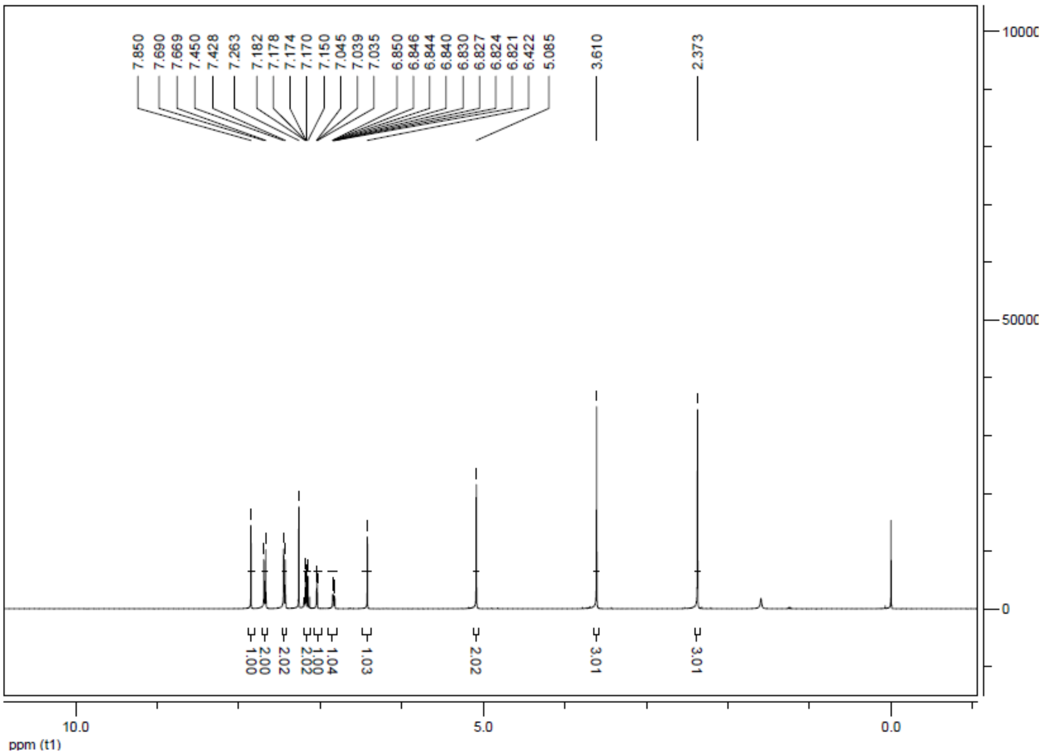

Figure S15. ^{1}H-NMR of compound **9o** (400 MHz, $CDCl_3$).

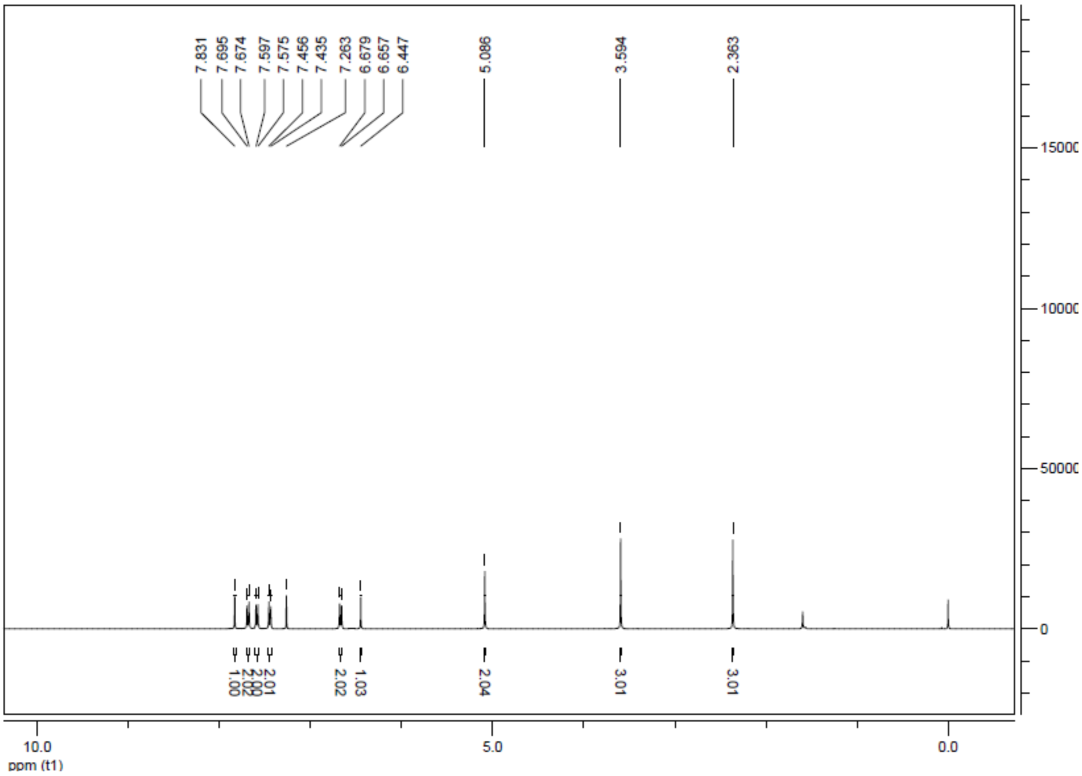

Figure S16. ^{1}H-NMR of compound **9p** (400 MHz, $CDCl_3$).

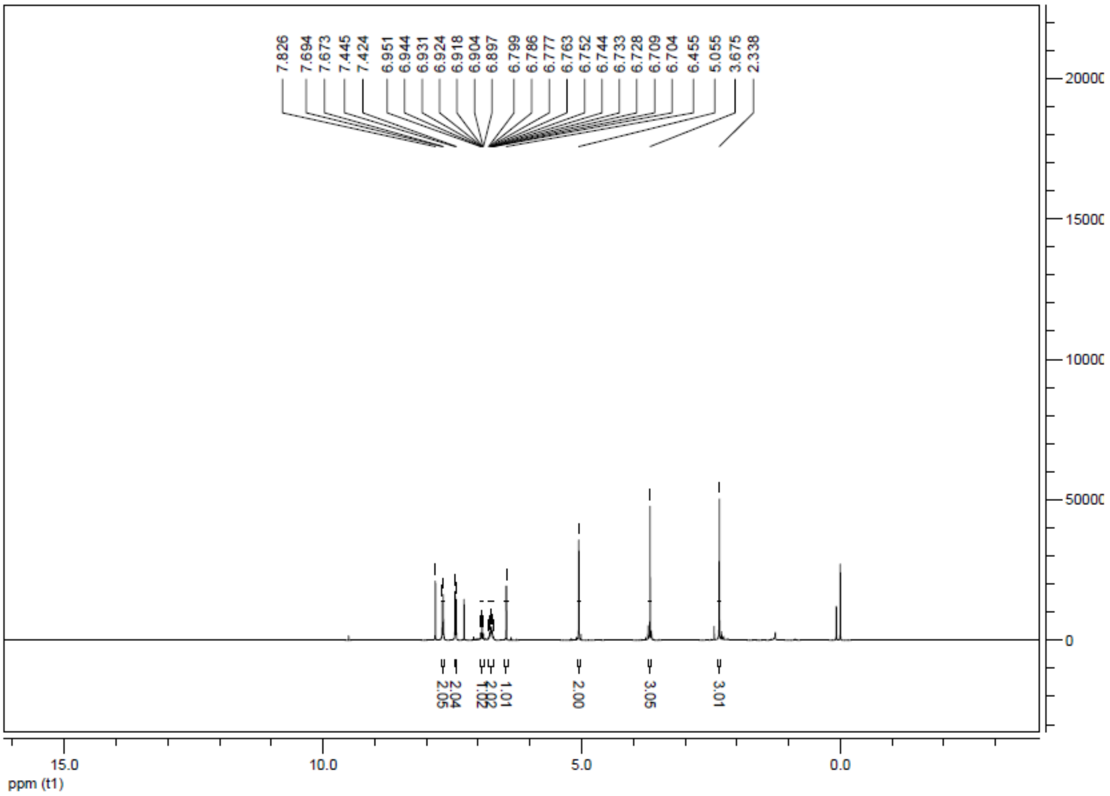

Figure S17. ^{1}H-NMR of compound **9q** (400 MHz, $CDCl_3$).

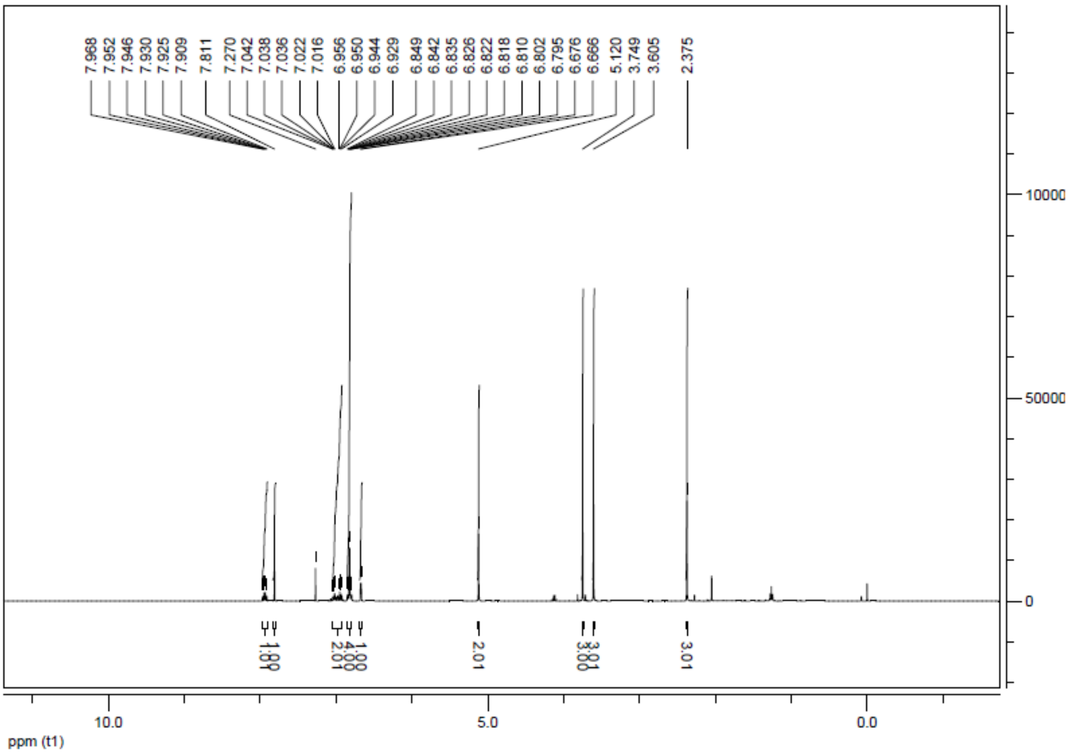

Figure S18. ^{1}H-NMR of compound **9r** (400 MHz, $CDCl_3$).

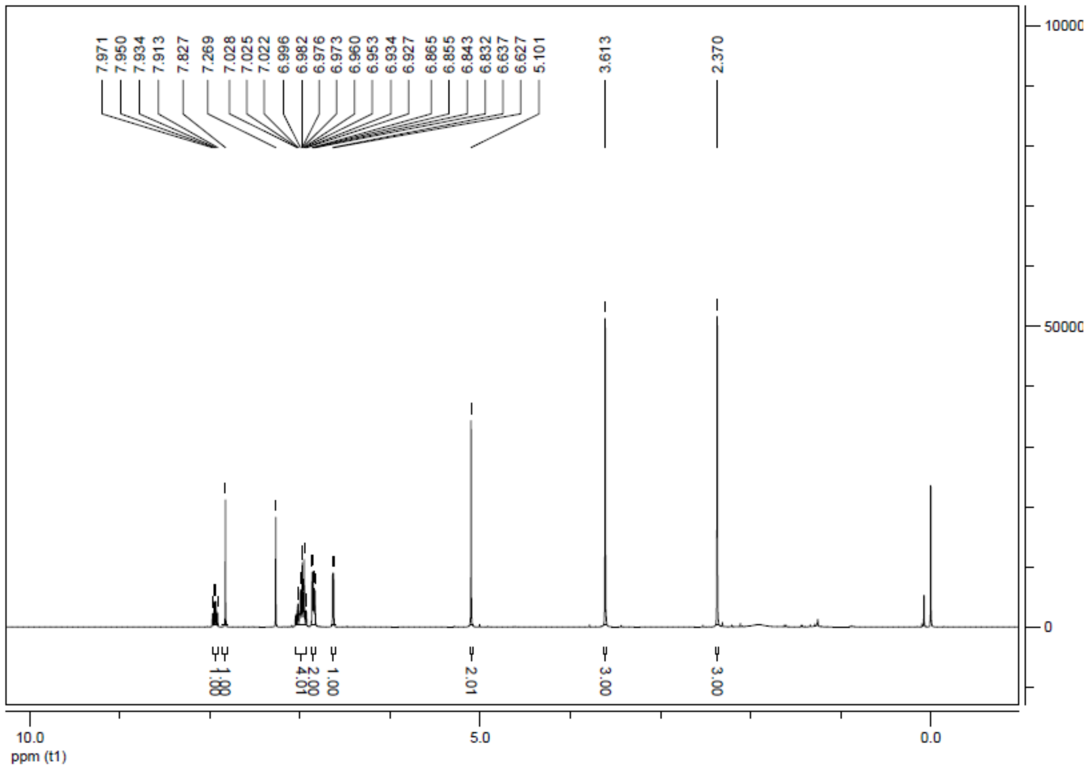

Figure S19. ^{1}H-NMR of compound **9s** (400 MHz, $CDCl_3$).

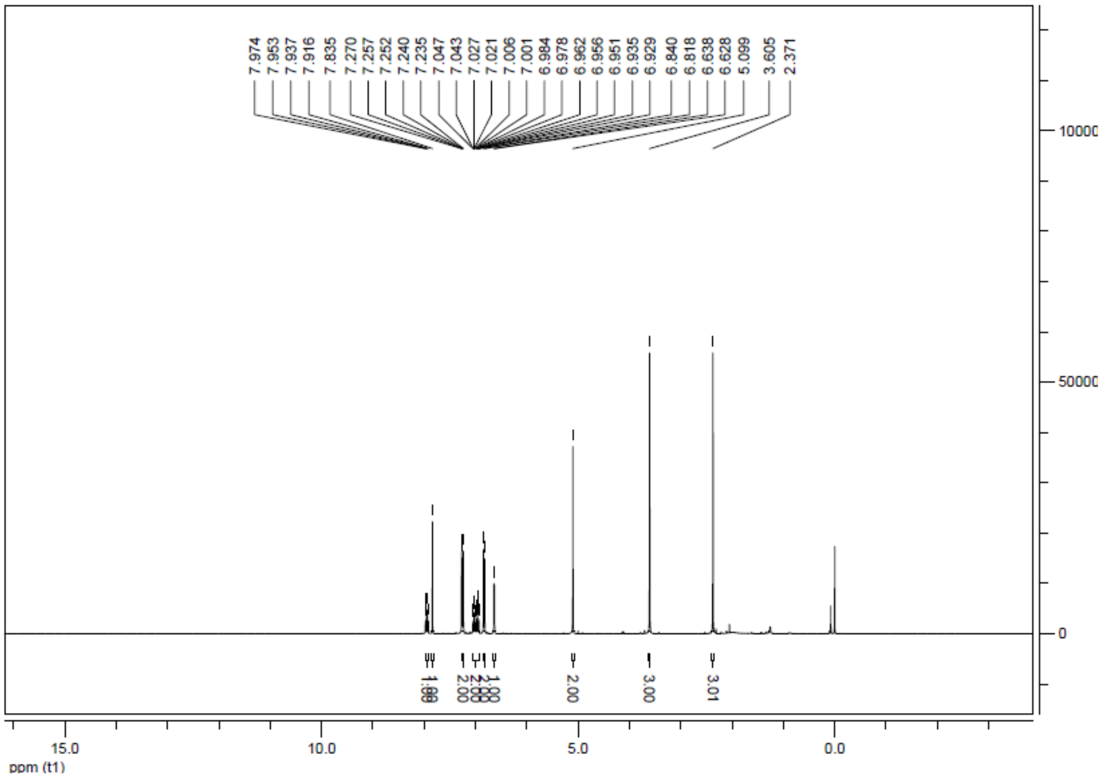

Figure S20. ^{1}H-NMR of compound **9t** (400 MHz, $CDCl_3$).

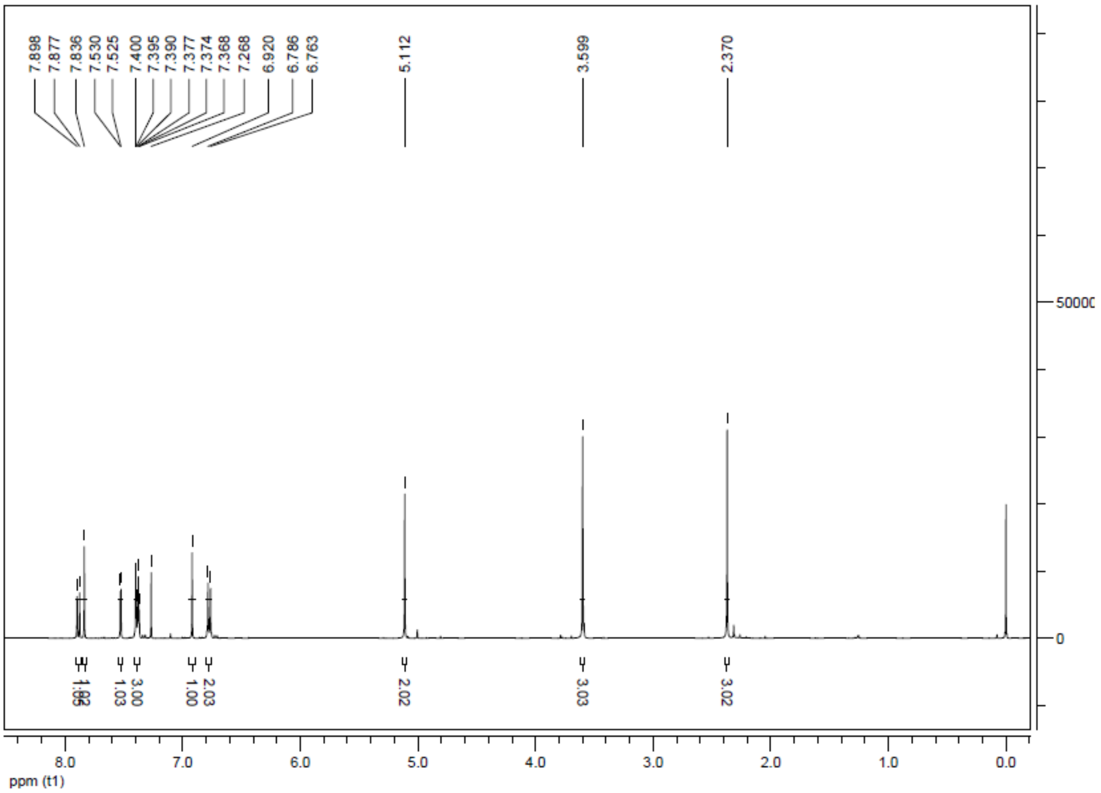

Figure S21. ^{1}H-NMR of compound **9u** (400 MHz, $CDCl_3$).

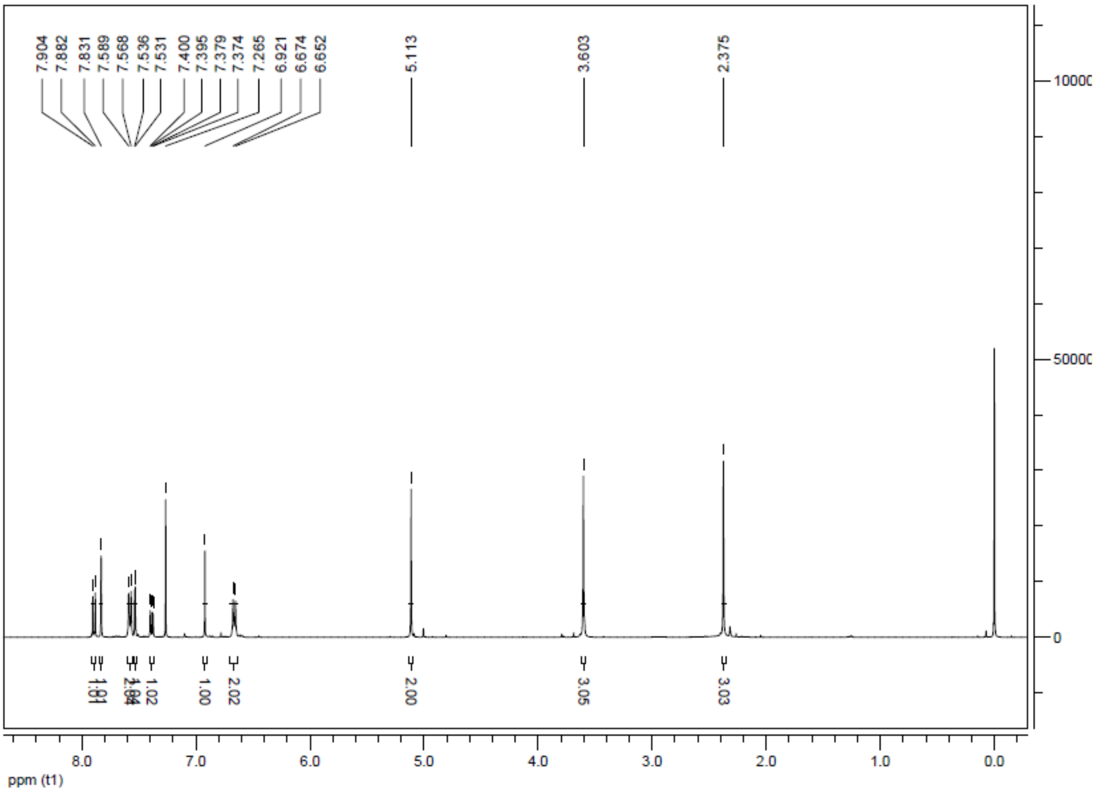

Figure S22. ^{1}H-NMR of compound **9v** (400 MHz, $CDCl_3$).

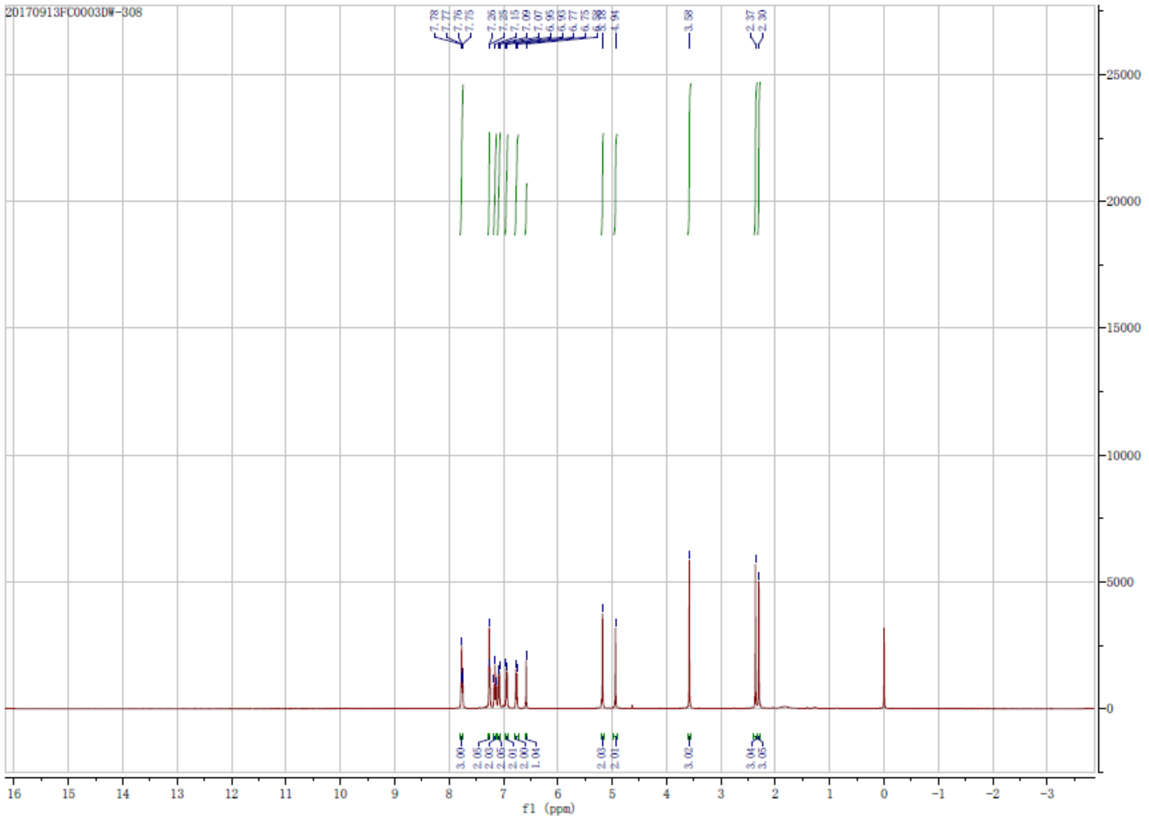

Figure S23. ^{1}H-NMR of compound **13a** (400 MHz, $CDCl_3$).

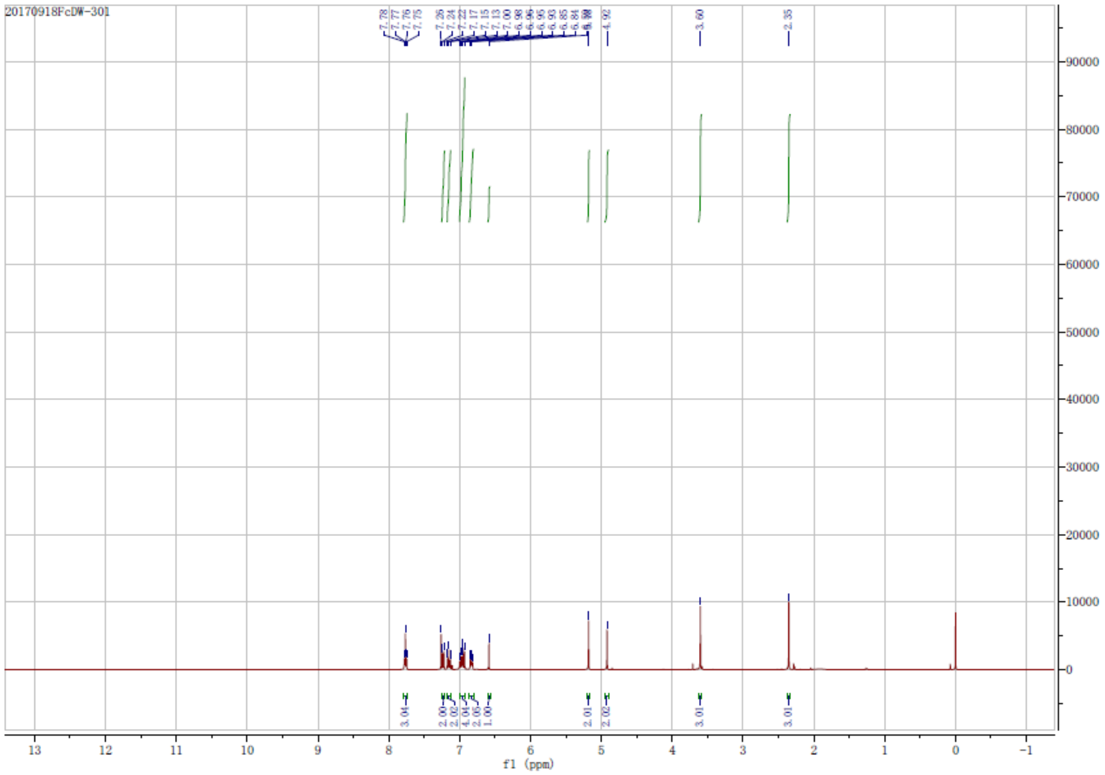

Figure S24. ^{1}H-NMR of compound **13b** (400 MHz, $CDCl_3$).

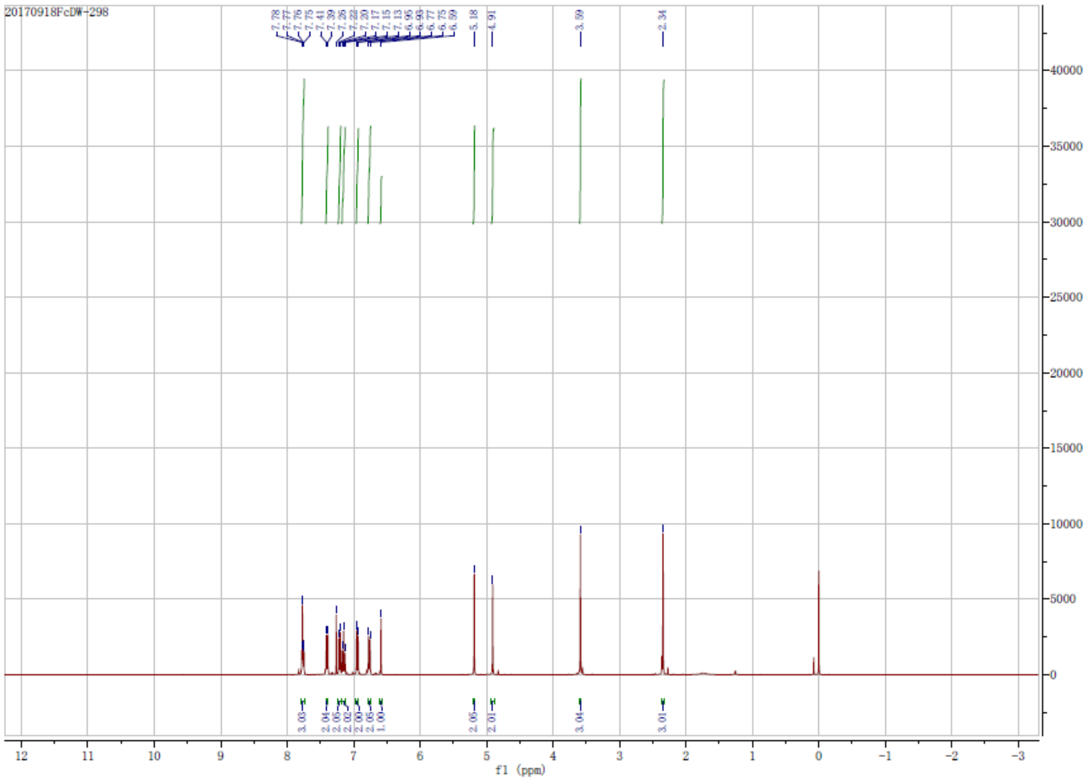

Figure S25. ^{1}H-NMR of compound **13c** (400 MHz, $CDCl_3$).

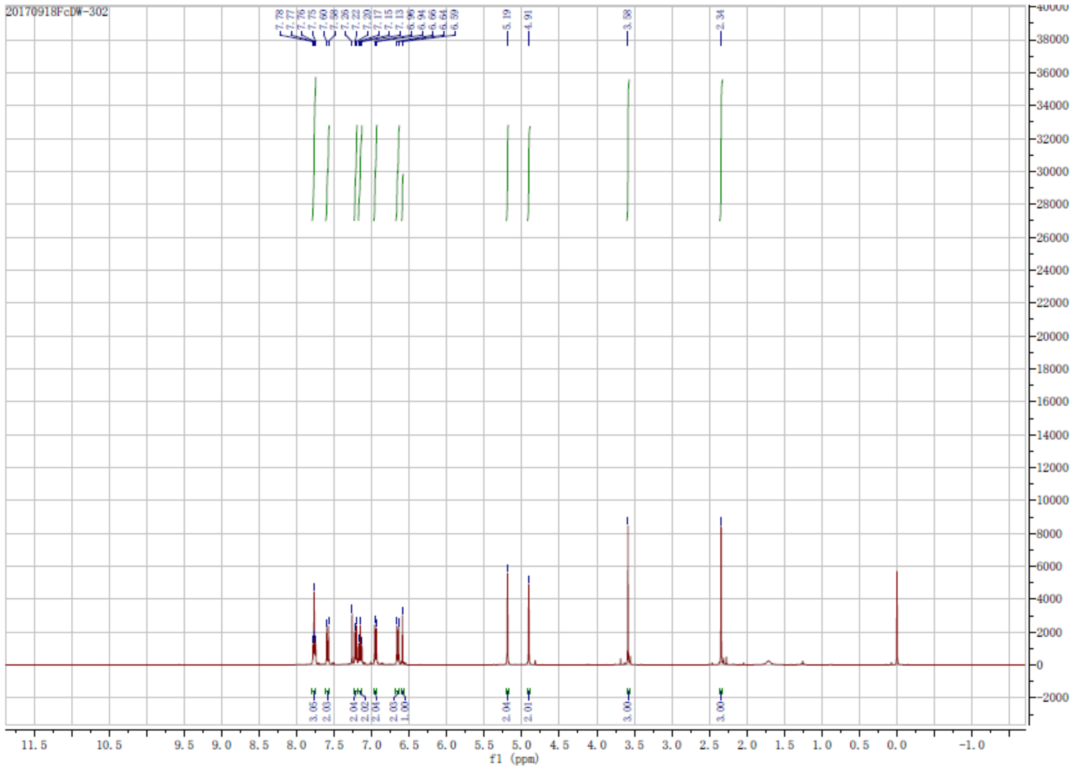

Figure S26. ^{1}H-NMR of compound **13d** (400 MHz, $CDCl_3$).

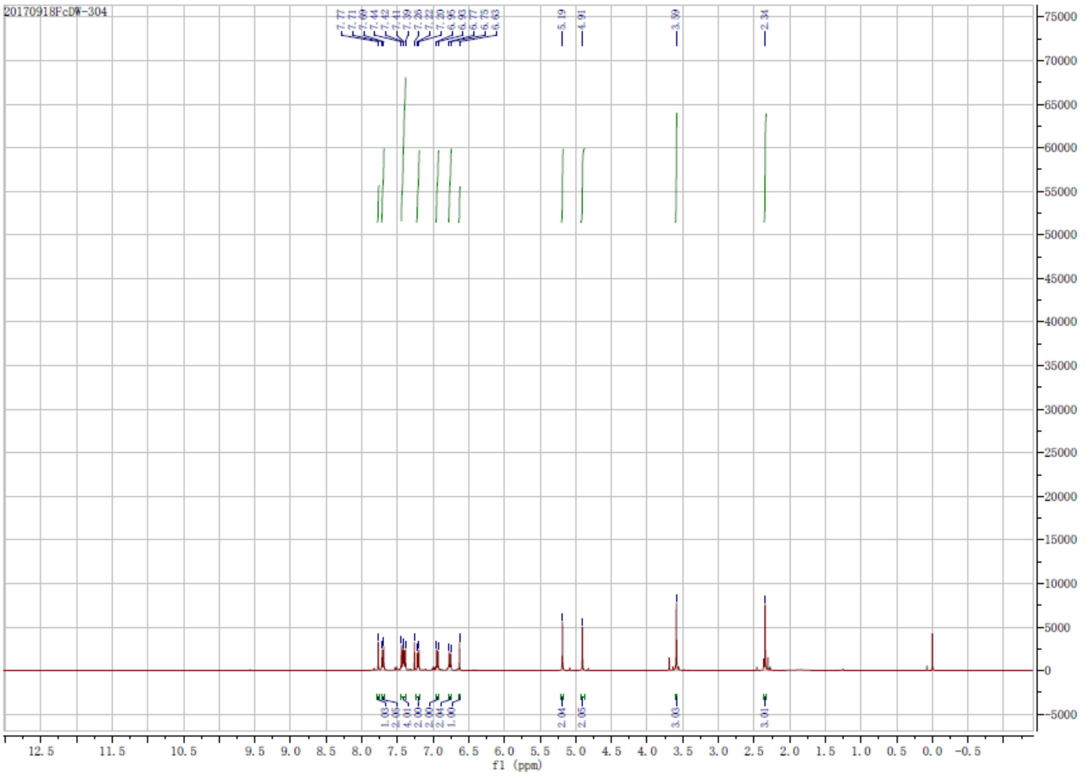

Figure S27. ^{1}H-NMR of compound **13e** (400 MHz, CDCl3).

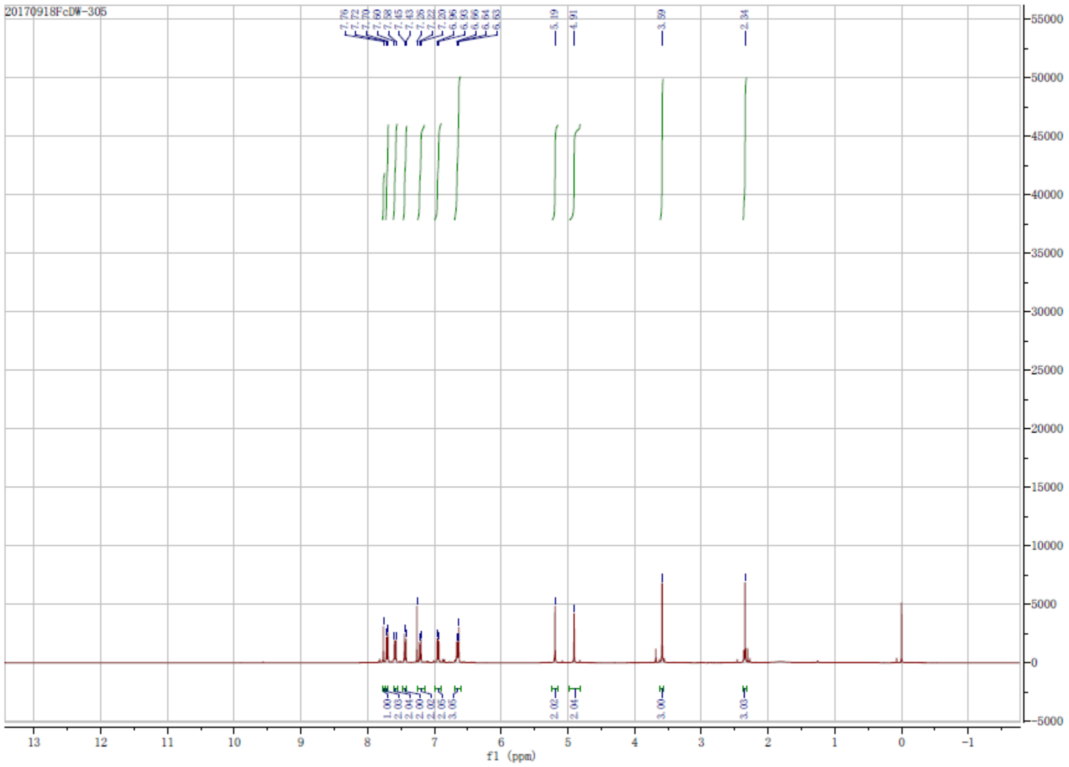

Figure S28. ^{1}H-NMR of compound **13f** (400 MHz, CDCl3).

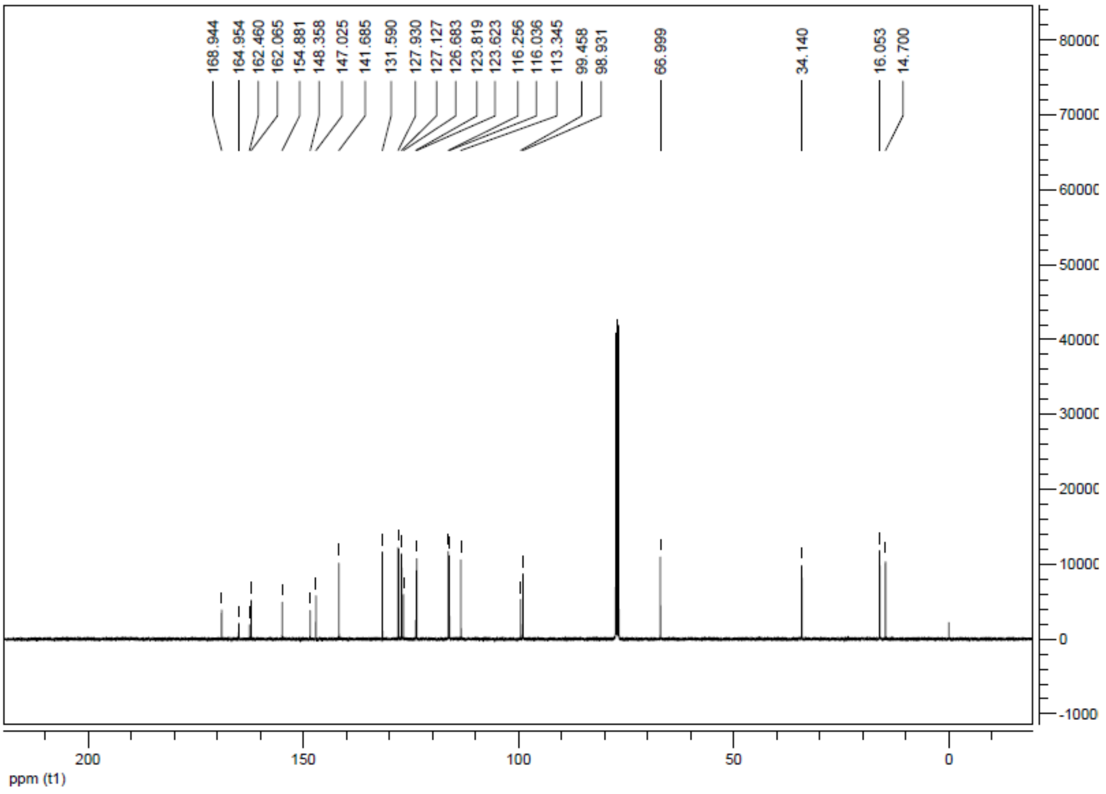

Figure S29. ^{13}C-NMR of compound **9a** (100 MHz, $CDCl_3$).

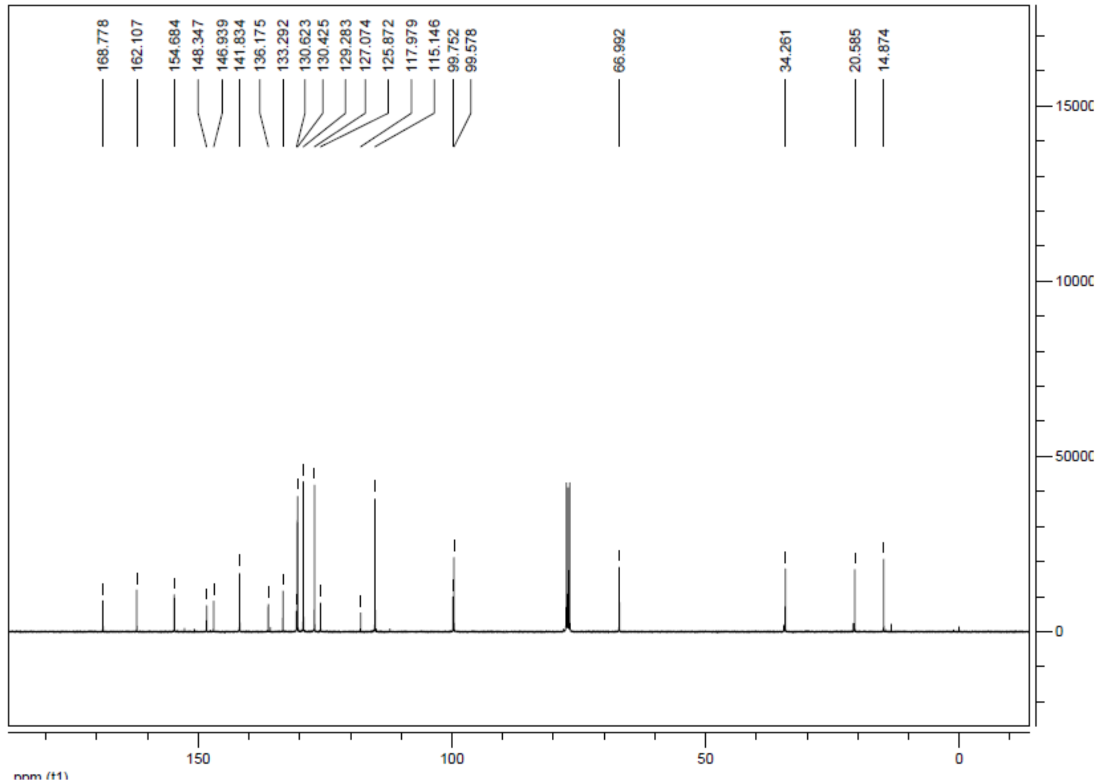

Figure S30. ^{13}C-NMR of compound **9b** (100 MHz, $CDCl_3$).

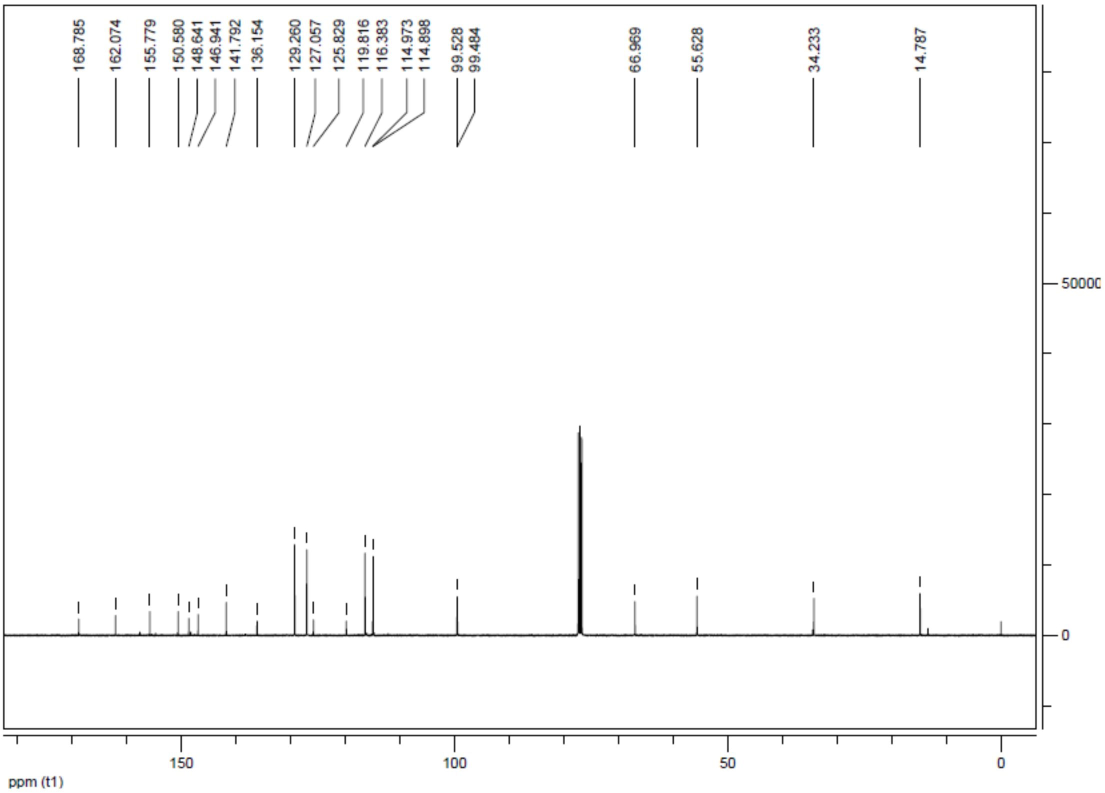

Figure S31. ^{13}C-NMR of compound **9c** (100 MHz, $CDCl_3$).

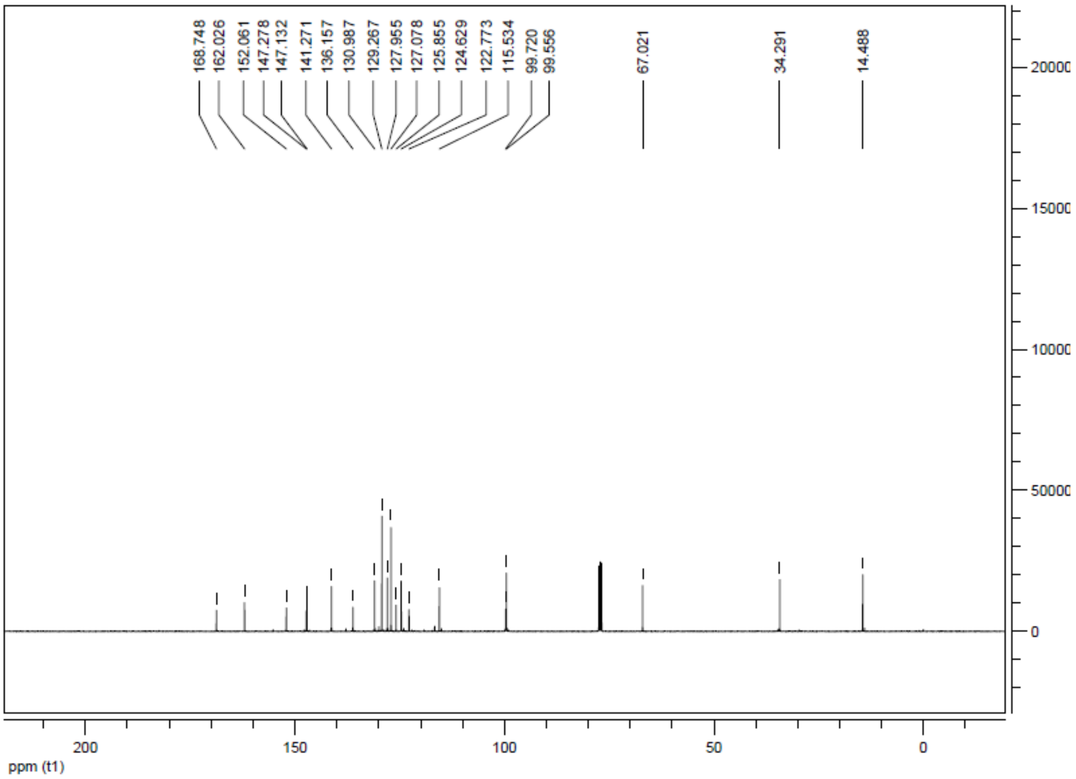

Figure S32. ^{13}C-NMR of compound **9d** (100 MHz, $CDCl_3$).

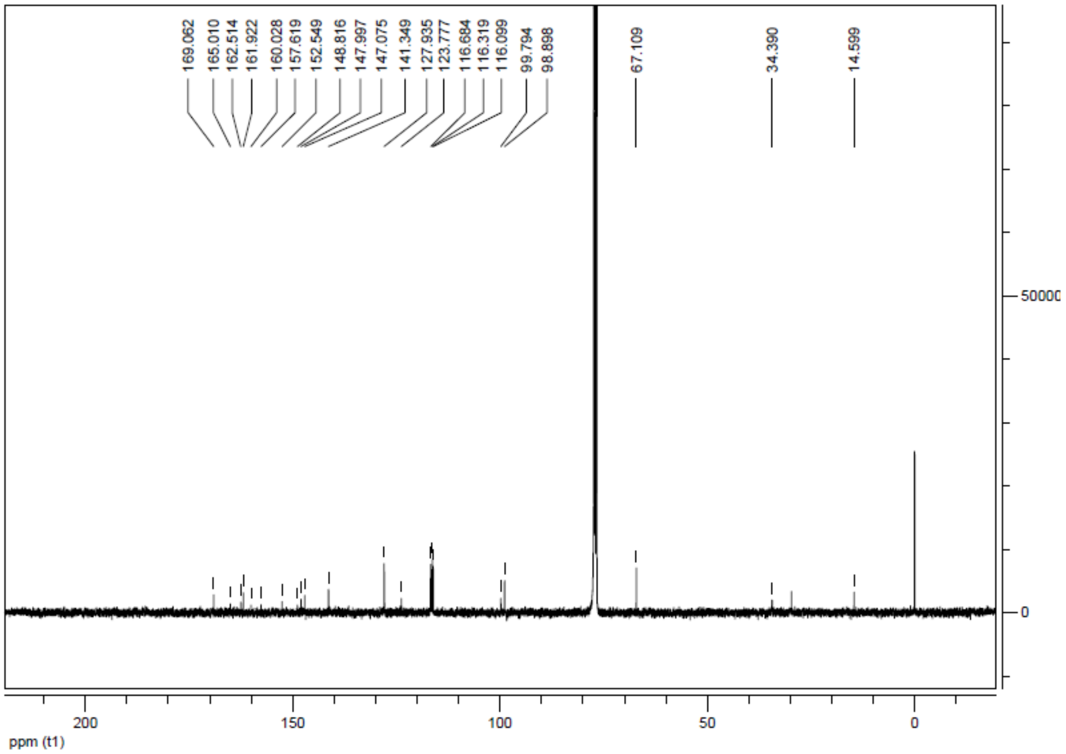

Figure S33. ^{13}C-NMR of compound **9e** (100 MHz, $CDCl_3$).

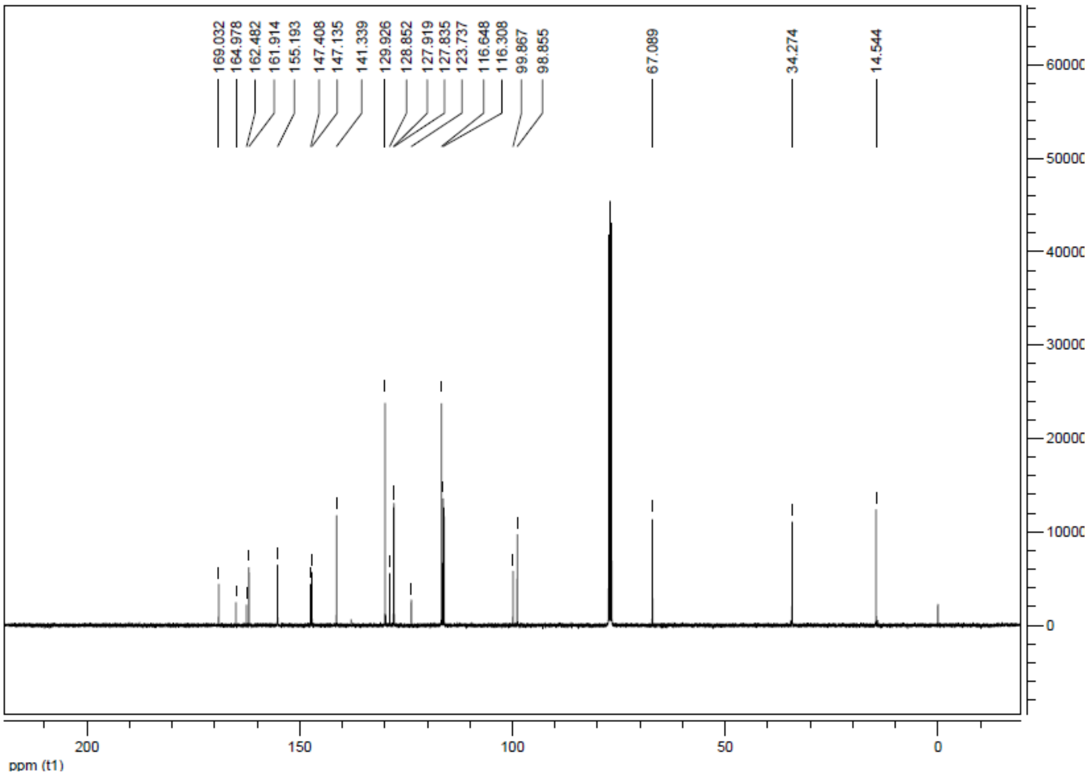

Figure S34. ^{13}C-NMR of compound **9f** (100 MHz, $CDCl_3$).

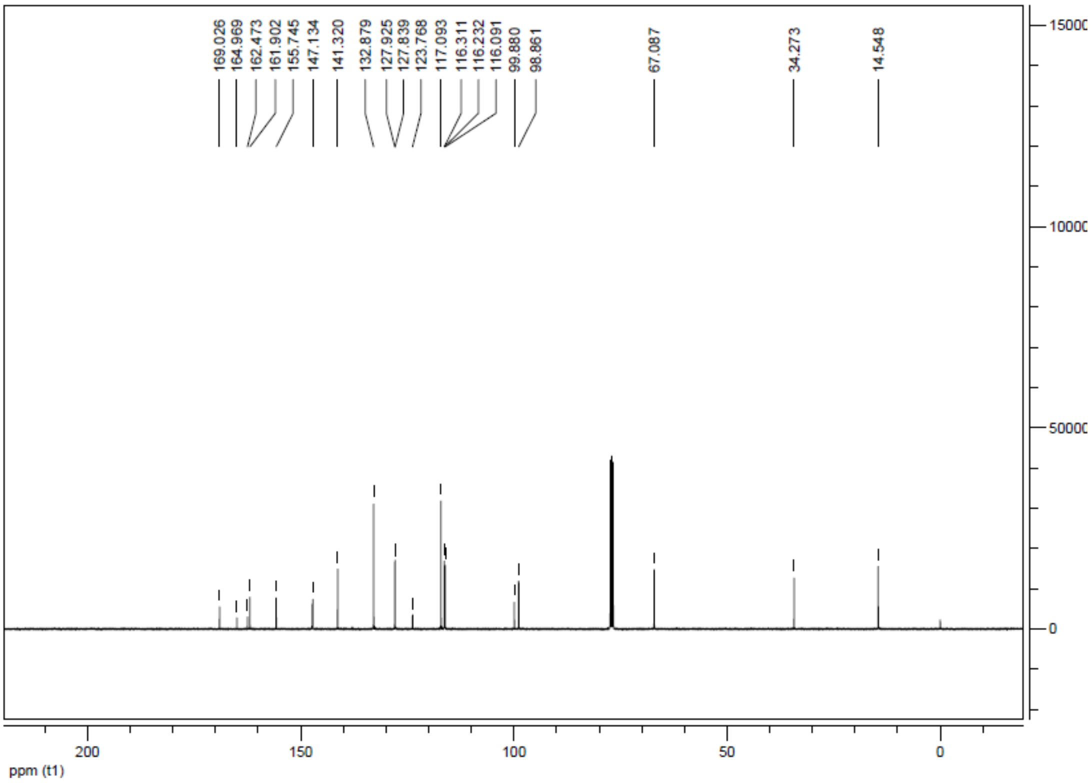

Figure S35. ^{13}C-NMR of compound **9g** (100 MHz, $CDCl_3$).

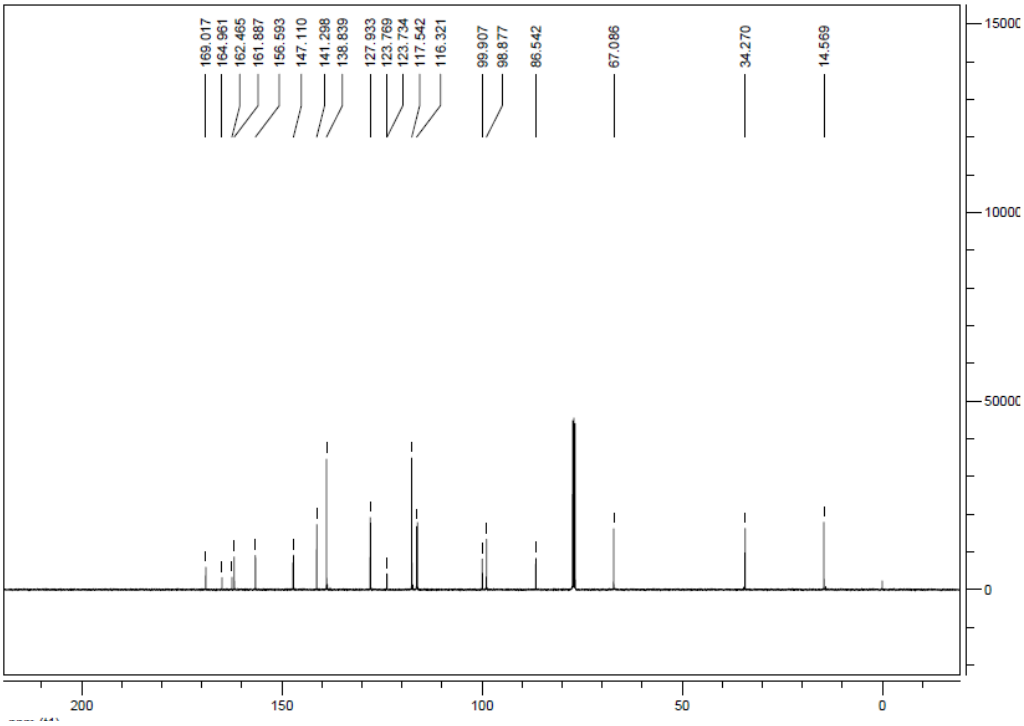

Figure S36. ^{13}C-NMR of compound **9h** (100 MHz, $CDCl_3$).

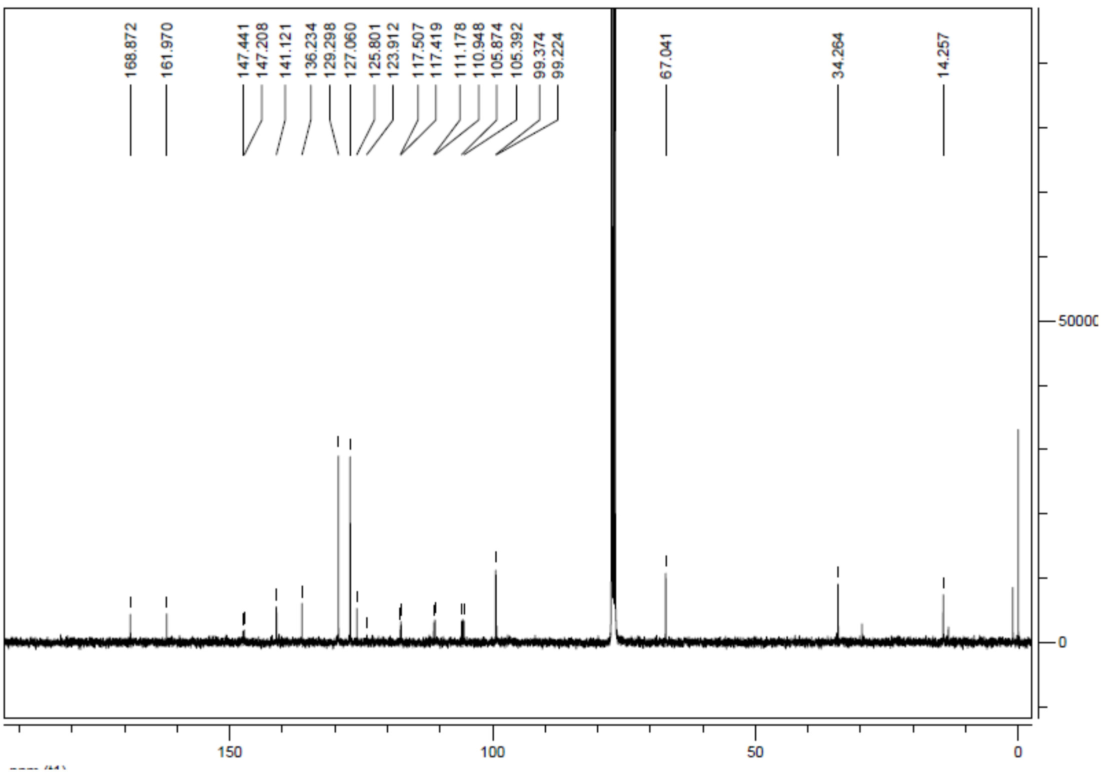

Figure S37. ^{13}C-NMR of compound **9i** (100 MHz, $CDCl_3$).

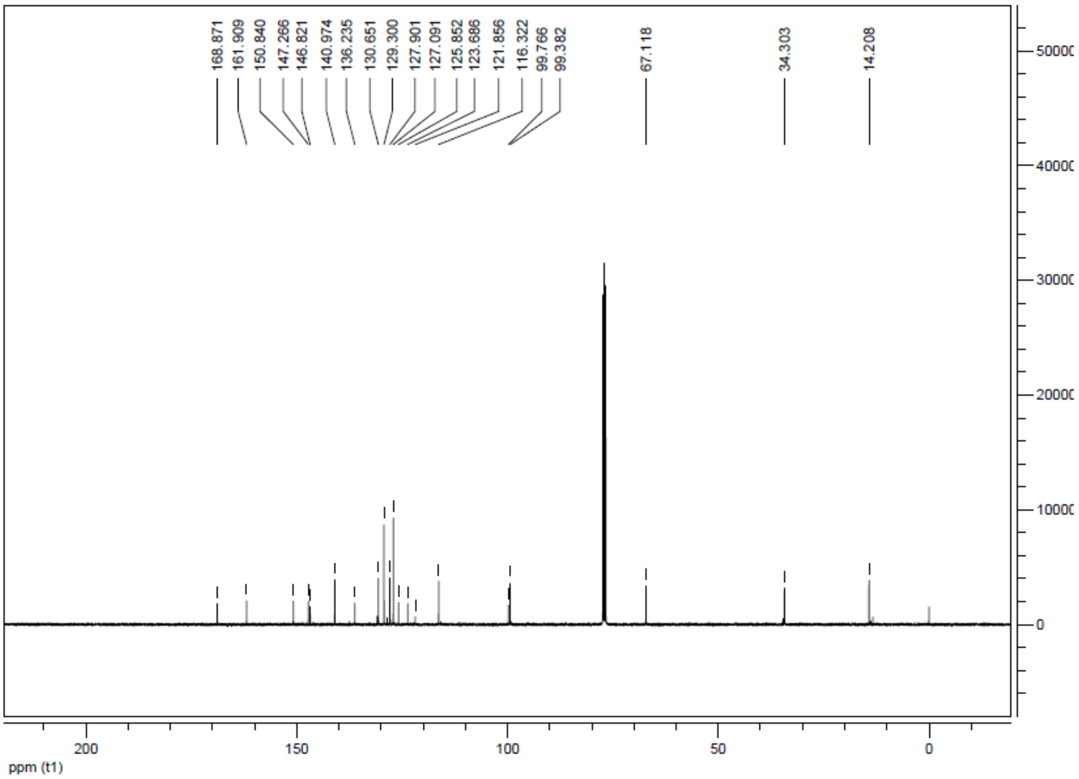

Figure S38. ^{13}C-NMR of compound **9j** (100 MHz, $CDCl_3$).

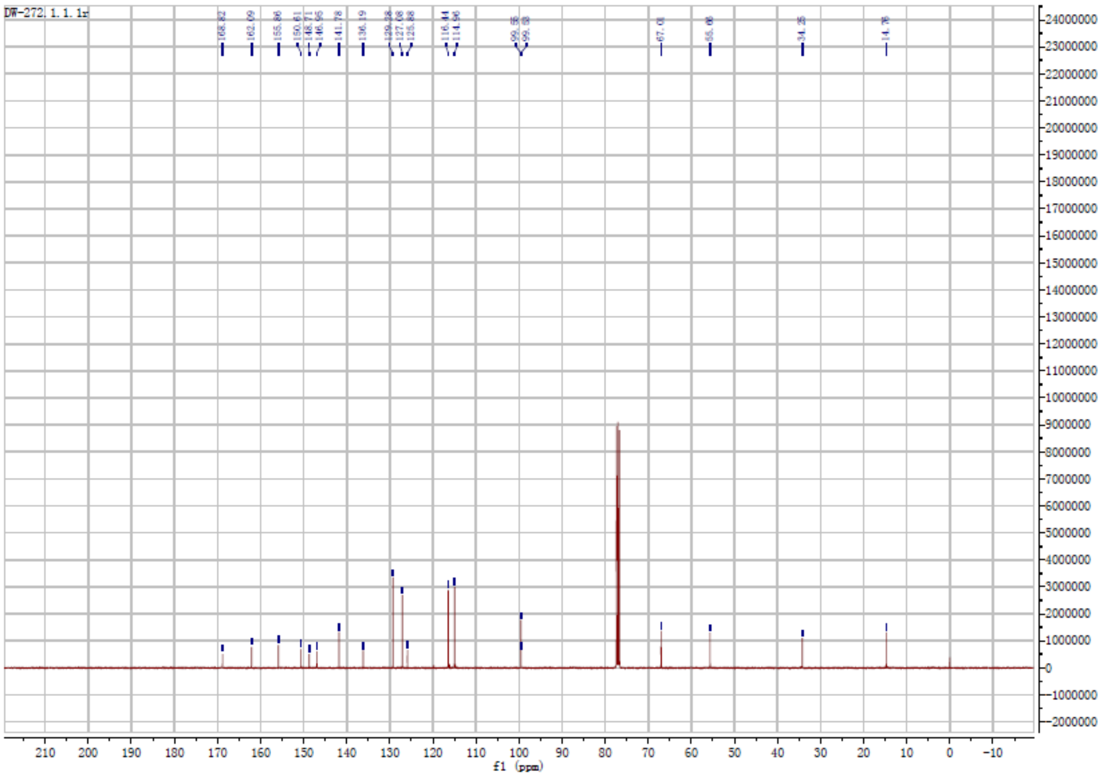

Figure S39. ^{13}C-NMR of compound **9k** (100 MHz, $CDCl_3$).

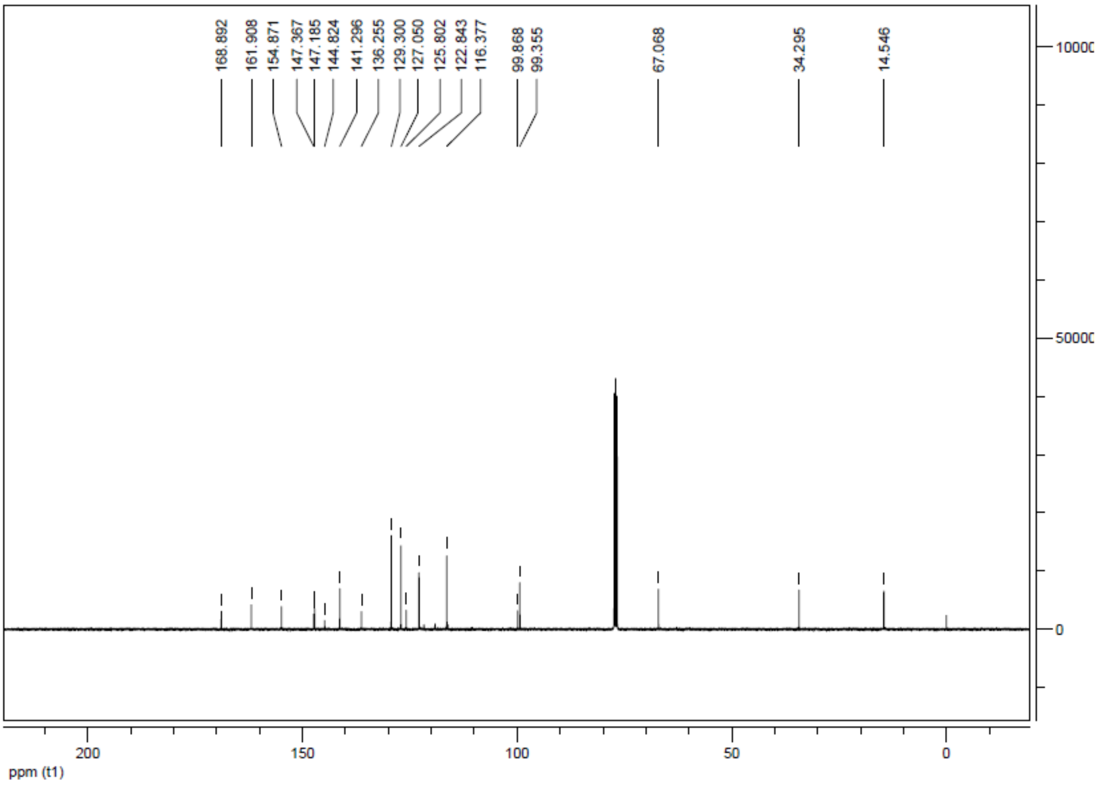

Figure S40. ^{13}C-NMR of compound **9l** (100 MHz, $CDCl_3$).

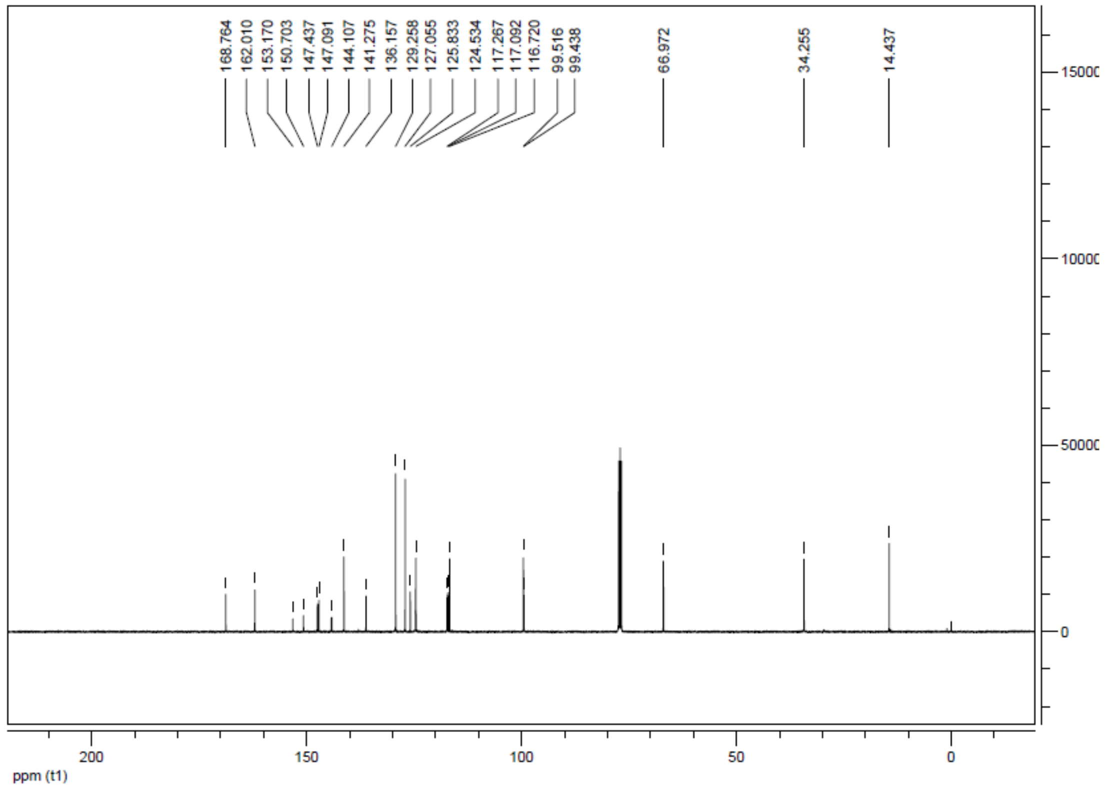

Figure S41. ^{13}C-NMR of compound **9m** (100 MHz, $CDCl_3$).

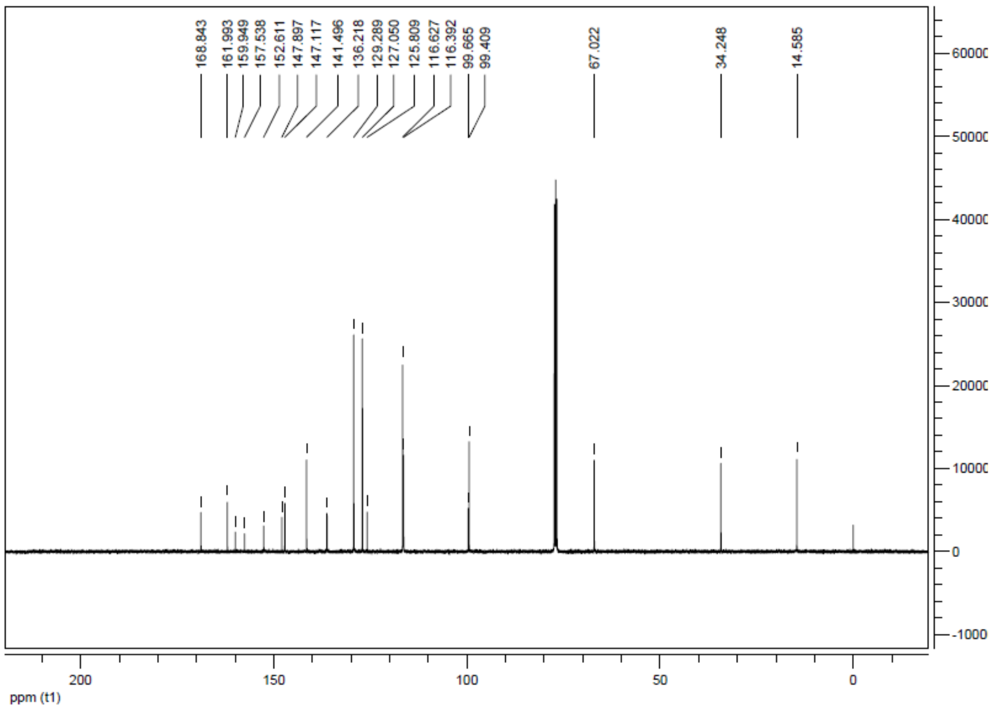

Figure S42. ^{13}C-NMR of compound **9n** (100 MHz, $CDCl_3$).

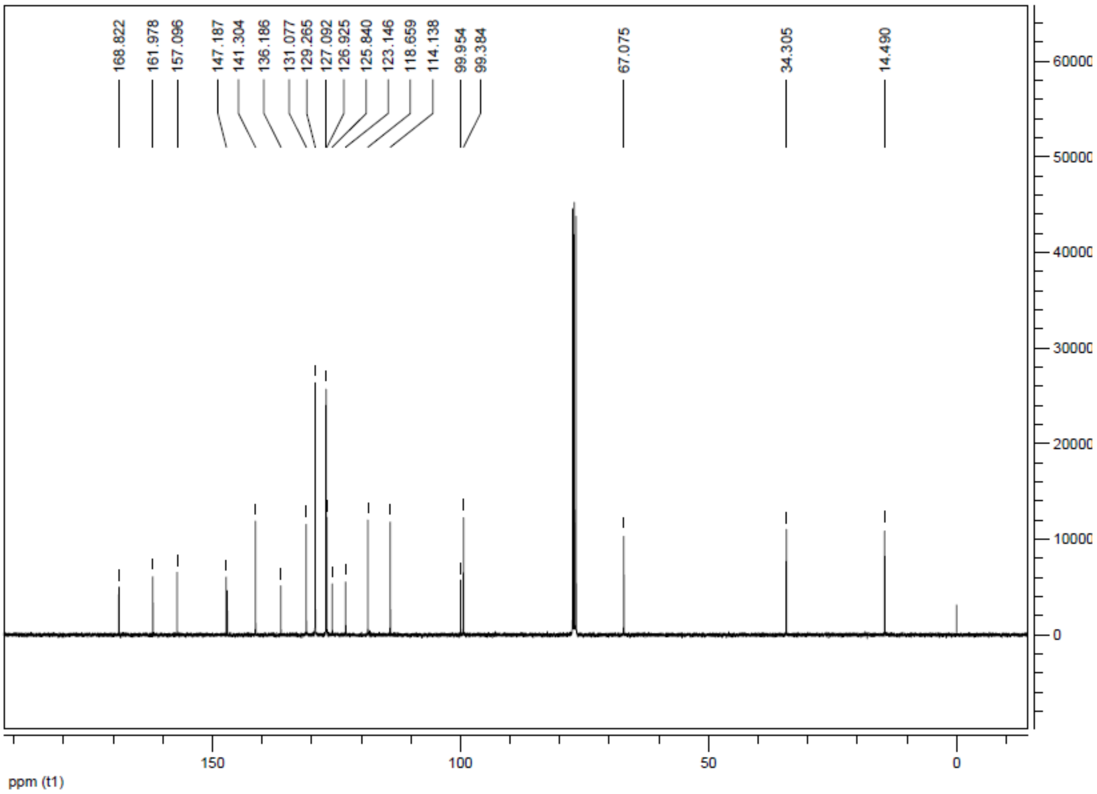

Figure S43. ^{13}C-NMR of compound **9o** (100 MHz, $CDCl_3$).

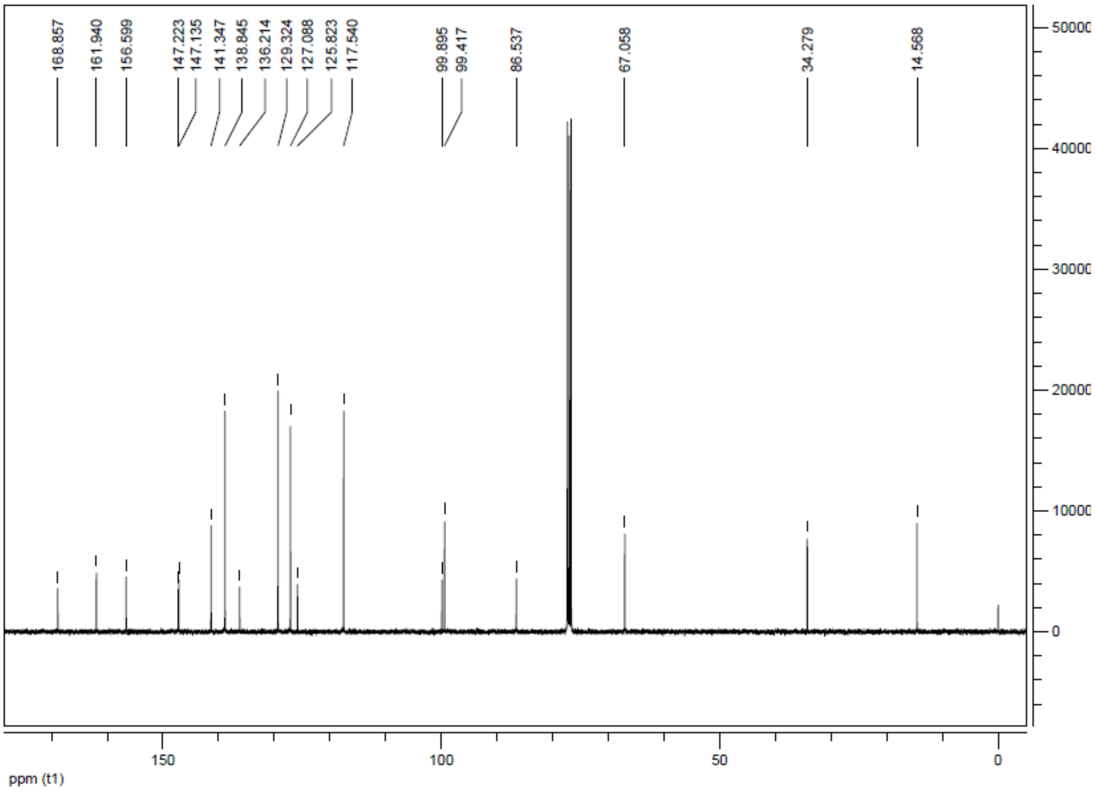

Figure S44. ^{13}C-NMR of compound **9p** (100 MHz, $CDCl_3$).

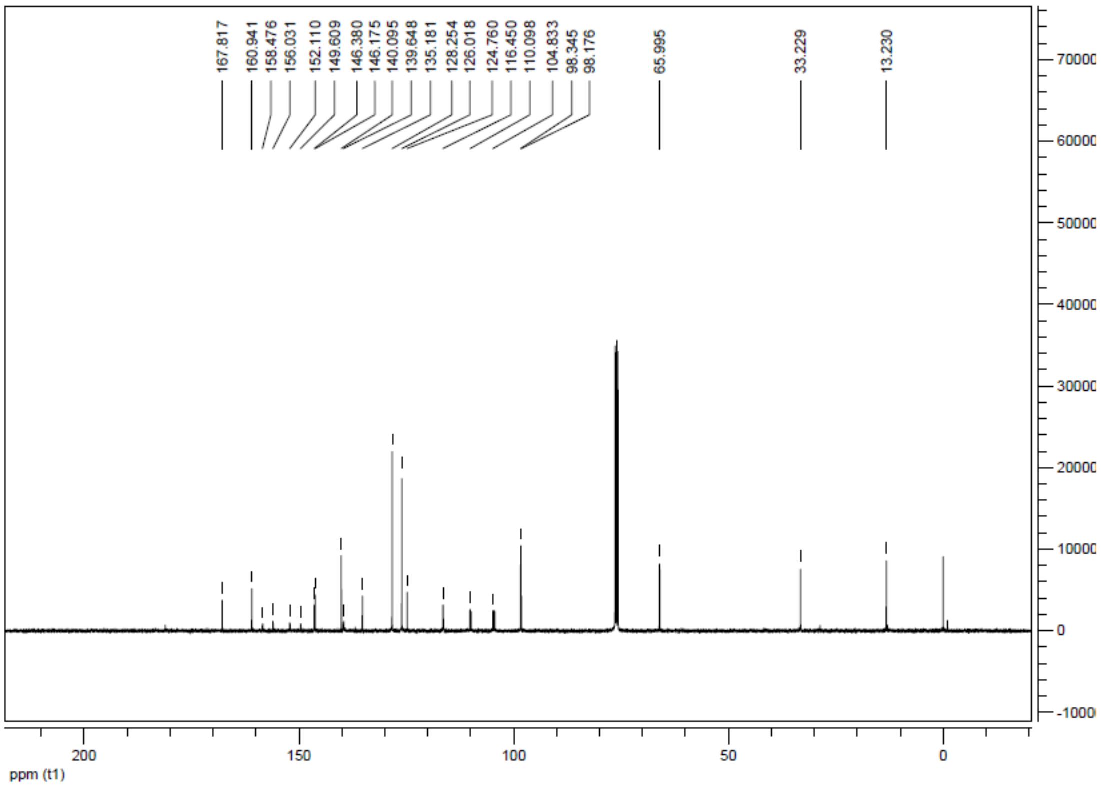

Figure S45. ^{13}C-NMR of compound **9q** (100 MHz, $CDCl_3$).

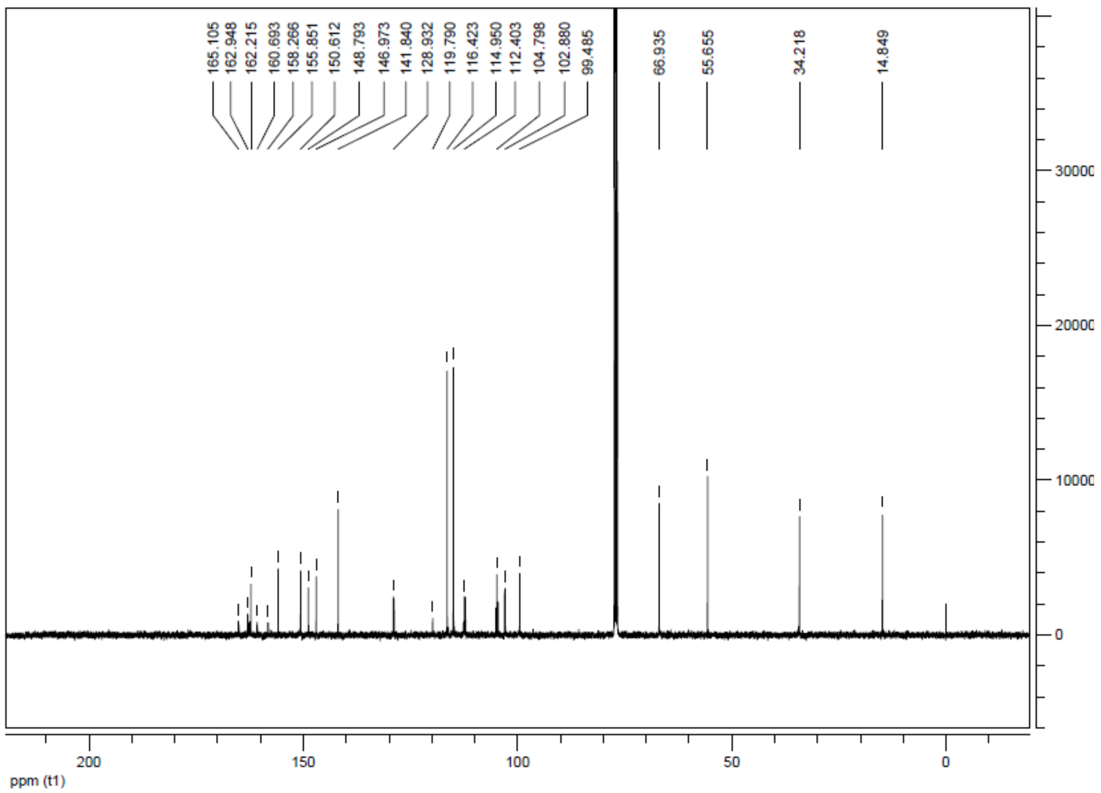

Figure S46. ^{13}C-NMR of compound **9r** (100 MHz, $CDCl_3$).

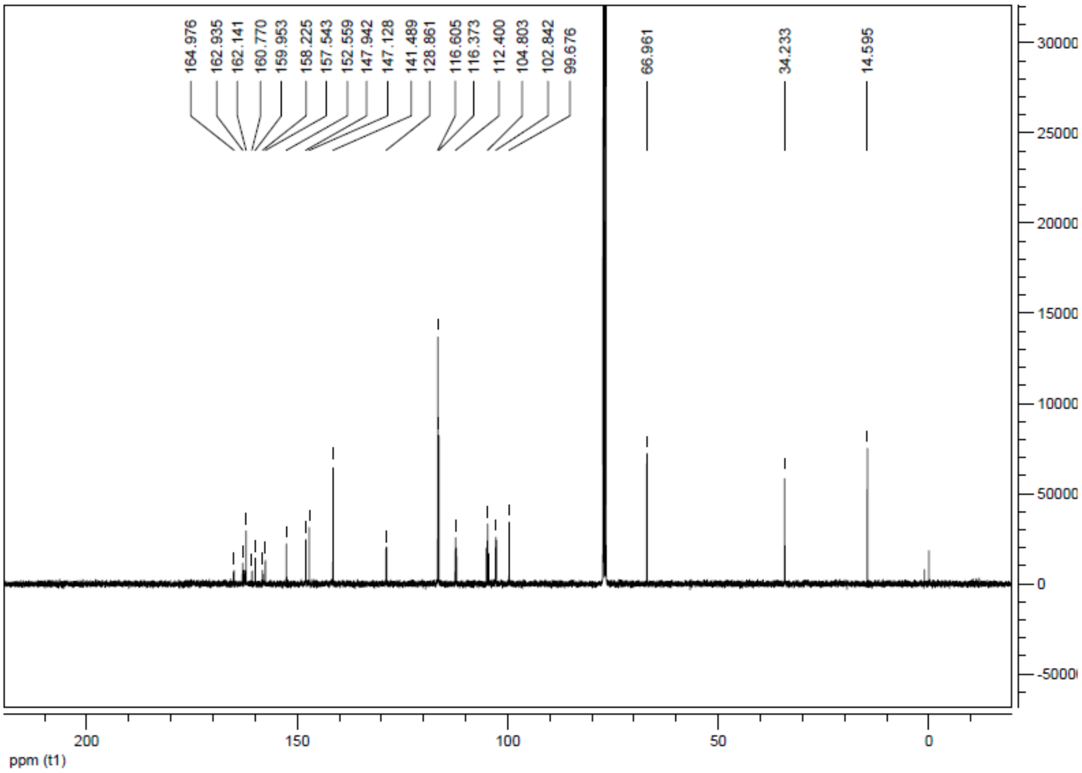

Figure S47. ^{13}C-NMR of compound **9s** (100 MHz, $CDCl_3$).

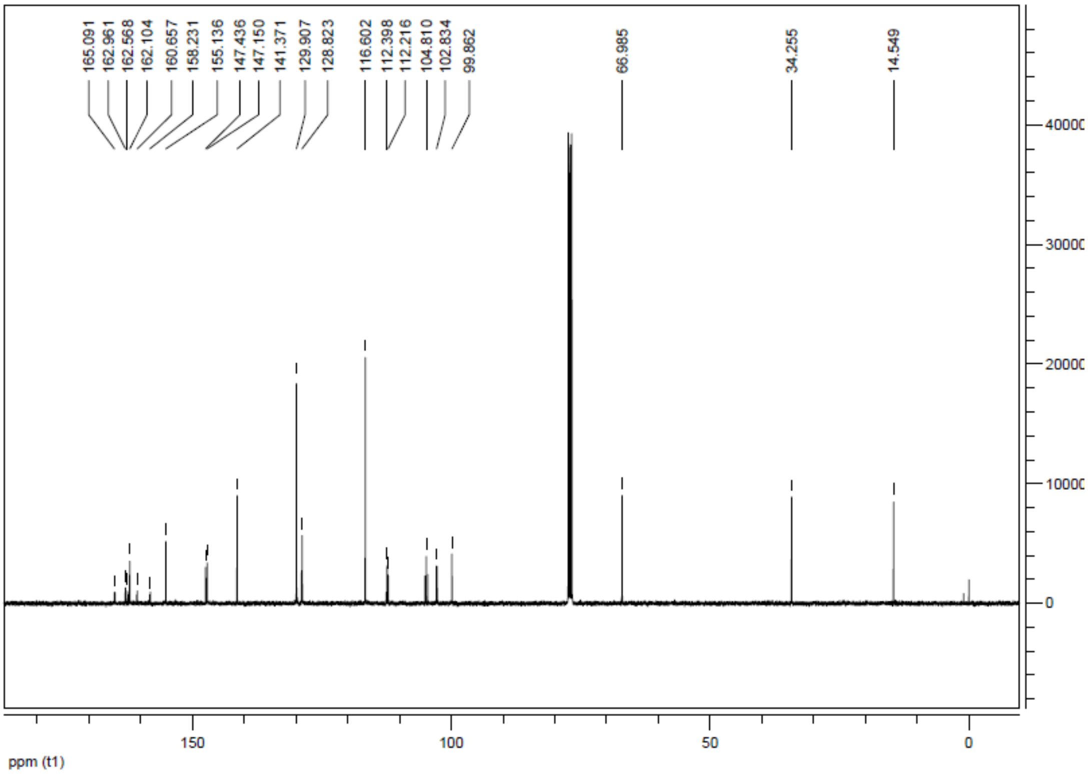

Figure S48. ^{13}C-NMR of compound **9t** (100 MHz, $CDCl_3$).

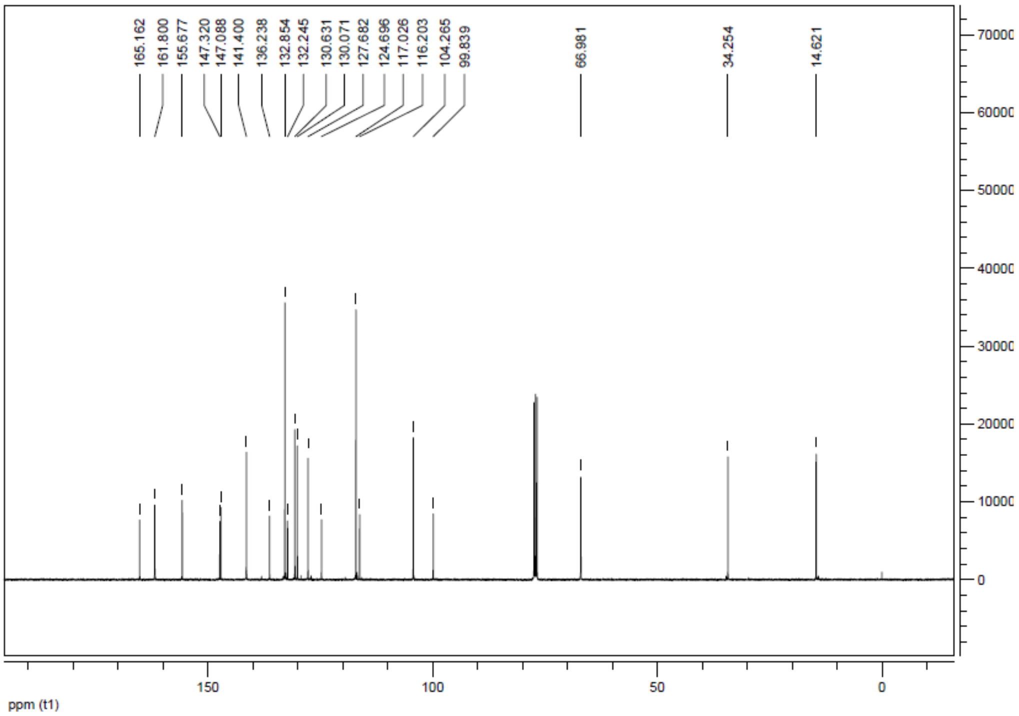

Figure S49. ^{13}C-NMR of compound **9u** (100 MHz, $CDCl_3$).

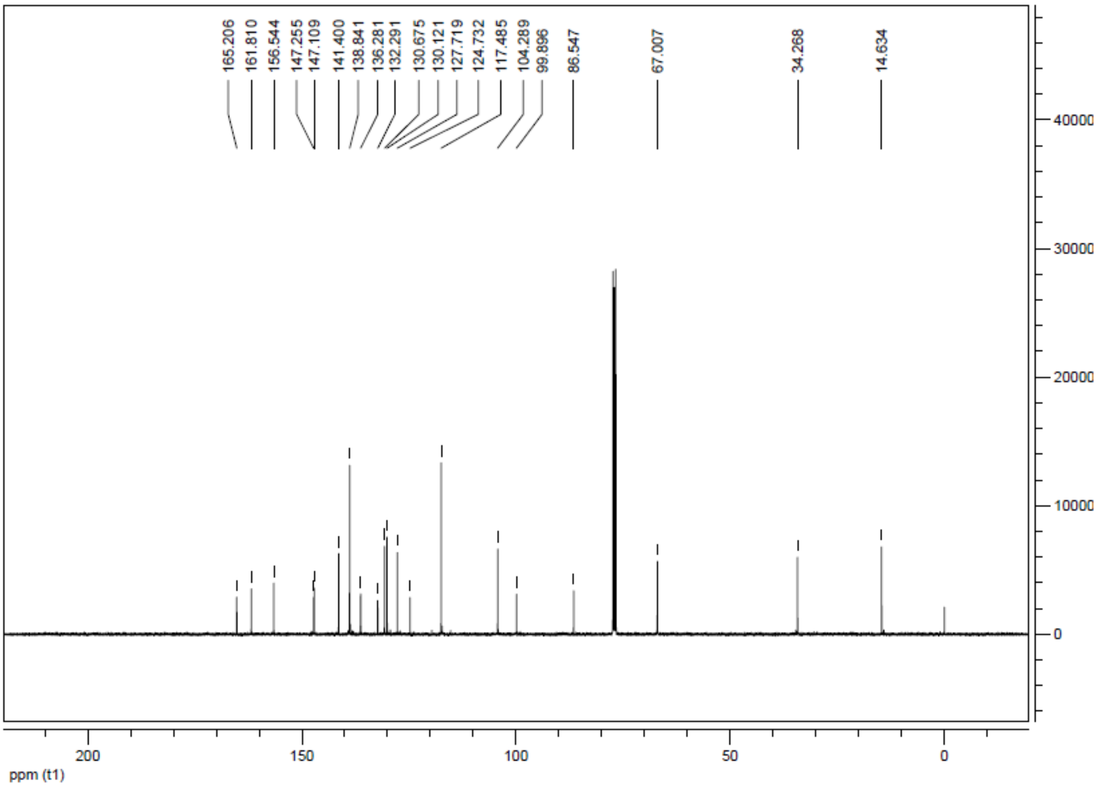

Figure S50. ^{13}C-NMR of compound **9v** (100 MHz, $CDCl_3$).

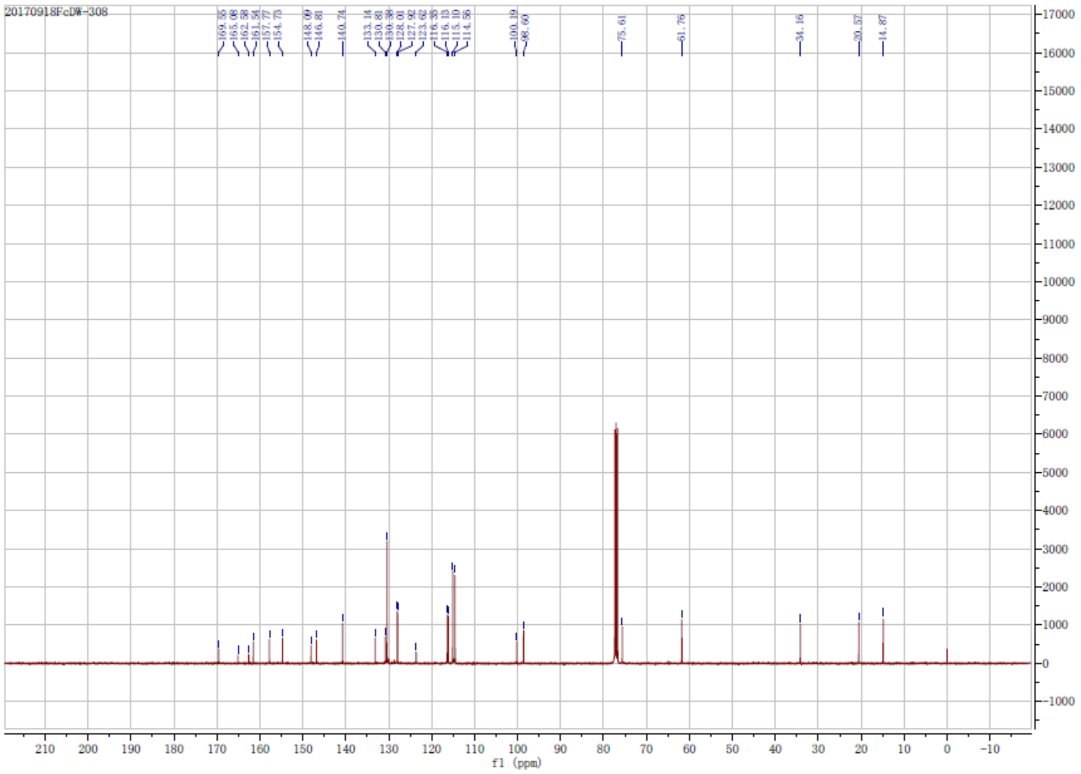

Figure S51. ^{13}C-NMR of compound **13a** (100 MHz, $CDCl_3$).

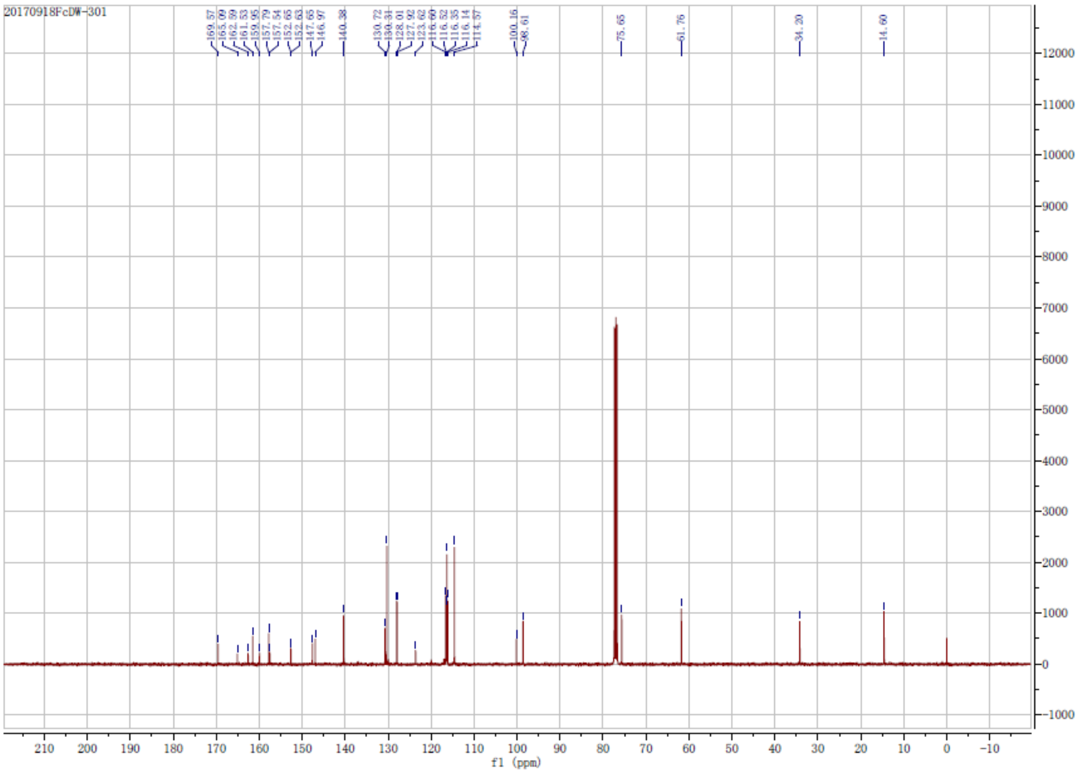

Figure S52. ^{13}C-NMR of compound **13b** (100 MHz, $CDCl_3$).

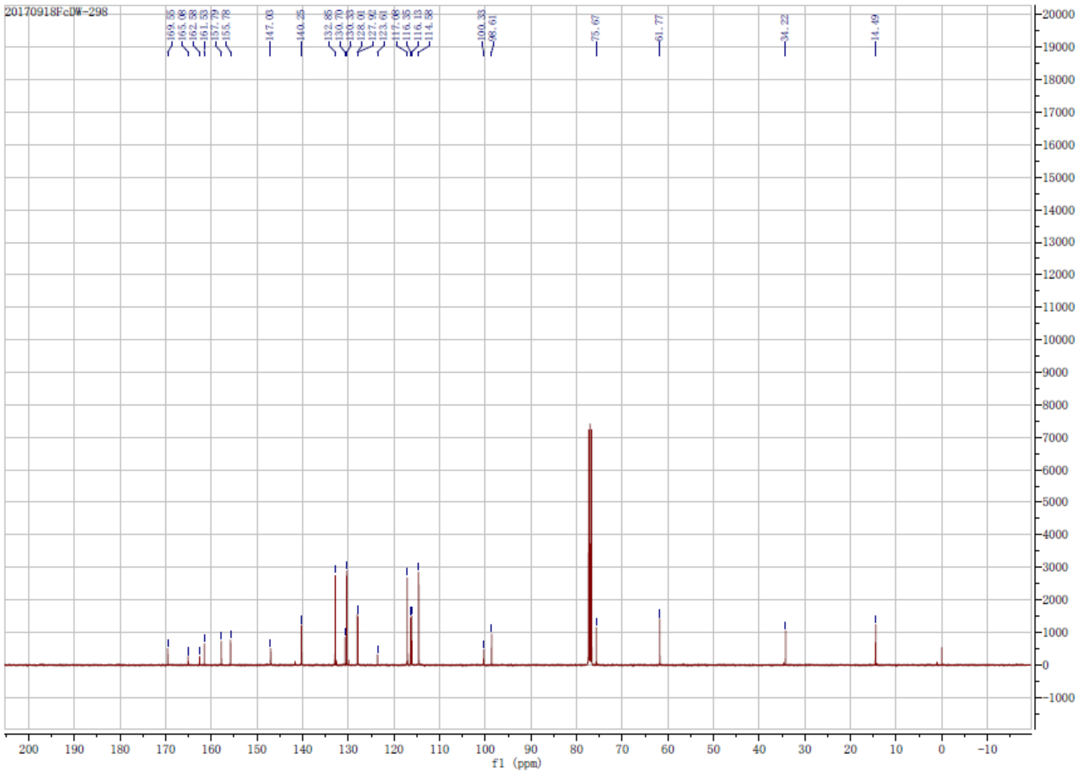

Figure S53. ^{13}C-NMR of compound **13c** (100 MHz, $CDCl_3$).

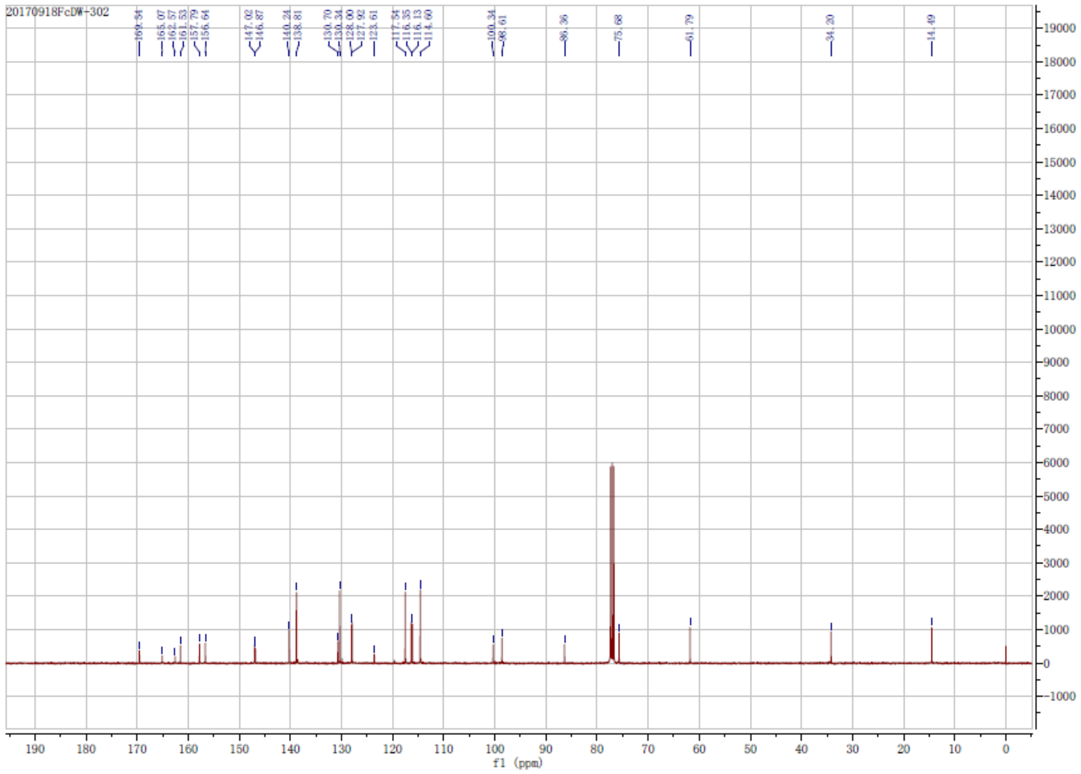

Figure S54. ^{13}C-NMR of compound **13d** (100 MHz, $CDCl_3$).

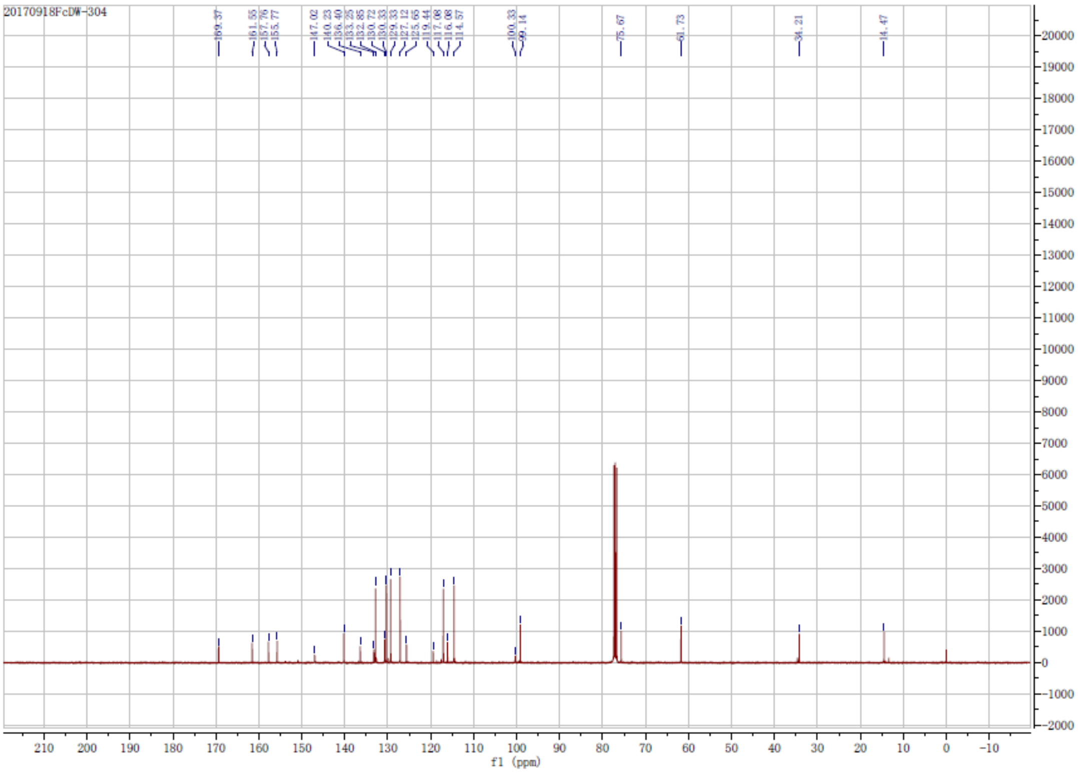

Figure S55. ^{13}C-NMR of compound **13e** (100 MHz, $CDCl_3$).

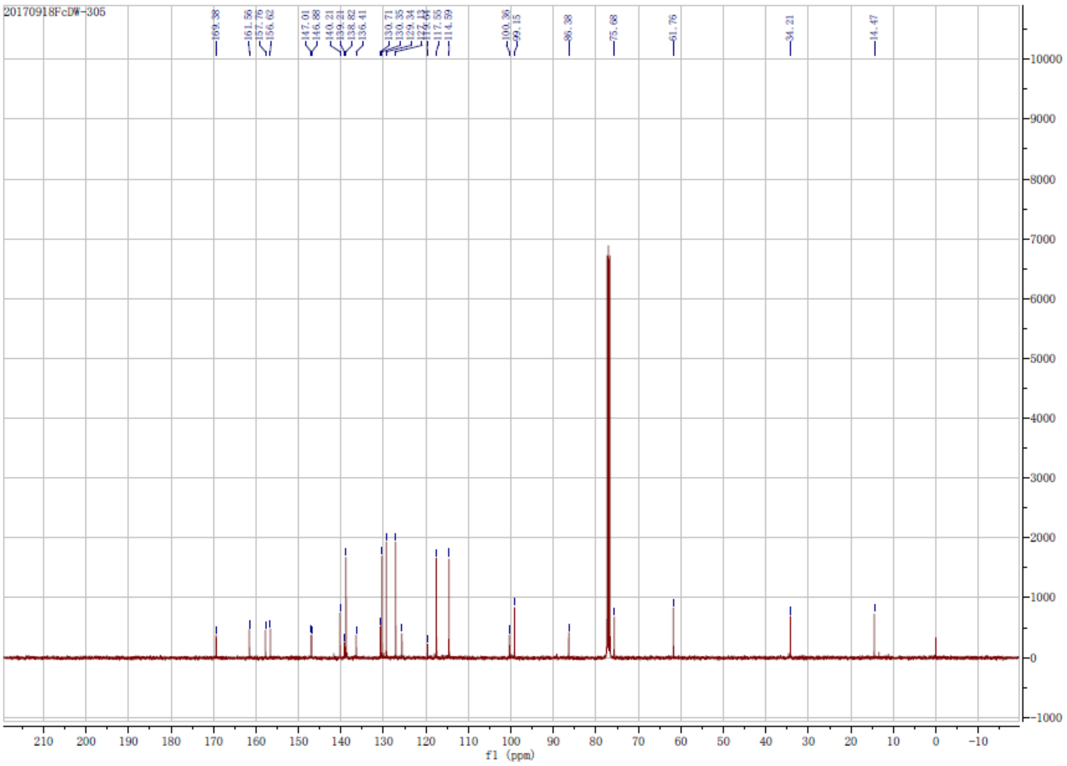

Figure S56. ^{13}C-NMR of compound **13f** (100 MHz, $CDCl_3$).
